# Effector and regulatory dendritic cells display distinct patterns of miRNA expression

**DOI:** 10.1002/iid3.165

**Published:** 2017-05-12

**Authors:** Vincent Lombardi, Sonia Luce, Hélène Moussu, Lise Morizur, Claire Gueguen, Catherine Neukirch, Sylvie Chollet‐Martin, Laurent Mascarell, Michel Aubier, Véronique Baron‐Bodo, Philippe Moingeon

**Affiliations:** ^1^ Research Department Stallergenes Greer Antony France; ^2^ Department of Pulmonology, University Hospital Paris Nord Val de Seine, Hospital Bichat AP‐HP, INSERM UMR 1152 University Hospital Department FIRE Paris France; ^3^ Department of Immunology, INSERM UMRS996 Bichat Claude Bernard Hospital Paris France

**Keywords:** Allergic rhinitis, dendritic cells, miRNA

## Abstract

**Introduction:**

MicroRNAs (miRNAs) contribute to the regulation of dendritic cell (DC) polarization, thereby influencing the balance of adaptive immune responses. Herein, we studied the expression of miRNAs in polarized DCs and analyzed whether expression of these miRNAs could be associated with allergic rhinitis and allergen immunotherapy (AIT) outcome.

**Method:**

Using specific culture conditions, we differentiated immature human monocyte‐derived DCs into DC1, DC2, and DCreg subsets (supporting the differentiation of T_H_1, T_H_2 or regulatory T cells, respectively). Profiling of miRNA expression was performed in these DC subpopulations using microarrays. Levels of miRNAs specific for polarized DCs were then evaluated in a cohort of 58 patients with allergic rhinitis and 25 non‐allergic controls, as well as in samples from 30 subjects treated with sublingual grass pollen tablets or placebo for four months.

**Results:**

We successfully identified 16 miRNAs differentially regulated between immature DCs, DC1, DC2, and DCreg cells. In allergic rhinoconjunctivitis patients, the expression of two of those miRNAs (miR‐132 and miR‐155), was down‐regulated compared to non‐allergic individuals. However, the levels of these miRNAs were not significantly modified following four months of grass pollen immunotherapy.

**Conclusions:**

Studying polarized DCs and clinical samples from subjects with or without allergic rhinoconjunctivitis, we demonstrated that the expression of two miRNAs linked to effector DCs (i.e., DC1 and/or DC2 cells), was reduced in the blood of patients with allergic rhinoconjunctivitis. Nevertheless, these miRNAs did not represent relevant biomarkers to predict or follow‐up AIT efficacy.

## Introduction

Depending upon their state of differentiation, dendritic cells (DCs) control the orientation of adaptive immune responses, thus, representing a potential source of markers indicative of the overall polarization of the immune system 1, 2, 3, 4. In this regard, we identified recently specific molecular signatures associated with human monocyte‐derived DCs (MoDCs) polarized in vitro into either DC1, DC2, or DCreg cells (i.e., DCs driving the differentiation of naive CD4^+^ T cells into T_H_1, T_H_2, and Treg cells, respectively 5, 6). Using such molecular signatures of DC polarization, we demonstrated that successful allergen immunotherapy (AIT) in grass pollen allergic patients is associated with the up‐regulation of markers related to DCreg cells concomitantly with a decrease of those linked to DC2s in the patients’ blood 5, 6.

To further extend those results, we investigated herein whether differentially polarized subsets of DCs express distinct patterns of microRNAs (miRNAs). MiRNAs are small non‐coding RNA molecules which contribute to the post‐transcriptional regulation of gene expression by triggering the degradation of target messenger RNAs. As such, miRNAs are involved in the regulation of inflammatory responses 7, with some evidence for miRNA dysregulation occurring in allergy and asthma 7, 8, 9. Whereas patterns of miRNA expression have mostly been studied in T and B lymphocytes, recent evidence suggests as well that miRNAs could contribute to the regulation of innate immune responses and even to innate immune memory associated with cells such as monocytes and DCs 8, 10. In particular, miRNAs have been shown to be important regulators of lineage commitment, maturation, antigen presentation, and cytokine production for DCs 10. We provide evidence in the present study that some miRNAs are differentially expressed in DC1, DC2, and DCreg subsets and discuss their use as potential markers for allergic inflammation and AIT 11.

## Methods

### Polarization of blood MoDCs into DC1, DC2 and DCreg cells

Human PBMCs were separated from healthy donors’ buffy coats (Etablissement Français du Sang, Rungis, France; donors were 34 to 54 old) by centrifugation over a lymphocyte separation medium (Eurobio AbCys, Courtaboeuf, France). The lack of allergen sensitization was assessed for grass, birch, ragweed pollens and house dust mites using a basophil activation test (Beckman Coulter, Villepinte, France). MoDCs were differentiated from CD14^+^ monocytes purified by magnetic cell sorting using anti‐CD14‐conjugated microbeads (MACS; Miltenyi Biotec, Bergisch Gladbach, Germany) and an autoMACS Pro Separator (Miltenyi Biotec), resulting in ≥ 95% pure CD14^+^ cells. Polarized MoDCs were generated as previously described 5, 6. Briefly, MoDCs were incubated for 24 h with either (i) highly purified lipopolysaccharide (LPS) from *Escherichia coli* (1 µg/mL; Invivogen, Toulouse, France), (ii) a mix of histamine (10 µM; Sigma–Aldrich, St. Louis, MO), IL‐25 and IL‐33 (both at 100 ng/mL; R&D Systems, Minneapolis, MN), LPS (10 ng/mL), prostaglandin E2 (PGE2, 10 µM; Sigma–Aldrich) and thymic stromal lymphopoietin (TSLP, 100 ng/mL; R&D Systems, Lille, France) or (iii) dexamethasone (1 µg/mL; Sigma–Aldrich), in order to generate DC1, DC2 and DCreg cells, respectively. MoDCs cultured for 24 h in medium only were used as controls (Ctrl‐DC). DC polarization was confirmed as follows: patterns of cytokines secreted were assessed and polarized DCs were cultured with allogeneic naive CD4^+^ T cells for 5 days to confirm the ability of these cells to support the differentiation of T_H_1, T_H_2 or Treg cells, respectively 5, 6.

### Expression profiling of microRNAs in DC1, DC2 and DCreg cells

Microarray expression analysis of 989 miRNAs was performed by Miltenyi (miRXplore microarray platform). In brief, 1.2 µg/sample of total RNA was labeled with the red fluorescent Hy5 using the miRNA/LNA labeling Exiqon kit. A pool of synthetic miRNAs in equimolar concentrations was designed by Miltenyi based on sequences of the miRBase 9.2 and were labeled with Hy3. Subsequently, the labeled material was hybridized overnight to microarrays. Fluorescence signals of the microarrays were detected using a scanner. This competitive hybridization allowed calculating the ratios between Hy5/Hy3‐labelled signals. The normalized intensities were log2‐transformed and used as a basis for further analyses.

### Quantitative analysis of miRNA expression by real‐time PCR

MiRNAs were extracted from either cell suspensions using the miRNeasy kit or from whole blood stabilized in PAXgene tubes using the PAXgene Blood miRNA kit (Qiagen Courtaboeuf, France). Complementary DNAs were synthesized using the miRScript II RT kit (Qiagen) and miRNA expression was evaluated by real‐time PCR with predesigned TaqMan microRNA assays and reagents (Applied Biosystems, Courtaboeuf, France), according to the manufacturer's instructions. Expression of the following miRNAs was assessed: miR‐132 (hsa‐miR‐132‐3p), miR‐142‐5P (hsa‐miR‐142‐5p), miR‐155 (hsa‐miR‐155‐3p), miR‐339‐5P (hsa‐miR‐339‐5p), miR‐422A (hsa‐miR‐422a), miR‐494 (hsa‐miR‐494‐3p) and miR‐744 (hsa‐miR‐744‐3p). Data were interpreted for each miRNA as copy numbers relative to endogenous RNU6B (NR‐002752) or RNU44 (NR‐002750) as references. In preliminary experiments, we demonstrated that RNU6B and RNU44 could be used indifferently. As per the manufacturer's recommendations, we used RNU44 as an internal reference for clinical samples since its abundance is higher compared to RNU6B. To calculate miRNA expression fold changes after AIT, the ΔΔ cycle threshold (Ct) method [23] was used with pre‐treatment samples as calibrators.

### Clinical samples

Blood samples from 58 allergic and 25 non‐allergic individuals were collected at the Bichat‐Claude Bernard Hospital (Paris, France) after approval of the study protocol (ref. #120147–30) by an ethical committee. Allergic patients (18 to 75 years of age) had documented symptoms of respiratory allergy, with confirmed IgE sensitization to allergens from either *Dermatophagoides pteronyssinus*, *D. farinae*, grass, birch, or ragweed pollens, cat or dog danders, cockroach or *Aspergillus fumigatus*. Positive sensitization was established based on a skin prick test wheal size ≥ 3 mm wider than the negative control (induced by vehicle only), and/or allergen‐specific serum IgE levels ≥ 0.35 kU/L measured by ImmunoCAP (Thermo Fisher Scientific, Saint Quentin en Yvelines, France). Control non‐allergic individuals were always asymptomatic to any of the aforementioned allergens, even if some (*n* = 4 out of 25) were IgE‐sensitized (i.e., with IgE titers ≥ 0.35 kU/L). Allergic rhinoconjunctivitis severity (intermittent, mild persistent, or moderate to severe persistent) was evaluated based on the ARIA classification [24]. Asthma status was defined according to GINA guidelines [25], following assessment of the lung function based on measurement of the forced expiratory volume in 1 second (FEV1%).

Details of the double‐blind, placebo‐controlled clinical trial VO56.07A (ClinicalTrials.gov NCT00619827) have been published elsewhere 12. Patients were exposed outside of the pollen season to grass pollens in a challenge chamber at baseline (Visit 2, V2), and after 2 (Visit 6, V6) and 4 (Visit 7, V7) months of treatment. They received either a grass pollen extract or placebo tablets once a day during the 4‐month study. Percentages of improvement in average rhinoconjunctivitis total symptom scores (ARTSS) were calculated between baseline and each challenge for all patients. The analysis of miRNAs was performed on PBMCs from 30 patients (*n* = 13 and 17 in the active and placebo groups, respectively) collected at Visit 3 (V3, before 1st tablet intake) and after immunotherapy (Visit 7, V7). Samples were coded and all biological analyses were conducted in a blind manner by the operators.

### Cell sorting

Pure subpopulations of either T (CD3^+^CD4^+^ and CD3^+^CD8^+^) or B lymphocytes (CD19^+^), myeloid (lin^−^ HLA‐DR^+^ CD11c^+^) or plasmacytoid (lin^−^ HLA‐DR^+^ CD123^+^) DCs, monocytes (CD14^+^) and natural killer cells (CD56^+^) were isolated from PBMCs using fluorescent‐labelled antibodies provided in the Supplementary Table 2 and a FACSAria III cell sorter (BD Biosciences) . Population purities were confirmed by post‐sorting analyses with the BD FACSDiva software (BD Biosciences, San Jose, CA) to be ≥ 95%.

### Statistical analyses

For the analysis of microarray data, ANOVA tests with repeated measurements design were applied to evaluate differences between all sample groups. The second evaluation for expression differences between one particular DC subset relative to Ctrl‐DCs was performed using the Tukey's post‐hoc test. Significant differences were considered for Tukey *P*‐value < 0.05. Differences of expression expressed as log 2 fold changes superior to 1.5 or lower than −1.5 (corresponding to fold changes superior to 2.8 or inferior to −2.8) were arbitrary considered as relevant. For other statistical analyses, differences between two groups were assessed by using the Mann–Whitney nonparametric test. To compare three groups or more, Kruskal–Wallis or Friedman tests were used for unpaired or paired data, respectively. Correlation analyses were achieved by using the nonparametric Spearman test. A *P*‐value < 0.05 was considered as significant. Statistical and graphic analyses were performed with Miltenyi's proprietary software, Prism6 (GraphPad Software, La Jolla, CA) or XLStat (Addinsoft, Paris, France) for the principal component analysis.

## Results

### Effector and regulatory DCs exhibit distinct patterns of miRNA expression

**Figure 1:**
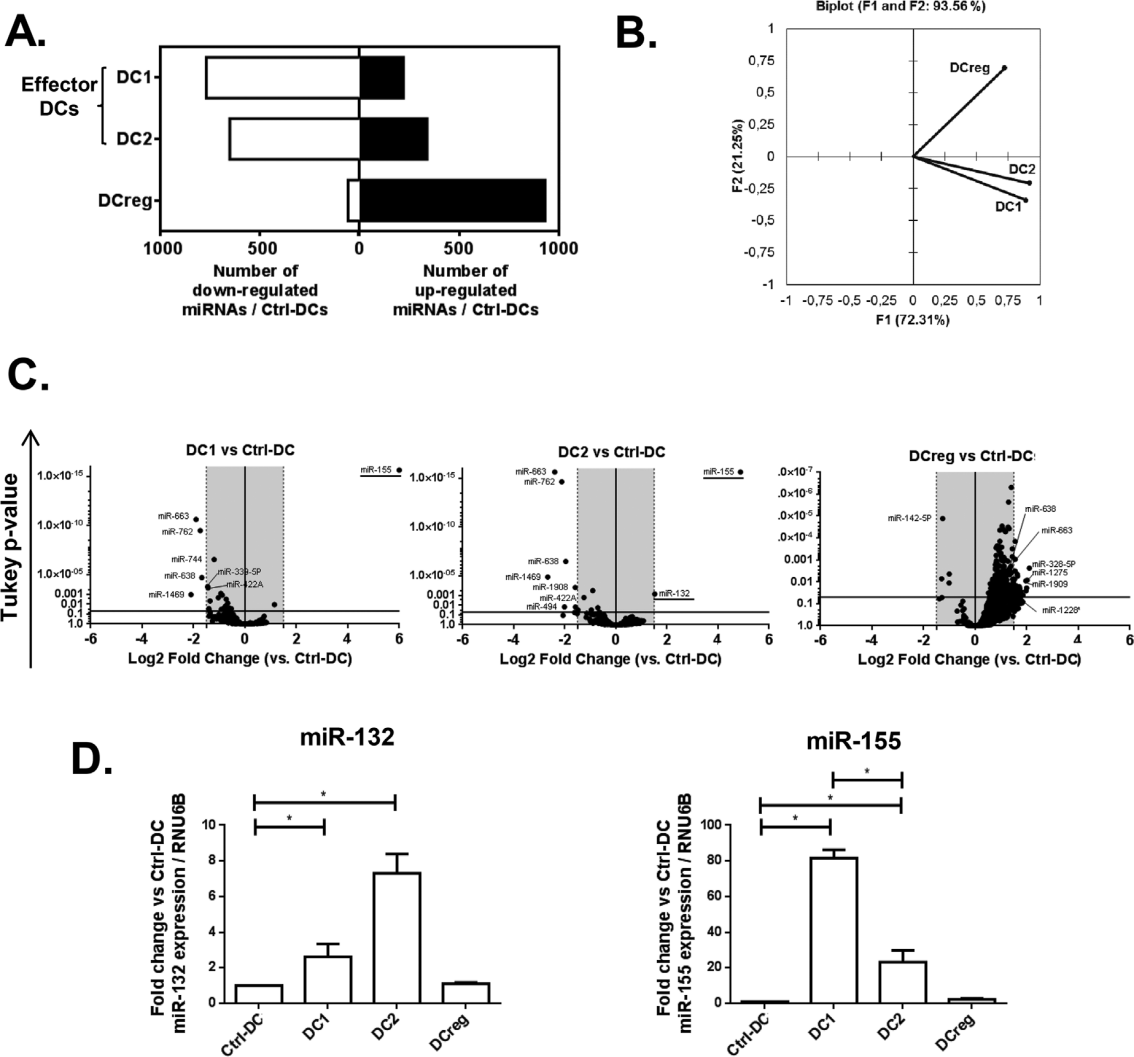
Identification of miRNAs associated with in vitro polarized MoDCs. (A) MiRNA expression was assessed by using microarrays in immature DCs, DC1, DC2, and DCreg cells from five different donors. (B) Principal component analysis (PCA) plot of the microarray data. The two first principal components (F1 and F2) are plotted. The proportion of variance explained by each component is 72.31 and 21.25%, respectively. (C) Volcano plots of miRNAs modulated in the different DC subsets. The line in ordinate indicates *P* = 0.05. The gray zone indicates miRNAs with a binary logarithm of fold change comprised between −1.5 and 1.5. (D) Confirmation by real‐time PCR of miR‐132 and miR‐155 differential expression in polarized DC subsets. Data are expressed as relative amounts of each miRNA in comparison with unstimulated DCs and are shown as mean + SEM (*n* = 5) (*P < 0.05, Friedman test).

In a first set of experiments, the expression of 989 miRNAs was evaluated by using microarrays in polarized MoDCs prepared from five different donors. DC polarization as either DC1, DC2, or DCreg cells was achieved in vitro, as described elsewhere, in presence of either (i) purified LPS; (ii) a mix of histamine, IL‐25, IL‐33, LPS, PGE2, and TSLP; or (iii) dexamethasone, respectively 5, 6. The effectiveness of this polarization was confirmed based on patterns of cytokine production and gene expression, as well as co‐cultures between DCs and naive CD4^+^ T cells (see the Methods section). Interestingly, in effector DCs (i.e., DC1 and DC2), the expression of a majority of miRNAs analyzed (77.6% for DC1s and 65.7% for DC2s) was down‐regulated when compared to unstimulated DCs (Ctrl‐DCs, Fig. 1A). In contrast, the expression of the vast majority (94.2%) of miRNAs tested was increased in DCreg cells (Fig. 1A). The principal component analysis (PCA) of microarray data confirmed that miRNA expression profiles observed in DC1s and DC2s are quite comparable, whereas both are dramatically different from the one observed in DCreg cells (Fig. 1B).

For subsequent analyses, we considered miRNA expression as being significantly modulated relative to Ctrl‐DCs when the corresponding Tukey *P*‐value was <0.05 and the binary logarithm of fold change was greater than 1.5 or smaller than −1.5 (Fig. 1C). Sixteen miRNAs reached both of these threshold values (Table 1 and Supplementary Fig. S1). To confirm these results, the expression of these miRNAs was subsequently assessed by real‐time PCR using the same samples. As a limitation that we faced in those experiments, 9 out of the 16 selected miRNAs exhibited GC‐rich sequences (i.e., containing >70% of guanine and/or cytosine creating stem‐loop structures which prevent PCR amplification, Supplementary Table S2), thus, precluding their proper quantification using this method. Overall, among the other seven remaining miRNAs, only miR‐132 and miR‐155 were confirmed by real‐time PCR to be significantly up‐regulated in DC1s and DC2s when compared to control‐DCs or DCreg cells (Fig. 1D), thereby representing potential markers of effector DCs. To confirm and extend those results, we then assessed by real‐time PCR the level of expression of these miRNAs in sorted leukocyte subsets obtained from two healthy donors, including B cells, CD4^+^, or CD8^+^ T lymphocytes, myeloid DCs (mDCs), plasmacytoid DCs (pDCs), monocytes, and NK cells. These analyses established that miR‐132 is mainly expressed by mDCs while miR‐155 expression is particularly high in pDCs (0.6% of PBMCs) and to a lesser extent in mDCs (0.8% of PBMCs) (Supplementary Fig. S2). Collectively, these data confirm that miR‐132 and miR‐155 are up‐regulated in effector DCs, and, as such, are relevant markers to assess changes occurring in blood DCs during allergic inflammation or AIT.

**Table 1:** Subject characteristics

Characteristics	Allergic rhinoconjunctivitis patients	Non‐allergic subjects
Number (*n*)	58	25
Age (years, mean ± SEM)	33.2 ± 1.7	34.2 ± 2.1
Female (*n*)	36	14
Male (*n*)	22	11
Allergic rhinoconjunctivitis severity (%)		
Intermittent (I)	34.5	0
Mild persistent (MP)	32.8	0
Moderate to severe persistent (MSP)	32.8	0
Asthma status (%)		
Asthmatic	51.7	0
IgE sensitization (%)		
*D. pteronyssinus*	53.4	16
Grass pollen	50	16
Birch pollen	34.5	0
Ragweed pollen	17.2	0

### The expression of miR‐132 and miR‐155 is decreased in the blood of patients with allergic rhinoconjunctivitis, with no detectable impact of allergen immunotherapy

**Figure 2:**
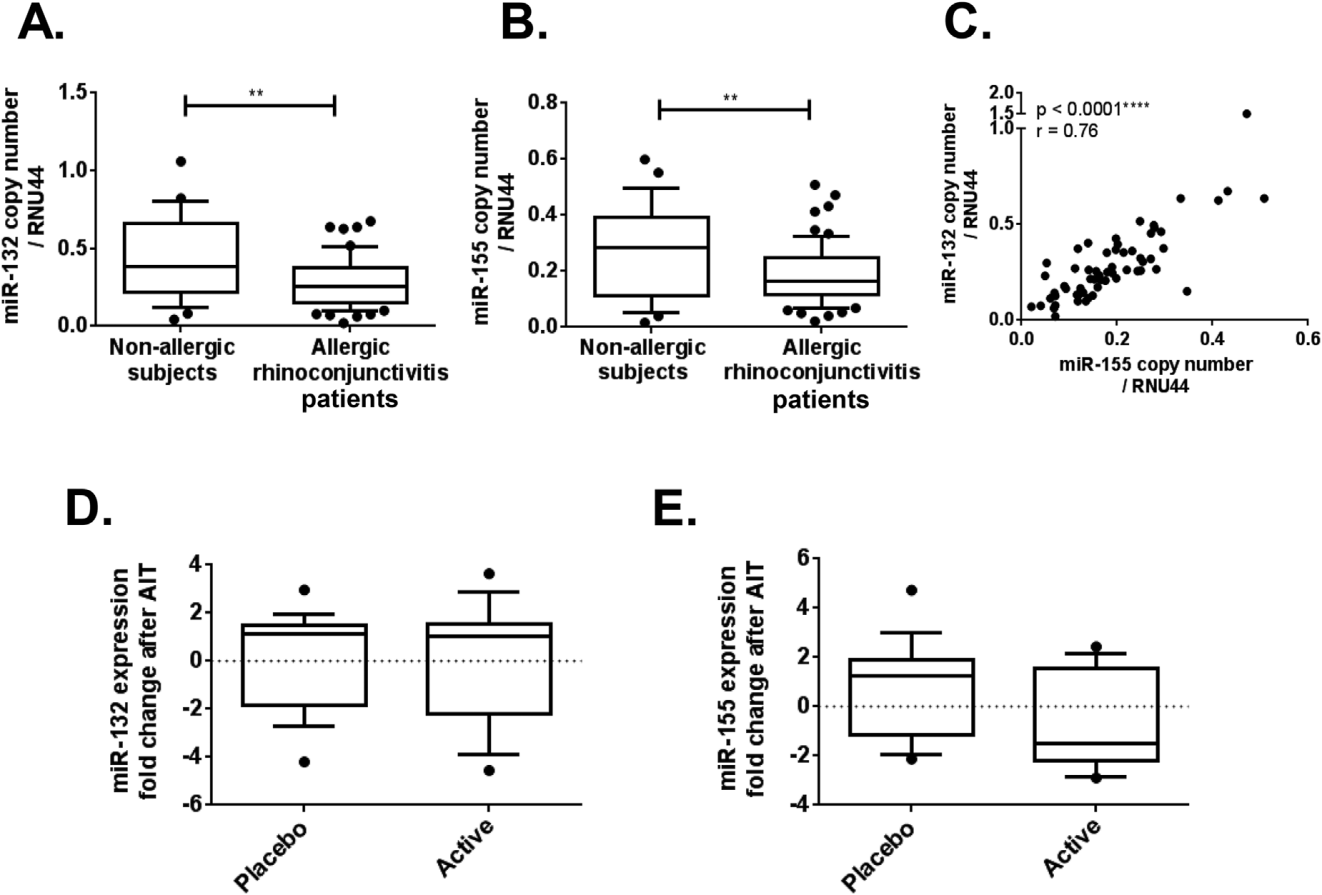
miR‐132 and miR‐155 expressions are lower in patients with allergic rhinoconjunctivitis (AR) compared to non‐allergic controls, and unaffected by AIT. (A and B) MiR‐132 and miR‐155 copy numbers were measured by real‐time PCR in blood samples from patients with allergic rhinoconjunctivitis (*n* = 58) and non‐allergic controls (*n* = 25, **P < 0.01, Mann–Whitney test). (C) Spearman correlation between miR‐132 and miR‐155 expression. (D and E) MiR‐132 or miR‐155 expression changes after four months of sublingual AIT assessed by real‐time PCR in PBMCs from patients receiving either grass pollen (*n* = 13) or placebo (*n* = 17) tablets daily.

Because it is not feasible to isolate the rare cell populations of DCs in the blood of patients enrolled in clinical studies, we next examined miRNA expression in peripheral blood. To determine whether miR‐132 and miR‐155 expressions in blood cells vary in relationship with the severity of allergic rhinoconjunctivitis, levels of miR‐132 and miR‐155 were measured in RNA samples isolated from the whole blood of 58 adult patients with allergic rhinoconjunctivitis and 25 non‐allergic subjects used as controls, as described in Table 2. Surprisingly, expression levels of miR‐132 and miR‐155 were significantly lower in blood samples from patients with allergic rhinoconjunctivitis, when compared to non‐allergic subjects (*P* < 0.01, Fig. 2A and B). Interestingly, the expression of these two miRNAs was strongly correlated (*P* < 0.0001) in the blood of each individual patient (Fig. 2C). Nonetheless, the expression of miR‐132 and ‐155 could not be related to rhinoconjunctivitis severity (Supplementary Fig. S3A and B), nor to lung function impairment (Supplementary Fig. S3C and D).

**Table 2:** Overview of miRNA expression in polarized DC subsets

	DC1		DC2		DCreg	
miR‐	Down‐regulated	Up‐regulated	Down‐regulated	Up‐regulated	Down‐regulated	Up‐regulated
132		X*		X		
155		X		X		
142‐5P					X	
328‐5P						X
339‐5P	X					
422A	X		X			
494			X			
638	X		X			X
663	X		X			X
744	X					
762	X		X			X
1275						X
1228*						X
1469	X		X			
1908			X			
1909						X

*As assessed by real‐time PCR.

Given those previous results, we subsequently assessed the expression of miR‐132 and miR‐155 in blood samples obtained from patients with grass pollen‐induced rhinoconjunctivitis having received sublingual AIT for four months, in the context of a double blind, placebo‐controlled study performed in a challenge chamber 12. Out of the 89 patients originally included in this trial, material was only available to monitor the expression of miR‐132 and miR‐155 in PBMCs from 13 active patients and 17 placebo subjects. In these samples, miR‐132 and miR‐155 expression levels were not dramatically modified after AIT (Fig. 2D and E). Importantly, miR‐132 and miR‐155 expression fold changes over the treatment period were not correlated with clinical benefit at an individual patient level (Supplementary Fig. S4A and B).

## Discussion

MiRNAs contribute to the regulation of various physiological and pathological immune responses, including allergic inflammation 8. For example, miR‐19a promotes IL‐13 production by human CD4^+^ T cells 9, whereas the miR‐17∼92 cluster contributes to the development of B cell tolerance 13. In monocytes, macrophages and DCs, miRNAs are also involved in the establishment of the recently described “innate immune memory” 14. Based on our recent observations that (i) polarized DCs encompassing regulatory (tolerogenic) and effector (pro‐inflammatory) cells bear specific molecular signatures; and (ii) AIT alters such DC polarization markers in the blood 5, 6, we proceeded herein to identify miRNAs specifically modulated in DC1, DC2, or DCreg cells and assessed their expression in relationship with allergic rhinoconjunctivitis severity or AIT efficacy.

Comparative microarray analyses established that patterns of miRNA expression are similar between effector DCs (DC1s and DC2s), but dramatically different from the one observed in DCreg cells. Specifically, we identified 16 miRNAs differentially regulated between DC1, DC2, and DCreg cells but failed to identify any single miRNA discriminating DC1s from DC2s. We confirmed that both miR‐132 and miR‐155 are expressed in blood DCs, and further, that they are up‐regulated in effector subsets (i.e., DC1s and DC2s). The miR‐132 is known to be involved in the development and functional regulation of neurons 15, 16 while miR‐155 enhances the pro‐inflammatory activity of T cells or DCs in diseases such as rheumatoid arthritis 17 or allergies 10, 16, 18. Of note, both miR‐132 and miR‐155 have been linked by others to the induction of either T_H_1 or T_H_2 responses. For example, miR‐155 supports the production of IFN‐γ by CD4^+^ T cells 19, whereas, miR‐155‐deficient mice spontaneously develop lung inflammation with high numbers of IL‐4 and IL‐5‐secreting T_H_2 cells 20. The role of miR‐132 in allergic inflammation has been less documented so far, even if a recent report reveals that miR‐132 expression is increased in bronchial brushing samples following allergen exposure and possibly contributes to epithelial wall damage 21. Nevertheless, similarly to miR‐155, miR‐132 is also induced following TLR4 engagement, thus, promoting a T_H_1‐type of inflammation 22. Together, these data suggest that miR‐155 expression might reflect a type 1 immune response, whereas miR‐132 can contribute to either type 1 or 2 immune responses, in line with our observation that this latter miRNA is expressed by DC1s as well as DC2s. In this context, we observed that expression levels of both miR‐132 and miR‐155 are jointly reduced in PBMCs of patients with allergic rhinoconjunctivitis, when compared with healthy individuals. We hypothesize that the latter reflects a potential down regulation of DC1s expressing miR‐132 and miR‐155, as a consequence of overwhelming T_H_2 responses to the allergens in such allergic patients. However, considering the paucity of blood DCs, we cannot exclude that a reduced number of T_H_1 cells also contributes to the decreased expression of these miRNAs observed in blood samples from these patients with allergic rhinoconjunctivitis.

MiRNAs have been proposed as a source of potential biomarkers to predict and monitor therapy outcomes 12, even if as of today, none of them has been yet proven to be relevant to document AIT efficacy. Herein, no changes in miR‐132 or miR‐155 expression were observed in the blood of grass pollen allergic patients receiving AIT. Although the short course of treatment (four months) and/or the low frequency of blood DCs could explain our failure to detect any modulation of miR‐132/‐155 expression in total blood samples, it should be noted that, under those very same conditions, we were able to document molecular changes reflecting the balance between DCreg cells and DC2s in the patients’ blood 5, 6. Consequently, in our opinion, these negative results raise several limitations to the use of miRNAs as candidate biomarkers of disease severity or treatment efficacy. First, miRNAs are expressed by multiple cell types and, as a result, miRNA signatures are unlikely to be specific enough to reflect a complex and heterogeneous condition such as allergic inflammation. In addition, miRNAs in our hands only exhibit a limited dynamic range (∼ twofold) of variation in their level of expression when comparing various subsets of polarized DCs. Also, as a technical limitation to set up miRNA‐based biomarker assays, potentially interesting miRNAs identified in our initial microarray study could not be confirmed by real‐time PCR because of their GC‐rich sequence. Noteworthy, an analysis of miRNAs in pulmonary or nasal fluids, might be highly relevant to identify local immunological changes linked to allergic rhinitis or asthma. Altogether, we successfully identified miRNAs differentially expressed in effector and regulatory DCs. Two of those miRNAs associated with effector DCs (i.e., DC1 and DC2 cells) are differentially expressed between patients with allergic rhinoconjunctivitis and non‐allergic individuals. Our results suggest that the use of such miRNAs as markers of AIT efficacy remains as of today very challenging.

## Supporting information

Additional supporting information may be found in the online version of this article at the publisher's website.

Supporting Data

**Figure S1**. Heatmaps of microarray data. Heatmaps representing miRNAs significantly modulated in DC1 (A), DC2 (B) and DCreg cells (C) compared to unstimulated DCs (Ctrl‐DC).
**Figure S2**. Analysis of miR‐132 and miR‐155 expressions in leukocyte subsets. Monocytes (CD14^+^), T (CD3^+^ CD4^+^ and CD3^+^ CD8^+^) or B lymphocytes (CD19^+^), natural killer cells (NK, CD56^+^), mDCs (lin^−^ HLA‐DR^+^ CD11c^+^) or pDCs (lin^−^ HLA‐DR^+^ CD123^+^) were sorted from PBMCs of two healthy donors by flow cytometry. The expression of miRNA‐132 and miR‐155 was measured in each subset by real‐time PCR. One representative experiment out of two is shown.
**Figure S3**. MiR‐132 and miR‐155 copy numbers in the blood of allergic rhinoconjunctivitis patients depending on allergic rhinitis severity and respiratory function. (A and B) MiR‐132 and miR‐155 copy numbers in blood samples from allergic patients with intermittent (I, *n* = 22), mild persistent (MP, *n* = 21) and moderate to severe persistent (MSP, *n* = 15) symptoms. (C and D) Spearman correlations between Forced Expiratory Volume in 1 sec (FEV1%) values and miR‐132 or miR‐155 copy numbers.
**Figure S4**. Correlation between clinical score improvement and miR‐132 or miR‐155 expression. (A and B) Spearman correlations between miR‐132 or miR‐155 expression changes after four months of sublingual AIT and percentages of improvement of the clinical score in patients from the active or placebo group.
**Table S1**. Antibodies used for cell sorting.
**Table S2**. Sequences of miRNAs differentially expressed between DC1, DC2 and DCreg cells.

**DC1.** Microarray results DC1 vs Unstimulated DCs

**DC2.** Microarray results DC2 vs Unstimulated DCs

**DCreg.** Microarray results DCreg vs Unstimulated DCs
