# Supplementary material for: Effector and regulatory dendritic cells display distinct patterns of miRNA expression
Source: Immun Inflamm Dis. 2017 May 12;5(3):310–7. doi: 10.1002/iid3.165 (PMC5569363; doi:10.1002/iid3.165)
Supplement: Supplementary file 1 — Supporting Data [file IID3-5-310-s001.docx]

**Supplementary Figure legends**

**Supplementary Figure 1: Heatmaps of microarray data.**

Heatmaps representing miRNAs significantly modulated in DC1s (**A**), DC2s (**B**) and DCreg cells (**C**) compared to unstimulated DCs (Ctrl-DC).

**Supplementary Figure 2: Analysis of miR-132 and miR-155 expressions in leukocyte subsets.**

Monocytes (CD14^+^), T (CD3^+^ CD4^+^ and CD3^+^ CD8^+^) or B lymphocytes (CD19^+^), natural killer cells (NK, CD56^+^), mDCs (lin^−^ HLA-DR^+^ CD11c^+^) or pDCs (lin^−^ HLA-DR^+^ CD123^+^) were sorted from PBMCs of two healthy donors by flow cytometry. The expression of miRNA-132 and miR-155 was measured in each subset by real-time PCR. One representative experiment out of 2 is shown.

**Supplementary Figure 3: MiR-132 and miR-155 copy numbers in the blood of allergic rhinoconjunctivitis patients depending on allergic rhinitis severity and respiratory function.**

**(A and C)** MiR-132 and miR-155 copy numbers in blood samples from allergic patients with intermittent (I, n=22), mild persistent (MP, n=21) and moderate to severe persistent (MSP, n=15) symptoms. **(B and D)**. Spearman correlations between Forced Expiratory Volume in 1 second (FEV1%) values and miR-132 or miR-155 copy numbers.

**Supplementary Figure 4: Correlation between clinical score improvement and miR-132 or miR-155 expression.**

(**A and B**) Spearman correlations between miR-132 or miR-155 expression changes after 4 months of sublingual AIT and percentages of improvement of the clinical score in patients from the active or placebo group.

**Supplementary Materials and Methods**

**Polarization of blood monocyte-derived DCs (MoDCs) into DC1, DC2 and DCreg cells**

Human PBMCs were separated from healthy donors’ buffy coats (Etablissement Français du Sang, Rungis, France; donors were 34 to 54 old) by centrifugation over a lymphocyte separation medium (Eurobio AbCys, Courtaboeuf, France). The lack of allergen sensitization was assessed for grass, birch, ragweed pollens and house dust mites using a basophil activation test (Beckman Coulter, Villepinte, France). MoDCs were differentiated from CD14^+^ monocytes purified by magnetic cell sorting using anti-CD14-conjugated microbeads (MACS; Miltenyi Biotec, Bergisch Gladbach, Germany) and an autoMACS Pro Separator (Miltenyi Biotec), resulting in ≥ 95% pure CD14^+^ cells. Polarized MoDCs were generated as previously described [[5](#_ENREF_5), [6](#_ENREF_6)]. Briefly, MoDCs were incubated for 24 hours with either (i) highly purified lipopolysaccharide (LPS) from *Escherichia coli* (1 µg/mL; Invivogen, Toulouse, France), (ii) a mix of histamine (10 µM; Sigma-Aldrich, Saint-Louis, MO), IL-25 and IL-33 (both at 100 ng/mL; R&D Systems, Minneapolis, MN), LPS (10 ng/mL), prostaglandin E2 (PGE2, 10 µM; Sigma-Aldrich) and thymic stromal lymphopoietin (TSLP, 100 ng/mL; R&D Systems, Lille, France) or (iii) dexamethasone (1 µg/mL; Sigma-Aldrich), in order to generate DC1, DC2 and DCreg cells, respectively. MoDCs cultured for 24 hours in medium only were used as controls (Ctrl-DC). DC polarization was confirmed as follows: patterns of cytokines secreted were assessed and polarized DCs were cultured with allogeneic naive CD4^+^ T cells for 5 days to confirm the ability of these cells to support the differentiation of T_H_1, T_H_2 or Treg cells, respectively [[5](#_ENREF_5), [6](#_ENREF_6)].

**Expression profiling of microRNAs in DC1, DC2 and DCreg cells**

Microarray expression analysis of 989 miRNAs was performed by Miltenyi (miRXplore microarray platform). In brief, 1.2 µg/sample of total RNA was labeled with the red fluorescent Hy5 using the miRNA/LNA labeling Exiqon kit. A pool of synthetic miRNAs in equimolar concentrations was designed by Miltenyi based on sequences of the miRBase 9.2 and were labeled with Hy3. Subsequently, the labeled material was hybridized overnight to microarrays. Fluorescence signals of the microarrays were detected using a scanner. This competitive hybridization allowed calculating the ratios between Hy5/Hy3-labelled signals. The normalized intensities were log2-transformed and used as a basis for further analyses.

**Quantitative analysis of miRNA expression by real-time PCR**

MiRNAs were extracted from either cell suspensions using the miRNeasy kit or from whole blood stabilized in PAXgene tubes using the PAXgene Blood miRNA kit (Qiagen Courtaboeuf, France). Complementary DNAs were synthesized using the miRScript II RT kit (Qiagen) and miRNA expression was evaluated by real-time PCR with predesigned TaqMan microRNA assays and reagents (Applied Biosystems, Courtaboeuf, France), according to the manufacturer’s instructions. Expression of the following miRNAs was assessed: miR-132 (hsa-miR-132-3p), miR-142-5P (hsa-miR-142-5p), miR-155 (hsa-miR-155-3p), miR-339-5P (hsa-miR-339-5p), miR-422A (hsa-miR-422a), miR-494 (hsa-miR-494-3p) and miR-744 (hsa-miR-744-3p). Data were interpreted for each miRNA as copy numbers relative to endogenous RNU6B (NR-002752) or RNU44 (NR-002750) as references. In preliminary experiments, we demonstrated that RNU6B and RNU44 could be used indifferently. As per the manufacturer’s recommendations, we used RNU44 as an internal reference for clinical samples since its abundance is higher compared to RNU6B. To calculate miRNA expression fold changes after AIT, the ΔΔ cycle threshold (Ct) method [[23](#_ENREF_23)] was used with pre-treatment samples as calibrators.

**Clinical samples**

Blood samples from 58 allergic and 25 non-allergic individuals were collected at the Bichat-Claude Bernard Hospital (Paris, France) after approval of the study protocol (ref. #120147-30) by an ethical committee. Allergic patients (18 to 75 years of age) had documented symptoms of respiratory allergy, with confirmed IgE sensitization to allergens from either *Dermatophagoides pteronyssinus*, *D. farinae*, grass, birch, or ragweed pollens, cat or dog danders, cockroach or *Aspergillus fumigatus*. Positive sensitization was established based on a skin prick test wheal size ≥ 3 mm wider than the negative control (induced by vehicle only), and / or allergen-specific serum IgE levels ≥ 0.35 kU/L measured by ImmunoCAP (Thermo Fisher Scientific, Saint Quentin en Yvelines, France). Control non-allergic individuals were always asymptomatic to any of the aforementioned allergens, even if some (n=4 out of 25) were IgE-sensitized (*i.e.* with IgE titers ≥ 0.35 kU/L). Allergic rhinoconjunctivitis severity (intermittent, mild persistent, or moderate to severe persistent) was evaluated based on the ARIA classification [[24](#_ENREF_24)]. Asthma status was defined according to GINA guidelines [[25](#_ENREF_25)], following assessment of the lung function based on measurement of the forced expiratory volume in 1 second (FEV1%).

Details of the double-blind, placebo-controlled clinical trial VO56.07A (ClinicalTrials.gov NCT00619827) have been published elsewhere [[12](#_ENREF_12)]. Patients were exposed outside of the pollen season to grass pollens in a challenge chamber at baseline (Visit 2, V2), and after 2 (Visit 6, V6) and 4 (Visit 7, V7) months of treatment. They received either a grass pollen extract or placebo tablets once a day during the 4-month study. Percentages of improvement in average rhinoconjunctivitis total symptom scores (ARTSS) were calculated between baseline and each challenge for all patients. The analysis of miRNAs was performed on PBMCs from 30 patients (n = 13 and 17 in the active and placebo groups, respectively) collected at Visit 3 (V3, before 1^st^ tablet intake) and after immunotherapy (Visit 7, V7). Samples were coded and all biological analyses were conducted in a blind manner by the operators.

**Cell sorting**

Pure subpopulations of either T (CD3^+^CD4^+^ and CD3^+^CD8^+^) or B lymphocytes (CD19^+^), myeloid (lin^-^ HLA-DR^+^ CD11c^+^) or plasmacytoid (lin^-^ HLA-DR^+^ CD123^+^) DCs, monocytes (CD14^+^) and natural killer cells (CD56^+^) were isolated from PBMCs using fluorescent-labelled antibodies provided in the **Supplementary Table 2** and a FACSAria III cell sorter (BD Biosciences) . Population purities were confirmed by post-sorting analyses with the BD FACSDiva software (BD Biosciences, San Jose, CA) to be ≥ 95%.

**Statistical analyses**

For the analysis of microarray data, ANOVA tests with repeated measurements design were applied to evaluate differences between all sample groups. The second evaluation for expression differences between one particular DC subset relative to Ctrl-DCs was performed using the Tukey’s post-hoc test. Significant differences were considered for Tukey p-value < 0.05. Differences of expression expressed as log 2 fold changes superior to 1.5 or lower than -1.5 (corresponding to fold changes superior to 2.8 or inferior to -2.8) were arbitrary considered as relevant. For other statistical analyses, differences between two groups were assessed by using the Mann-Whitney nonparametric test. To compare three groups or more, Kruskal-Wallis or Friedman tests were used for unpaired or paired data, respectively. Correlation analyses were achieved by using the nonparametric Spearman test. A p-value < 0.05 was considered as significant. Statistical and graphic analyses were performed with Miltenyi´s proprietary software, Prism6 (GraphPad Software, La Jolla, CA) or XLStat (Addinsoft, Paris, France) for the principal component analysis.

**Supplementary References**

1. Gueguen C, Bouley J, Moussu H, Luce S, Duchateau M, Chamot-Rooke J, Pallardy M, Lombardi V, Nony E, Baron-Bodo V, Mascarell L, Moingeon P. Changes in markers associated with dendritic cells driving the differentiation of either T2 cells or regulatory T cells correlate with clinical benefit during allergen immunotherapy. J Allergy Clin Immunol 2016 Feb;137(2):545-58.

2. Zimmer A, Bouley J, Le Mignon M, Pliquet E, Horiot S, Turfkruyer M, Baron-Bodo V, Horak F, Nony E, Louise A, Moussu H, Mascarell L, Moingeon P. A regulatory dendritic cell signature correlates with the clinical efficacy of allergen-specific sublingual immunotherapy. J Allergy Clin Immunol 2012; 129:1020-1030.

3. Schmittgen TD, Livak KJ. Analyzing real-time PCR data by the comparative C(T) method. Nat Protoc 2008; 3:1101-1108.

4. Bousquet J, et al. Development and implementation of guidelines in allergic rhinitis - an ARIA-GA2LEN paper. Allergy 2010; 65:1212-1221.

5. Bateman ED, Hurd SS, Barnes PJ, Bousquet J, Drazen JM, FitzGerald M, Gibson P, Ohta K, O'Byrne P, Pedersen SE, Pizzichini E, Sullivan SD, Wenzel SE, Zar HJ. Global strategy for asthma management and prevention: GINA executive summary. Eur Respir J 2008; 31:143-178.

6. Horak F, Zieglmayer P, Zieglmayer R, Lemell P, Devillier P, Montagut A, Melac M, Galvain S, Jean-Alphonse S, Van Overtvelt L, Moingeon P, Le Gall M. Early onset of action of a 5-grass-pollen 300-IR sublingual immunotherapy tablet evaluated in an allergen challenge chamber. J Allergy Clin Immunol 2009; 124:471-477.
