# Supplementary material for: Effector and regulatory dendritic cells display distinct patterns of miRNA expression
Source: Immun Inflamm Dis. 2017 May 12;5(3):310–7. doi: 10.1002/iid3.165 (PMC5569363; doi:10.1002/iid3.165)
Supplement: Supplementary file 2 — Figure S1. Heatmaps of microarray data. Heatmaps representing miRNAs significantly modulated in DC1 (A), DC2 (B) and DCreg cells (C) compared to unstimulated DCs (Ctrl‐DC). Figure S2. Analysis of miR‐132 and miR‐155 expressions in leukocyte subsets. Monocytes (CD14+), T (CD3+ CD4+ and CD3+ CD8+) or B lymphocytes (CD19+), natural killer cells (NK, CD56+), mDCs (lin− HLA‐DR+ CD11c+) or pDCs (lin− HLA‐DR+ CD123+) were sorted from PBMCs of two healthy donors by flow cytometry. The expression of miRNA‐132 and miR‐155 was measured in each subset by real‐time PCR. One representative experiment out of two is shown. Figure S3. MiR‐132 and miR‐155 copy numbers in the blood of allergic rhinoconjunctivitis patients depending on allergic rhinitis severity and respiratory function. (A and B) MiR‐132 and miR‐155 copy numbers in blood samples from allergic patients with intermittent (I, n = 22), mild persistent (MP, n = 21) and moderate to severe persistent (MSP, n = 15) symptoms. (C and D) Spearman correlations between Forced Expiratory Volume in 1 sec (FEV1%) values and miR‐132 or miR‐155 copy numbers. Figure S4. Correlation between clinical score improvement and miR‐132 or miR‐155 expression. (A and B) Spearman correlations between miR‐132 or miR‐155 expression changes after four months of sublingual AIT and percentages of improvement of the clinical score in patients from the active or placebo group. Table S1. Antibodies used for cell sorting. Table S2. Sequences of miRNAs differentially expressed between DC1, DC2 and DCreg cells. [file IID3-5-310-s002.pptx]

## Slide 1
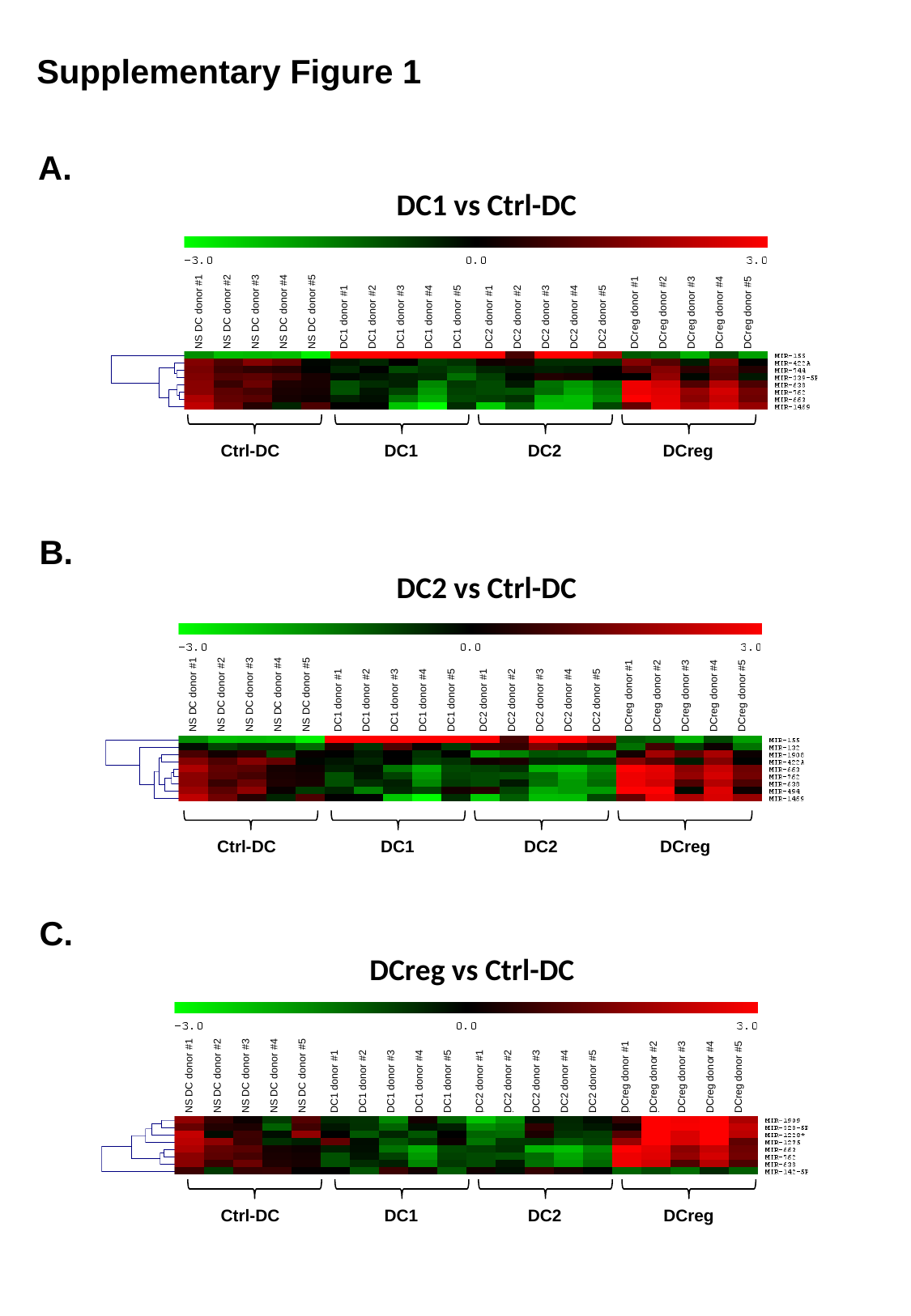

Supplementary Figure 1
A.
DC1 vs Ctrl-DC
DC1 donor #1
DC1 donor #2
DC1 donor #3
DC1 donor #4
DC1 donor #5
DC2 donor #1
DC2 donor #2
DC2 donor #3
DC2 donor #4
DC2 donor #5
DCreg donor #1
DCreg donor #2
DCreg donor #3
DCreg donor #4
DCreg donor #5
NS DC donor #1
NS DC donor #2
NS DC donor #3
NS DC donor #4
NS DC donor #5
Ctrl-DC
DC1
DC2
DCreg
B.
DC2 vs Ctrl-DC
DC1 donor #1
DC1 donor #2
DC1 donor #3
DC1 donor #4
DC1 donor #5
DC2 donor #1
DC2 donor #2
DC2 donor #3
DC2 donor #4
DC2 donor #5
DCreg donor #1
DCreg donor #2
DCreg donor #3
DCreg donor #4
DCreg donor #5
NS DC donor #1
NS DC donor #2
NS DC donor #3
NS DC donor #4
NS DC donor #5
Ctrl-DC
DC1
DC2
DCreg
C.
DCreg vs Ctrl-DC
DC1 donor #1
DC1 donor #2
DC1 donor #3
DC1 donor #4
DC1 donor #5
DC2 donor #1
DC2 donor #2
DC2 donor #3
DC2 donor #4
DC2 donor #5
DCreg donor #1
DCreg donor #2
DCreg donor #3
DCreg donor #4
DCreg donor #5
NS DC donor #1
NS DC donor #2
NS DC donor #3
NS DC donor #4
NS DC donor #5
Ctrl-DC
DC1
DC2
DCreg

## Slide 2
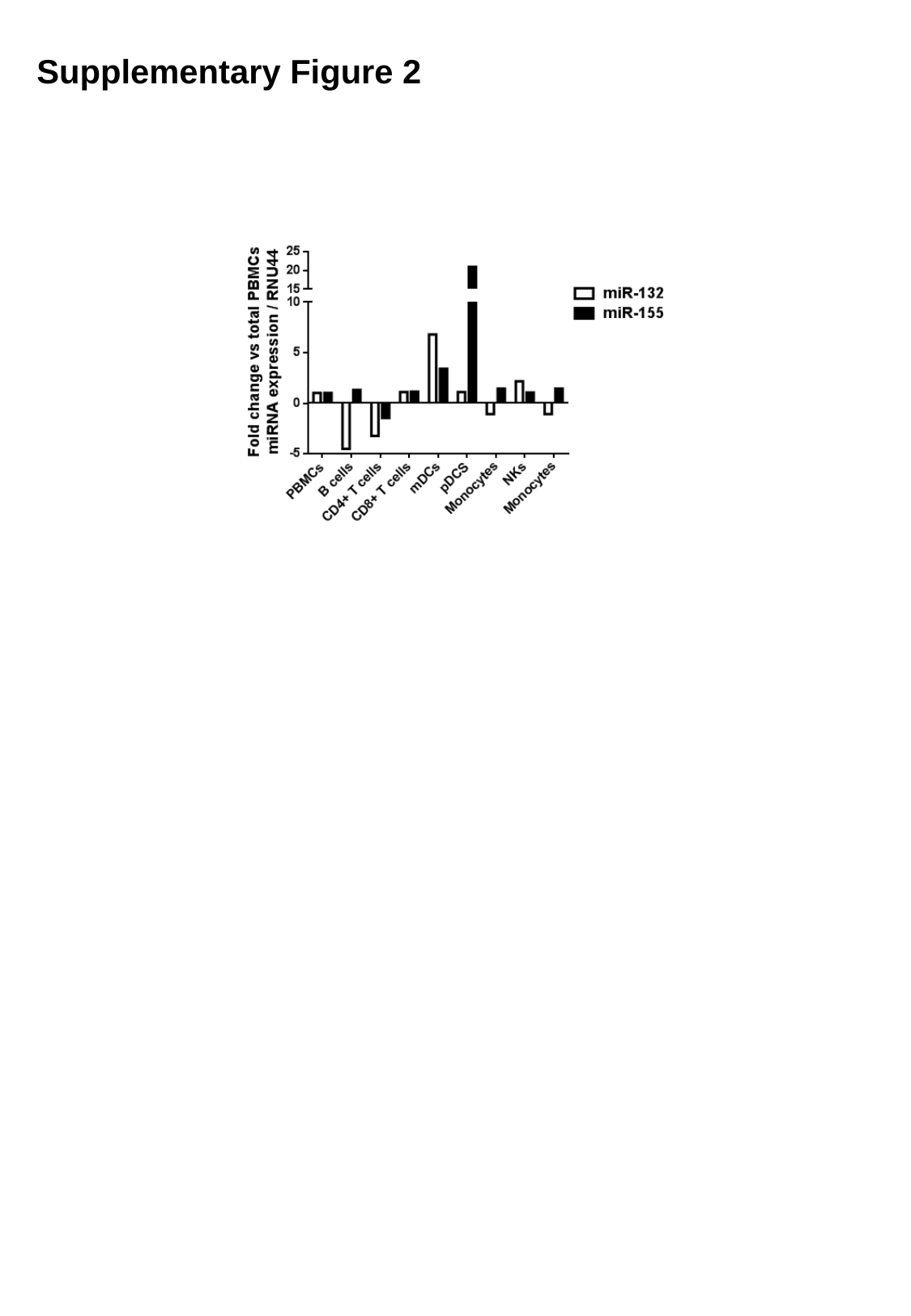

Supplementary Figure 2

## Slide 3
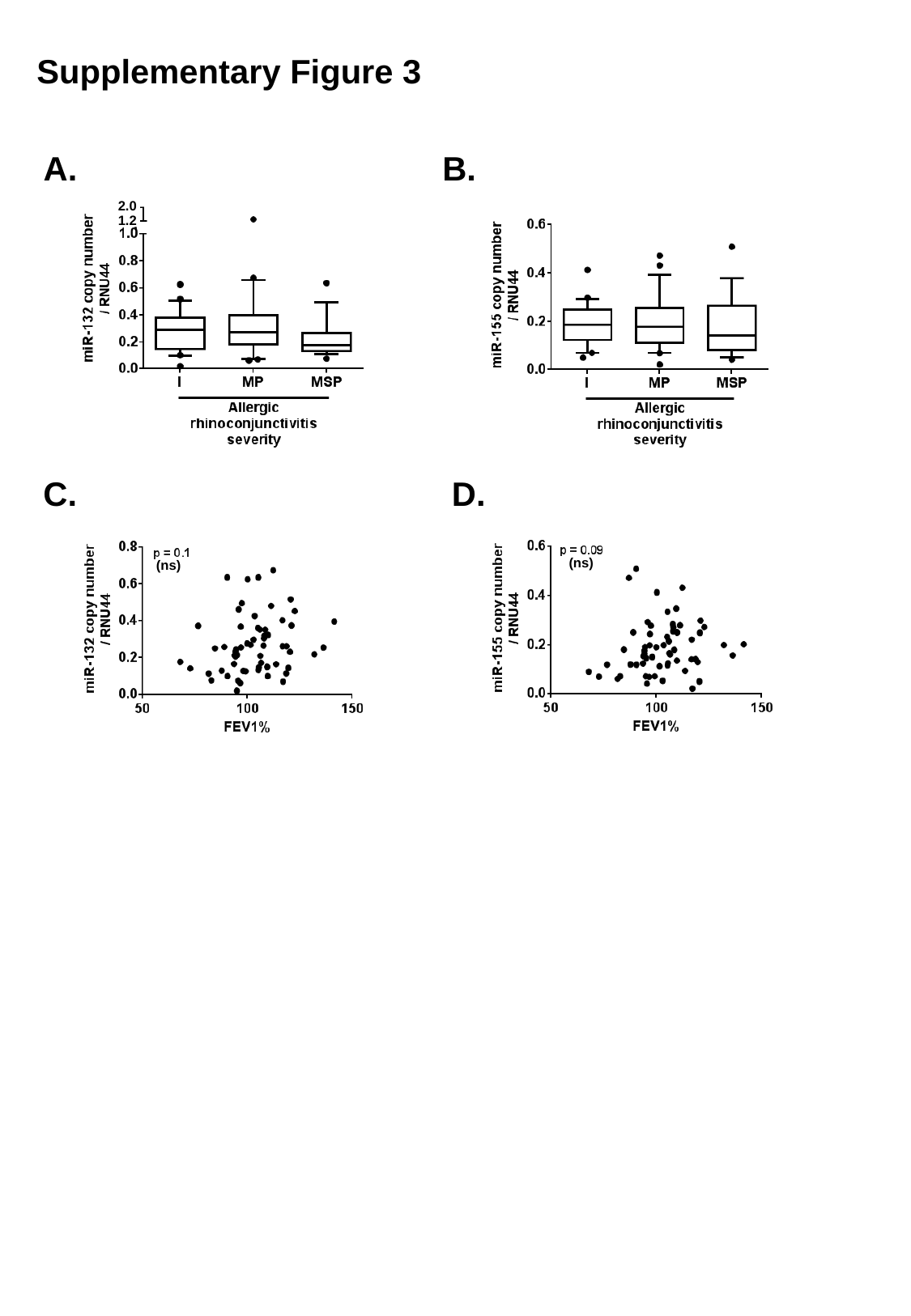

Supplementary Figure 3
A.
B.
2.0
1.2
C.
D.
(ns)
(ns)

## Slide 4
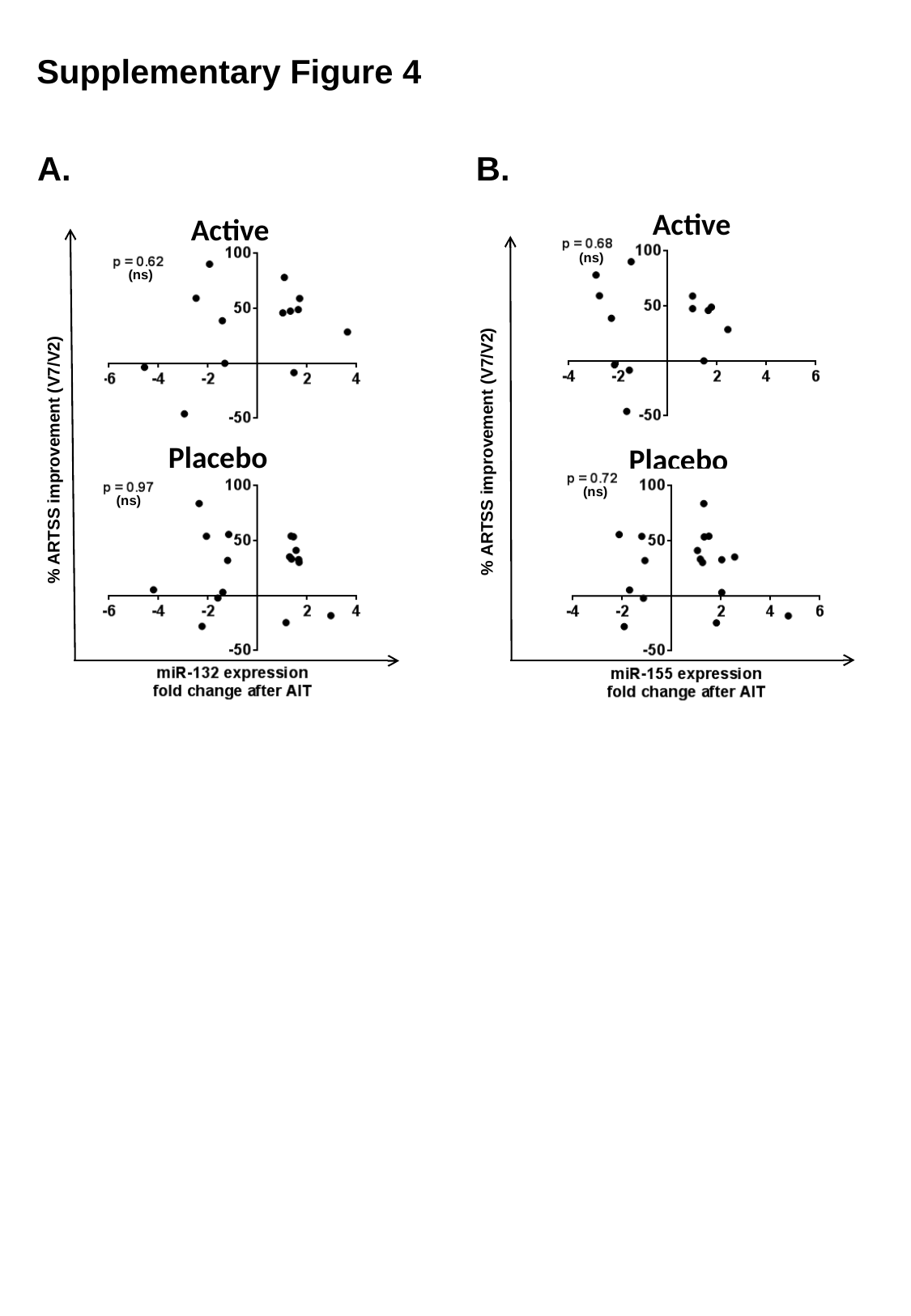

Supplementary Figure 4
A.
B.
Active
Active
% ARTSS improvement (V7/V2)
% ARTSS improvement (V7/V2)
Placebo
Placebo
(ns)
(ns)
(ns)
(ns)

## Slide 5
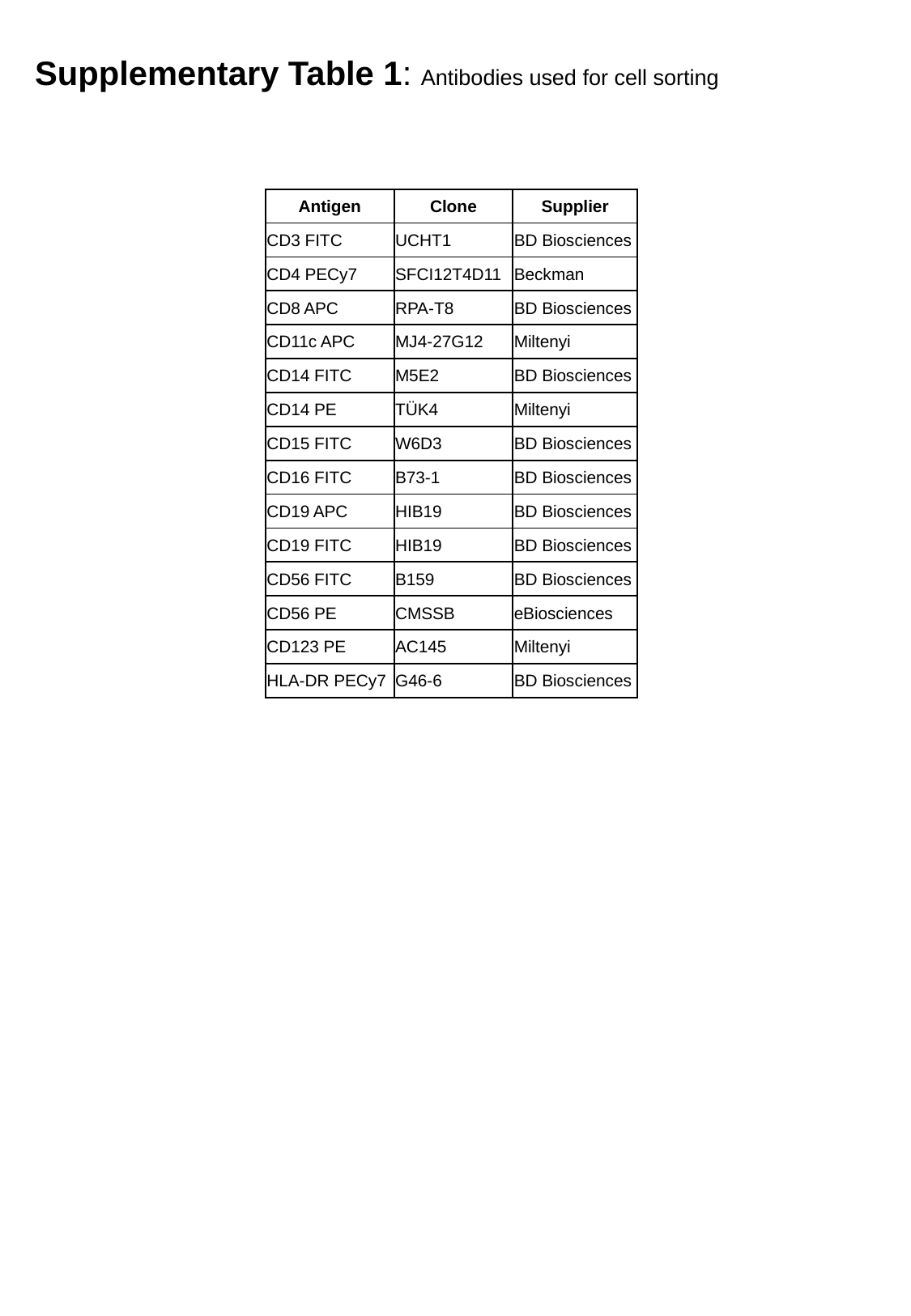

Supplementary Table 1: Antibodies used for cell sorting
| Antigen | Clone | Supplier |
| --- | --- | --- |
| CD3 FITC | UCHT1 | BD Biosciences |
| CD4 PECy7 | SFCI12T4D11 | Beckman |
| CD8 APC | RPA-T8 | BD Biosciences |
| CD11c APC | MJ4-27G12 | Miltenyi |
| CD14 FITC | M5E2 | BD Biosciences |
| CD14 PE | TÜK4 | Miltenyi |
| CD15 FITC | W6D3 | BD Biosciences |
| CD16 FITC | B73-1 | BD Biosciences |
| CD19 APC | HIB19 | BD Biosciences |
| CD19 FITC | HIB19 | BD Biosciences |
| CD56 FITC | B159 | BD Biosciences |
| CD56 PE | CMSSB | eBiosciences |
| CD123 PE | AC145 | Miltenyi |
| HLA-DR PECy7 | G46-6 | BD Biosciences |

## Slide 6
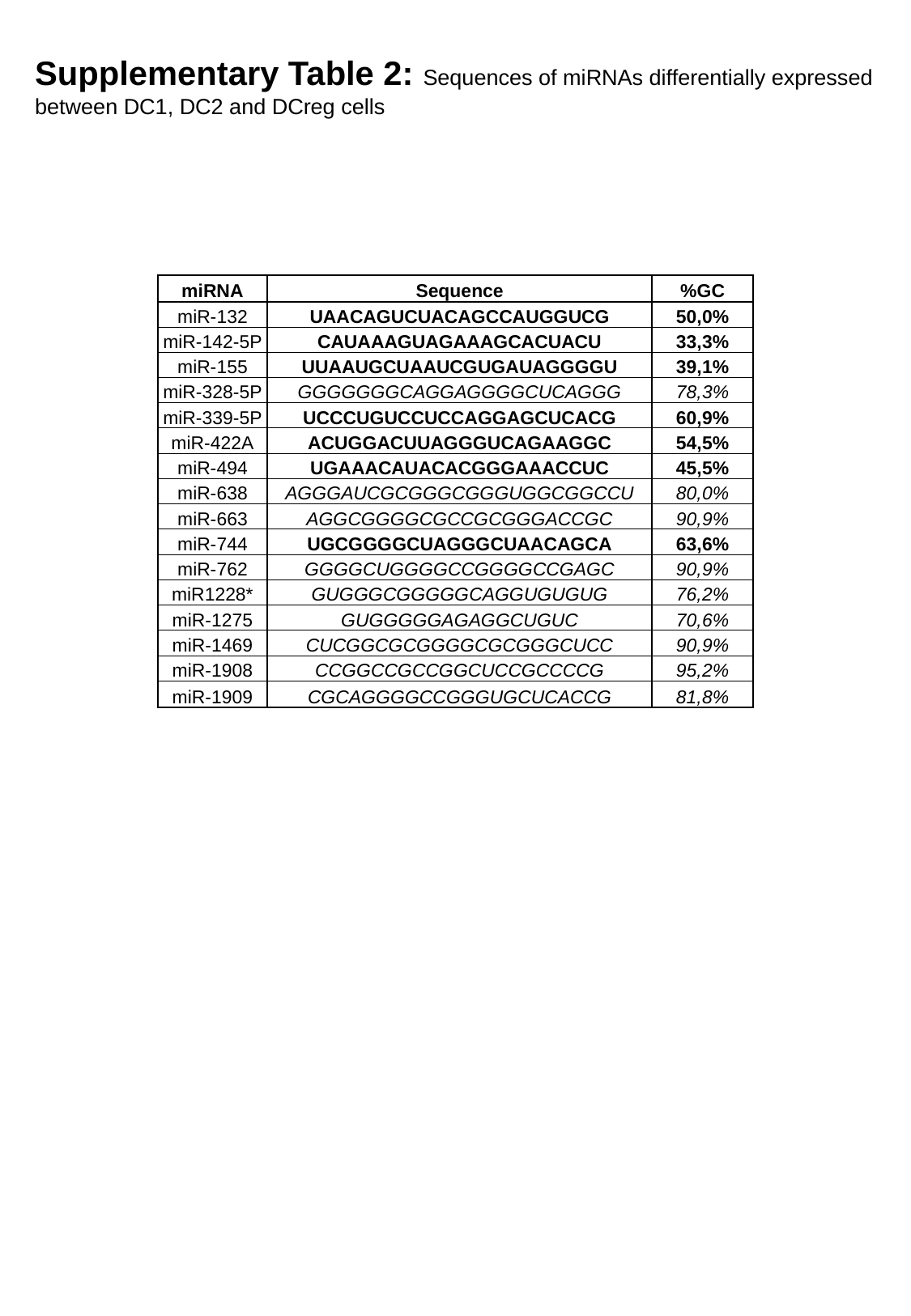

Supplementary Table 2: Sequences of miRNAs differentially expressed between DC1, DC2 and DCreg cells
| miRNA | Sequence | %GC |
| --- | --- | --- |
| miR-132 | UAACAGUCUACAGCCAUGGUCG | 50,0% |
| miR-142-5P | CAUAAAGUAGAAAGCACUACU | 33,3% |
| miR-155 | UUAAUGCUAAUCGUGAUAGGGGU | 39,1% |
| miR-328-5P | GGGGGGGCAGGAGGGGCUCAGGG | 78,3% |
| miR-339-5P | UCCCUGUCCUCCAGGAGCUCACG | 60,9% |
| miR-422A | ACUGGACUUAGGGUCAGAAGGC | 54,5% |
| miR-494 | UGAAACAUACACGGGAAACCUC | 45,5% |
| miR-638 | AGGGAUCGCGGGCGGGUGGCGGCCU | 80,0% |
| miR-663 | AGGCGGGGCGCCGCGGGACCGC | 90,9% |
| miR-744 | UGCGGGGCUAGGGCUAACAGCA | 63,6% |
| miR-762 | GGGGCUGGGGCCGGGGCCGAGC | 90,9% |
| miR1228\* | GUGGGCGGGGGCAGGUGUGUG | 76,2% |
| miR-1275 | GUGGGGGAGAGGCUGUC | 70,6% |
| miR-1469 | CUCGGCGCGGGGCGCGGGCUCC | 90,9% |
| miR-1908 | CCGGCCGCCGGCUCCGCCCCG | 95,2% |
| miR-1909 | CGCAGGGGCCGGGUGCUCACCG | 81,8% |

## Slide 7
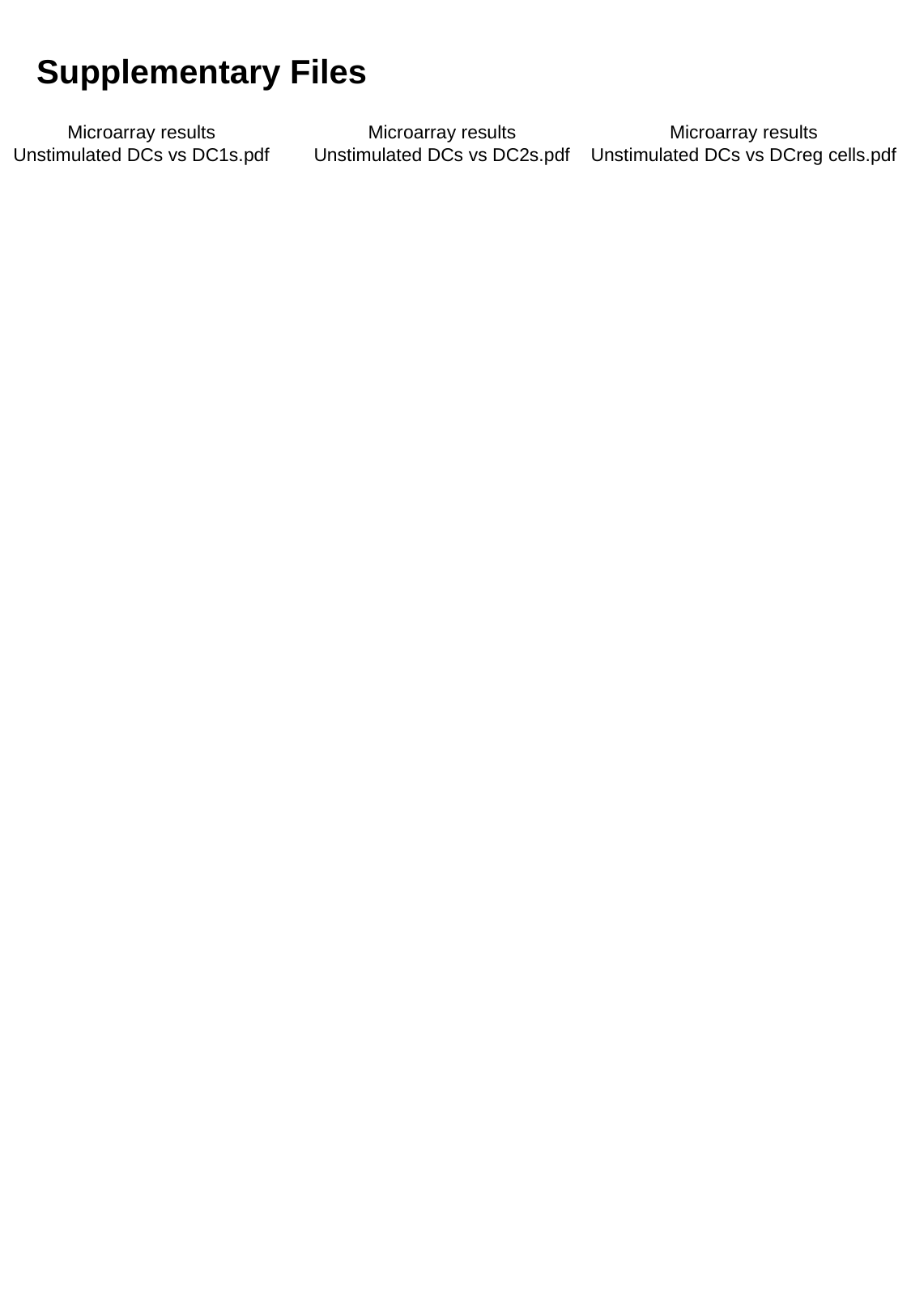

Supplementary Files
Microarray results
Unstimulated DCs vs DC1s.pdf
Microarray results
Unstimulated DCs vs DC2s.pdf
Microarray results
Unstimulated DCs vs DCreg cells.pdf
