# Supplementary material for: Effector and regulatory dendritic cells display distinct patterns of miRNA expression
Source: Immun Inflamm Dis. 2017 May 12;5(3):310–7. doi: 10.1002/iid3.165 (PMC5569363; doi:10.1002/iid3.165)
Supplement: Supplementary file 3 — DC1. Microarray results DC1 vs Unstimulated DCs [file IID3-5-310-s003.pdf]

	DC1 vs Immature DC		
**miRNA name**	**Tukey.p-value**	**Fold_Change**	**Log2_Ratio**
MIR-155	0,00E+00	81,876	6,355
MIR-663	2,31E-11	-3,737	-1,902
MIR-762	3,14E-10	-3,338	-1,739
MIR-744	2,82E-07	-2,290	-1,196
MIR-638	1,92E-05	-3,193	-1,675
MIR-339-5P	1,60E-04	-2,730	-1,449
MIR-422A	2,14E-04	-2,696	-1,431
MIR-423-3P	7,86E-04	-1,920	-0,941
MIR-1469	1,05E-03	-4,285	-2,099
MIR-487B	1,19E-03	-1,847	-0,885
MIR-378*	2,12E-03	-2,050	-1,036
MIR-30E*	3,08E-03	-1,657	-0,728
MIR-148B	4,58E-03	-2,562	-1,357
MIR-342-3P	7,65E-03	-1,591	-0,670
MIR-222	1,07E-02	2,215	1,147
MIR-425	1,49E-02	-1,692	-0,758
MIR-29C	2,19E-02	-1,743	-0,801
MIR-223	2,37E-02	-1,520	-0,604
MIR-15B	2,49E-02	-1,525	-0,608
MIR-421-3P	2,78E-02	-1,445	-0,531
MIR-142-3P	2,83E-02	-2,638	-1,400
MIR-371-5P	3,23E-02	-1,892	-0,920
MIR-1228*	4,22E-02	-2,634	-1,397
MIR-484	4,50E-02	-1,622	-0,698
MIR-380-5P	4,87E-02	-1,599	-0,677
MIR-326	5,12E-02	-1,626	-0,702
MIR-378	5,13E-02	-1,618	-0,694
MIR-1537	5,38E-02	-1,578	-0,659
MIR-502-3P	5,48E-02	-1,478	-0,564
MIR-662	5,50E-02	-1,506	-0,591
MIR-27B	5,64E-02	-1,703	-0,768
MIR-500A*	6,73E-02	-1,468	-0,554
MIR-512-5P	6,92E-02	-1,602	-0,680
MIR-1260	7,06E-02	-2,550	-1,350
HCMV-MIR-US33-5P	7,30E-02	-1,492	-0,577
MIR-636	7,47E-02	-1,626	-0,701
MIR-596	7,66E-02	-1,517	-0,601
EBV-MIR-BART10	7,84E-02	-1,480	-0,565
MIR-615-3P	9,42E-02	-1,490	-0,576
MIR-611	9,83E-02	-1,535	-0,618
EBV-MIR-BART3-3P	9,85E-02	-1,500	-0,585
MIR-138	1,02E-01	-1,617	-0,693
MIR-362-5P	1,06E-01	-1,543	-0,625
MIR-612	1,08E-01	-1,537	-0,620
MIR-1290	1,08E-01	-1,373	-0,457
MIR-532-3P	1,08E-01	-1,435	-0,521
MIR-190	1,08E-01	-1,760	-0,815
MIR-554	1,09E-01	-2,024	-1,017
MIR-373*	1,09E-01	-1,443	-0,529
MIR-671-5P	1,10E-01	-1,634	-0,708
MIR-630	1,10E-01	-1,480	-0,566
MIR-1208	1,14E-01	-1,394	-0,479
MIR-412	1,25E-01	-1,484	-0,569
HCMV-MIR-UL112	1,26E-01	-1,456	-0,542
MIR-19A	1,26E-01	-1,814	-0,860
MIR-665	1,28E-01	-1,672	-0,742
MIR-720	1,32E-01	-1,518	-0,602

MIR-370	1,37E-01	-1,465	-0,551
MIR-1270	1,39E-01	-1,519	-0,603
HCMV-MIR-US5-1	1,42E-01	-1,506	-0,590
MIR-490-3P	1,43E-01	-1,693	-0,759
MIR-323-5P	1,48E-01	-1,456	-0,542
MIR-661	1,48E-01	-1,651	-0,723
MIR-518A-5P-527_2	1,51E-01	-1,527	-0,611
MIR-518F*	1,51E-01	-1,479	-0,565
MIR-597	1,51E-01	-1,646	-0,719
MIR-100	1,52E-01	-1,589	-0,668
EBV-MIR-BART18-3P	1,53E-01	-1,485	-0,571
MIR-487A	1,57E-01	-1,403	-0,488
MIR-494	1,58E-01	-2,637	-1,399
MIR-595	1,60E-01	-1,473	-0,558
MIR-361-5P	1,61E-01	-1,427	-0,513
MIR-675	1,62E-01	-1,503	-0,588
MIR-503	1,65E-01	-1,715	-0,778
EBV-MIR-BART13	1,65E-01	-1,555	-0,637
MIR-20A	1,66E-01	1,676	0,745
MIR-30C-1*	1,67E-01	-1,558	-0,640
MIR-518D-5P-520C-5P-526A	1,68E-01	-1,432	-0,518
MIR-490-5P	1,70E-01	-1,553	-0,635
MIR-1909	1,76E-01	-2,417	-1,273
MIR-518E	1,79E-01	-1,443	-0,529
LET-7C	1,79E-01	-1,337	-0,419
MIR-654-5P	1,85E-01	-1,441	-0,527
MIR-1827	1,86E-01	-2,241	-1,164
MIR-501-5P	1,86E-01	-1,342	-0,424
MIR-615-5P	1,86E-01	-1,459	-0,545
MIR-593*	1,87E-01	-1,607	-0,685
MIR-1321	1,88E-01	-1,424	-0,510
MIR-550A	1,88E-01	-1,424	-0,510
HCMV-MIR-UL70-5P	1,95E-01	-1,729	-0,790
MIR-124	1,98E-01	-1,657	-0,729
HIV1-MIR-H1	1,98E-01	-1,491	-0,576
EBV-MIR-BART16	1,99E-01	-1,493	-0,578
MIR-518E*-519A*-1-519B-5P-519C-5P-522*-523*-526C	2,00E-01	-1,356	-0,440
KSHV-MIR-K12-3*	2,01E-01	-1,470	-0,556
MIR-1253	2,01E-01	-1,875	-0,907
MIR-140-3P	2,01E-01	-1,390	-0,475
MIR-135A*	2,01E-01	-1,435	-0,522
MIR-328-3P	2,03E-01	-1,415	-0,501
MIR-668	2,04E-01	-1,410	-0,496
MIR-140-5P	2,04E-01	-1,562	-0,643
MIR-128	2,08E-01	-1,430	-0,516
HCMV-MIR-US4	2,10E-01	-1,408	-0,494
MIR-934	2,13E-01	-1,313	-0,393
MIR-30E	2,18E-01	-1,490	-0,575
MIR-193B	2,18E-01	-1,423	-0,508
MIR-637	2,22E-01	-1,491	-0,577
MIR-1246	2,26E-01	-2,039	-1,028
MIR-548J	2,27E-01	-1,540	-0,623
MIR-568	2,27E-01	-1,597	-0,675
MIR-30C	2,28E-01	-1,353	-0,436
MIR-885-5P	2,36E-01	-1,404	-0,489
MIR-519C-5P	2,36E-01	-1,392	-0,477
MIR-509-5P	2,38E-01	-1,468	-0,554
MIR-600	2,39E-01	-1,416	-0,502
EBV-MIR-BART11-5P	2,39E-01	-1,368	-0,452

KSHV-MIR-K12-10B	2,40E-01	-1,315	-0,395
MIR-125B	2,41E-01	-1,636	-0,710
EBV-MIR-BHRF1-1	2,42E-01	-1,331	-0,413
MIR-614	2,44E-01	-1,740	-0,799
MIR-648	2,44E-01	-1,667	-0,737
MIR-142-5P	2,45E-01	-1,404	-0,490
MIR-877	2,46E-01	-1,598	-0,676
MIR-384-3P	2,48E-01	-1,480	-0,565
MIR-23A	2,48E-01	-1,462	-0,548
MIR-132	2,50E-01	1,635	0,709
MIR-524-3P	2,53E-01	-1,428	-0,514
MIR-718	2,54E-01	-1,378	-0,463
MIR-1910	2,54E-01	-2,079	-1,056
MIR-492	2,57E-01	-1,423	-0,509
MIR-621	2,58E-01	-1,669	-0,739
MIR-517*	2,59E-01	-1,371	-0,455
MIR-107	2,65E-01	-1,249	-0,321
HCMV-MIR-US25-2-3P	2,66E-01	-1,479	-0,564
MIR-1301	2,67E-01	-1,506	-0,590
MIR-1197	2,67E-01	-1,496	-0,581
HCMV-MIR-US25-2-5P	2,69E-01	-1,431	-0,517
MIR-433-3P	2,71E-01	-1,380	-0,465
MIR-523	2,72E-01	-1,433	-0,519
KSHV-MIR-K12-12*	2,73E-01	-1,376	-0,460
MIR-566	2,74E-01	-1,522	-0,606
MIR-608	2,75E-01	-1,406	-0,492
MIR-185	2,76E-01	-1,495	-0,580
MIR-1267	2,77E-01	-1,546	-0,629
MIR-629	2,82E-01	-1,371	-0,455
MIR-130B	2,82E-01	-1,297	-0,376
MIR-520D-5P	2,83E-01	-1,381	-0,465
KSHV-MIR-K12-5	2,83E-01	-1,419	-0,504
MIR-640	2,86E-01	-1,401	-0,487
MIR-631	2,86E-01	-1,514	-0,598
MIR-935	2,87E-01	-1,279	-0,355
MIR-658	2,88E-01	-1,529	-0,613
MIR-432*	2,90E-01	-1,549	-0,632
MIR-150*	2,93E-01	-2,000	-1,000
MIR-331-5P	2,96E-01	-1,371	-0,455
MIR-639	2,99E-01	-1,679	-0,748
MIR-136*	3,00E-01	-1,455	-0,541
MIR-602	3,02E-01	-1,516	-0,600
MIR-106B	3,05E-01	-1,525	-0,609
MIR-518D-3P	3,06E-01	-1,372	-0,456
MIR-532-5P	3,06E-01	-1,610	-0,687
MIR-483-5P	3,07E-01	-1,588	-0,667
MIR-33B	3,08E-01	-2,144	-1,100
MIR-874	3,09E-01	-1,515	-0,599
MIR-93	3,11E-01	-1,402	-0,488
EBV-MIR-BART20-3P	3,11E-01	-1,499	-0,584
MIR-646	3,16E-01	-1,505	-0,590
MIR-590-3P	3,16E-01	-1,603	-0,681
EBV-MIR-BART6-5P	3,17E-01	-1,508	-0,593
KSHV-MIR-K12-1	3,18E-01	-1,375	-0,460
MIR-1305	3,19E-01	-1,519	-0,603
MIR-324-3P	3,21E-01	-1,374	-0,459
MIR-339-3P	3,21E-01	-1,374	-0,458
MIR-885-3P	3,23E-01	-1,366	-0,450
MIR-548H	3,23E-01	-1,409	-0,495

SV40-MIR-S1-5P	3,23E-01	-1,435	-0,521
MIR-498	3,24E-01	-1,376	-0,460
EBV-MIR-BART12	3,24E-01	-1,389	-0,475
MIR-770-3P	3,26E-01	-1,432	-0,518
MIR-512-3P	3,26E-01	-1,352	-0,435
MIR-887	3,29E-01	-1,461	-0,547
MIR-769-3P	3,30E-01	-1,422	-0,508
MIR-31	3,35E-01	-1,385	-0,470
MIR-708	3,35E-01	-1,466	-0,552
MIR-767-5P	3,35E-01	-1,432	-0,518
MIR-30D*	3,36E-01	-1,651	-0,724
MIR-1225-5P	3,36E-01	-1,455	-0,541
MIR-200B	3,37E-01	-1,315	-0,395
HCMV-MIR-US5-2	3,38E-01	-1,352	-0,435
MIR-324-5P	3,43E-01	-1,381	-0,465
MIR-1914	3,43E-01	-1,787	-0,838
MIR-516A-5P	3,45E-01	-1,389	-0,474
EBV-MIR-BART17-3P	3,45E-01	-1,325	-0,406
MIR-330-5P	3,46E-01	-1,304	-0,383
HSV1-MIR-H1	3,47E-01	-1,426	-0,512
MIR-605	3,48E-01	-1,439	-0,525
MIR-1252	3,48E-01	-1,337	-0,419
MIR-323B-5P	3,49E-01	-1,320	-0,400
MIR-296-3P	3,50E-01	-1,420	-0,505
MIR-601	3,52E-01	-1,334	-0,416
MIR-556-3P	3,56E-01	-1,400	-0,485
MIR-320B	3,57E-01	-1,360	-0,444
MIR-26A-2*	3,61E-01	-1,502	-0,587
KSHV-MIR-K12-2	3,62E-01	-1,374	-0,458
MIR-431	3,64E-01	-1,400	-0,486
MIR-199A-5P	3,64E-01	-1,386	-0,470
MIR-99B	3,64E-01	-1,366	-0,450
HIV1-MIR-N367	3,65E-01	-1,412	-0,498
MIR-96*	3,67E-01	1,551	0,633
MIR-103A	3,68E-01	-1,247	-0,319
MIR-892B	3,68E-01	-1,359	-0,442
MIR-320A	3,69E-01	-1,352	-0,435
MIR-647	3,72E-01	-1,489	-0,574
MIR-1909*	3,72E-01	-1,782	-0,834
EBV-MIR-BHRF1-3	3,75E-01	-1,357	-0,440
MIR-936	3,77E-01	-1,261	-0,335
MIR-491-5P	3,77E-01	-1,495	-0,580
MIR-933	3,81E-01	-1,350	-0,433
MIR-122	3,84E-01	-1,277	-0,353
MIR-210	3,84E-01	-1,604	-0,682
MIR-541-5P	3,85E-01	-1,445	-0,532
MIR-765	3,87E-01	-1,556	-0,638
MIR-873	3,87E-01	-1,436	-0,522
MIR-942	3,88E-01	-1,352	-0,435
MIR-27A	3,88E-01	-1,305	-0,384
MIR-660	3,89E-01	-1,458	-0,544
MIR-519E*	3,90E-01	-1,369	-0,453
MIR-769-5P	3,91E-01	-1,412	-0,498
MIR-134	3,91E-01	-1,314	-0,394
MIR-298	3,93E-01	-1,362	-0,446
EBV-MIR-BART7	3,94E-01	-1,334	-0,416
MIR-1294	3,96E-01	-1,308	-0,388
MIR-188-5P	4,00E-01	-1,426	-0,512
MIR-489	4,02E-01	-1,335	-0,417

MIR-542-5P	4,02E-01	-1,353	-0,436
MIR-758	4,03E-01	-1,453	-0,539
HCMV-MIR-UL70-3P	4,05E-01	-1,351	-0,434
MIR-322-MIR-424	4,07E-01	-1,928	-0,947
MIR-214	4,07E-01	-1,343	-0,425
MIR-593	4,12E-01	-1,388	-0,473
MIR-345-5P	4,18E-01	-1,339	-0,421
MIR-575	4,22E-01	-1,377	-0,461
MIR-30A*	4,23E-01	-1,358	-0,442
MIR-151-5P-151B	4,23E-01	-1,321	-0,402
KSHV-MIR-K12-7	4,23E-01	-1,354	-0,437
EBV-MIR-BART17-5P	4,26E-01	-1,351	-0,434
HCMV-MIR-US25-1	4,27E-01	-1,382	-0,467
MIR-372	4,27E-01	-1,331	-0,413
KSHV-MIR-K12-8	4,27E-01	-1,448	-0,534
MIR-129-5P	4,31E-01	-1,341	-0,423
MIR-149*	4,32E-01	-1,622	-0,698
MIR-515-3P	4,34E-01	-1,352	-0,435
EBV-MIR-BART6-3P	4,37E-01	-1,415	-0,500
MIR-99A*	4,37E-01	-1,507	-0,592
HIV1-MIR-TAR-5P	4,37E-01	-1,271	-0,346
MIR-198	4,38E-01	-1,385	-0,470
MIR-564	4,39E-01	-1,367	-0,451
MIR-1538	4,48E-01	-1,524	-0,608
MIR-588	4,49E-01	-1,379	-0,464
MIR-497	4,49E-01	-1,353	-0,437
MIR-518C*	4,50E-01	-1,257	-0,329
MIR-1468	4,53E-01	-1,250	-0,322
LET-7A	4,55E-01	1,459	0,545
MIR-205	4,57E-01	-1,455	-0,541
MIR-770-5P	4,58E-01	-1,451	-0,538
MIR-510	4,61E-01	-1,359	-0,443
MIR-548E	4,63E-01	-1,271	-0,346
MIR-149	4,63E-01	-1,333	-0,414
MIR-525-5P	4,63E-01	-1,266	-0,340
MIR-143	4,64E-01	-1,353	-0,437
MIR-146B-5P	4,64E-01	-1,275	-0,351
MIR-139-5P	4,64E-01	-1,372	-0,456
MIR-1273	4,65E-01	-1,409	-0,494
MIR-99A	4,69E-01	-1,524	-0,608
MIR-296-5P	4,72E-01	-1,366	-0,450
MIR-18B	4,74E-01	-1,341	-0,423
MIR-516B	4,76E-01	-1,278	-0,353
MIR-130A*	4,76E-01	-1,464	-0,550
MIR-643	4,80E-01	-1,286	-0,362
MIR-761	4,81E-01	-1,371	-0,455
MIR-411*	4,83E-01	-1,303	-0,382
MIR-187	4,86E-01	-1,264	-0,338
MIR-483-3P	4,87E-01	-1,280	-0,356
MIR-34C-5P	4,87E-01	-1,473	-0,559
MIR-1183	4,88E-01	-1,380	-0,465
MIR-105	4,88E-01	-1,343	-0,426
MIR-183	4,88E-01	-1,365	-0,449
MIR-183*	4,89E-01	-1,403	-0,489
HCMV-MIR-US33-3P	4,89E-01	-1,367	-0,451
MIR-574-5P	4,91E-01	-1,432	-0,518
MIR-363*	4,92E-01	-1,318	-0,399
MIR-567	4,94E-01	-1,489	-0,575
MIR-328-5P	4,95E-01	-1,790	-0,840

MIR-625	4,97E-01	-1,284	-0,361
MIR-23B	4,98E-01	-1,273	-0,348
MIR-1297	5,02E-01	-1,257	-0,330
MIR-623	5,03E-01	-1,303	-0,382
MIR-92A-2*	5,03E-01	-1,393	-0,478
MIR-939	5,04E-01	-1,302	-0,381
MIR-543-3P	5,05E-01	-1,272	-0,347
MIR-485-5P	5,06E-01	-1,298	-0,376
MIR-619	5,08E-01	-1,470	-0,556
MIR-25*	5,08E-01	-1,242	-0,313
MIR-202-5P	5,10E-01	-1,288	-0,366
MIR-1973	5,11E-01	-2,400	-1,263
MIR-1207-5P	5,12E-01	-1,385	-0,470
MIR-875-3P	5,13E-01	-1,334	-0,416
KSHV-MIR-K12-4-3P	5,16E-01	-1,312	-0,392
MIR-382	5,31E-01	-1,215	-0,280
KSHV-MIR-K12-3	5,33E-01	-1,367	-0,451
MIR-635	5,34E-01	-1,419	-0,505
HCMV-MIR-UL148D	5,35E-01	-1,297	-0,375
MIR-587	5,35E-01	-1,340	-0,423
MIR-671-3P	5,40E-01	-1,263	-0,337
MIR-373	5,40E-01	-1,326	-0,407
MIR-19B	5,41E-01	-1,483	-0,569
MIR-711	5,44E-01	-1,315	-0,395
HCMV-MIR-UL36	5,45E-01	-1,242	-0,313
MIR-518A-3P	5,46E-01	-1,300	-0,379
MIR-628-3P	5,51E-01	-1,214	-0,280
MIR-22	5,51E-01	-1,414	-0,499
MIR-320C	5,52E-01	-1,321	-0,401
MIR-1262	5,54E-01	-1,258	-0,332
MIR-509-3P	5,57E-01	-1,278	-0,354
EBV-MIR-BART1-5P	5,60E-01	-1,225	-0,293
MIR-589*	5,61E-01	-1,345	-0,428
MIR-502-5P	5,62E-01	-1,249	-0,320
MIR-555	5,62E-01	-1,268	-0,343
MIR-450B-3P	5,64E-01	-1,342	-0,425
MIR-573	5,65E-01	-1,346	-0,428
MIR-1257	5,65E-01	-1,203	-0,267
MIR-432	5,67E-01	-1,251	-0,323
MIR-550A*	5,67E-01	-1,259	-0,333
MIR-622	5,67E-01	-1,288	-0,365
MIR-571	5,69E-01	-1,276	-0,352
MIR-184	5,69E-01	-1,286	-0,362
MIR-1275	5,73E-01	-1,765	-0,820
MIR-486-5P	5,75E-01	-1,238	-0,308
MIR-645	5,76E-01	-1,315	-0,395
EBV-MIR-BART11-3P	5,77E-01	-1,294	-0,372
MIR-485-3P	5,78E-01	-1,210	-0,275
MIR-181C	5,78E-01	-1,305	-0,384
MIR-518B	5,79E-01	-1,291	-0,369
MIR-302C*	5,80E-01	-1,240	-0,310
MIR-508-5P	5,81E-01	-1,375	-0,460
MIR-125A-3P	5,81E-01	-1,551	-0,633
EBV-MIR-BART14-3P	5,81E-01	-1,262	-0,335
HSV1-MIR-H4	5,83E-01	-1,213	-0,278
KSHV-MIR-K12-6-5P	5,90E-01	-1,232	-0,301
MIR-302F	5,90E-01	-1,252	-0,324
MIR-548I	5,94E-01	-1,408	-0,493
MIR-449B	5,94E-01	-1,252	-0,325

MIR-337-5P	5,95E-01	-1,306	-0,385
MIR-572	5,96E-01	-1,273	-0,349
MIR-766	5,96E-01	-1,360	-0,444
MIR-500A-500B	5,98E-01	-1,288	-0,366
MIR-20B	6,01E-01	-1,545	-0,628
MIR-381	6,04E-01	-1,276	-0,351
MIR-19B-1*	6,04E-01	-1,275	-0,351
MIR-556-5P	6,07E-01	-1,394	-0,479
MIR-409-5P	6,08E-01	-1,296	-0,374
MIR-2110	6,08E-01	-1,350	-0,433
MIR-323-3P	6,10E-01	-1,247	-0,319
MIR-2052	6,11E-01	-1,231	-0,299
MIR-193B*	6,12E-01	-1,356	-0,439
MIR-181D	6,12E-01	-1,239	-0,309
MIR-937	6,14E-01	-1,279	-0,355
MIR-1224-5P	6,17E-01	-1,279	-0,354
MIR-383	6,18E-01	-1,209	-0,274
MIR-197	6,19E-01	-1,389	-0,474
KSHV-MIR-K12-11	6,22E-01	-1,283	-0,360
EBV-MIR-BART8-3P	6,25E-01	-1,312	-0,392
MIR-379	6,26E-01	-1,246	-0,318
KSHV-MIR-K12-10A	6,31E-01	-1,201	-0,264
MIR-641	6,33E-01	-1,259	-0,332
MIR-375	6,34E-01	-1,268	-0,343
MIR-877*	6,37E-01	-1,365	-0,449
MIR-1912	6,39E-01	-1,266	-0,341
MIR-1179	6,40E-01	-1,197	-0,259
MIR-92A-1*	6,41E-01	-1,206	-0,270
MIR-891A	6,41E-01	-1,299	-0,378
MIR-557	6,46E-01	-1,222	-0,289
MIR-300-3P	6,51E-01	-1,227	-0,295
MIR-650	6,55E-01	-1,211	-0,276
MIR-548D-5P	6,59E-01	-1,389	-0,474
MIR-181A*	6,59E-01	-1,306	-0,386
MIR-450B-5P	6,62E-01	1,608	0,685
MIR-760-3P	6,64E-01	-1,220	-0,286
MIR-448	6,65E-01	-1,196	-0,259
MIR-617	6,66E-01	-1,268	-0,343
MIR-151-3P	6,66E-01	-1,262	-0,336
MIR-18A*	6,66E-01	-1,272	-0,347
MIR-320D	6,66E-01	-1,279	-0,354
MIR-27B*	6,66E-01	-1,158	-0,212
MIR-659	6,68E-01	-1,185	-0,245
HBV-MIR-B20	6,70E-01	-1,247	-0,318
MIR-219-1-3P	6,70E-01	-1,199	-0,261
MIR-542-3P	6,70E-01	1,734	0,794
MIR-217	6,71E-01	-1,197	-0,259
MIR-767-3P	6,72E-01	-1,246	-0,317
MIR-1471	6,72E-01	-1,600	-0,678
MIR-200C	6,74E-01	-1,225	-0,293
EBV-MIR-BART4	6,76E-01	-1,293	-0,371
MIR-548C-3P	6,82E-01	-1,247	-0,318
MIR-551B	6,83E-01	-1,305	-0,384
MIR-182_1	6,84E-01	-1,216	-0,283
MIR-548K	6,85E-01	-1,245	-0,316
EBV-MIR-BART5	6,87E-01	-1,306	-0,385
KSHV-MIR-K12-9	6,91E-01	-1,249	-0,321
MIR-193A-3P	6,91E-01	1,345	0,428
MIR-425*	6,91E-01	-1,313	-0,392

MIR-558	6,92E-01	1,475	0,561
MIR-1178	6,92E-01	-1,282	-0,358
MIR-584	6,92E-01	-1,203	-0,267
MIR-374B*-374C*	6,93E-01	-1,222	-0,289
MIR-377	6,94E-01	1,500	0,585
MIR-654-3P	6,94E-01	-1,298	-0,376
MIR-205*	6,96E-01	-1,188	-0,248
MIR-551A	6,97E-01	-1,232	-0,301
MIR-493*	7,00E-01	1,662	0,733
MIR-548O	7,02E-01	-1,186	-0,247
MIR-629*	7,03E-01	-1,334	-0,415
MIR-362-3P	7,05E-01	-1,443	-0,529
MIR-145	7,07E-01	-1,229	-0,297
MIR-455-3P	7,10E-01	-1,237	-0,307
MIR-1272	7,11E-01	-1,238	-0,308
MIR-200A*	7,13E-01	-1,303	-0,381
MIR-505	7,15E-01	-1,228	-0,296
MIR-549	7,18E-01	-1,179	-0,238
MIR-1261	7,21E-01	-1,288	-0,365
MIR-548A-5P	7,21E-01	-1,170	-0,226
MIR-330-3P	7,21E-01	-1,185	-0,245
MIR-9*	7,22E-01	1,429	0,515
MIR-1292	7,26E-01	-1,262	-0,336
MIR-346	7,26E-01	-1,217	-0,283
MIR-223*	7,28E-01	-1,164	-0,219
LET-7D	7,28E-01	-1,154	-0,207
MIR-493	7,33E-01	-1,238	-0,308
MIR-24	7,33E-01	-1,164	-0,219
MIR-876-3P	7,34E-01	1,598	0,676
MIR-297A	7,34E-01	-1,215	-0,281
MIR-21*	7,35E-01	1,785	0,836
MIR-301A	7,35E-01	-1,284	-0,360
MIR-1185	7,37E-01	1,151	0,202
MIR-335*	7,38E-01	-1,222	-0,289
MIR-1238	7,40E-01	1,493	0,579
EBV-MIR-BHRF1-2	7,41E-01	1,734	0,794
MIR-2116	7,42E-01	-1,286	-0,363
MIR-337-3P	7,47E-01	1,666	0,737
MIR-155*	7,48E-01	-1,218	-0,284
MIR-551B*	7,49E-01	-1,266	-0,340
MIR-604	7,50E-01	-1,235	-0,305
MIR-411	7,51E-01	-1,207	-0,271
MIR-299-3P	7,52E-01	-1,208	-0,273
MIR-29C*	7,52E-01	-1,201	-0,265
MIR-194	7,52E-01	-1,268	-0,342
MIR-191	7,53E-01	-1,197	-0,259
MIR-137	7,56E-01	1,590	0,669
MIR-369-3P	7,57E-01	-1,207	-0,271
MIR-628-5P	7,57E-01	-1,250	-0,322
MIR-409-3P	7,57E-01	-1,200	-0,263
MIR-516A-3P-MIR-516*	7,57E-01	1,379	0,464
HCMV-MIR-UL22A	7,57E-01	-1,173	-0,231
HSV1-MIR-H7	7,58E-01	-1,274	-0,349
MIR-2054	7,58E-01	-1,216	-0,282
LET-7F-1*	7,61E-01	-1,203	-0,267
MIR-513A-5P	7,61E-01	-1,418	-0,504
MIR-127	7,61E-01	-1,164	-0,219
KSHV-MIR-K12-4-5P	7,63E-01	-1,245	-0,316
HIV1-MIR-TAR-3P	7,63E-01	-1,564	-0,645

MIR-7-2*	7,64E-01	-1,207	-0,271
MIR-520D-3P	7,64E-01	-1,176	-0,234
MIR-150	7,65E-01	-1,206	-0,270
MIR-569	7,66E-01	1,603	0,681
MIR-212	7,66E-01	-1,214	-0,280
MIR-152	7,69E-01	-1,198	-0,261
MIR-610	7,69E-01	-1,194	-0,255
MIR-627	7,70E-01	-1,284	-0,361
MIR-1303	7,72E-01	-1,206	-0,270
MIR-2117	7,77E-01	-1,226	-0,294
MIR-1908	7,78E-01	-1,283	-0,359
EBV-MIR-BART9	7,78E-01	-1,165	-0,220
MIR-191*	7,79E-01	-1,256	-0,329
MIR-922	7,79E-01	-1,266	-0,340
MIR-1287	7,80E-01	-1,253	-0,325
MIR-371-3P	7,82E-01	-1,190	-0,251
HSV1-MIR-H4*	7,83E-01	-1,147	-0,198
EBV-MIR-BHRF1-2*	7,83E-01	-1,354	-0,437
MIR-29B-1*	7,84E-01	-1,219	-0,286
MIR-1206	7,85E-01	-1,164	-0,219
LET-7F-2*	7,87E-01	-1,228	-0,296
MIR-1295	7,87E-01	-1,193	-0,254
MIR-548F-MIR-548G	7,88E-01	-1,176	-0,234
SV40-MIR-S1-3P	7,89E-01	-1,200	-0,264
MIR-34B-5P	7,92E-01	-1,199	-0,261
MIR-888*	7,92E-01	-1,159	-0,213
MIR-455-5P	7,93E-01	-1,196	-0,258
MIR-1280	7,93E-01	-1,430	-0,516
MIR-513B	7,94E-01	1,593	0,672
MIR-548D-3P	7,97E-01	1,375	0,460
MIR-9	7,98E-01	1,633	0,707
MIR-449C	7,99E-01	1,501	0,585
EBV-MIR-BART19-5P	8,01E-01	-1,202	-0,266
MIR-509-3-5P	8,02E-01	-1,144	-0,194
MIR-133A	8,03E-01	-1,233	-0,302
MIR-454*	8,03E-01	1,438	0,524
MIR-548P-548AM	8,04E-01	-1,190	-0,251
EBV-MIR-BART2-3P	8,05E-01	-1,165	-0,220
MIR-1256	8,08E-01	-1,148	-0,199
MIR-100*	8,08E-01	-1,162	-0,216
EBV-MIR-BART14-5P	8,08E-01	-1,237	-0,307
MIR-380-3P	8,09E-01	1,530	0,613
MIR-32	8,12E-01	1,534	0,618
MIR-1913	8,13E-01	-1,363	-0,447
MIR-1289	8,16E-01	-1,180	-0,238
MIR-299-5P	8,16E-01	-1,206	-0,270
MIR-29A	8,17E-01	1,180	0,238
MIR-200B*	8,17E-01	-1,299	-0,378
MIR-216B	8,18E-01	-1,230	-0,299
MIR-1296	8,19E-01	-1,221	-0,288
MIR-129-3P	8,19E-01	-1,221	-0,288
MIR-633	8,21E-01	1,523	0,607
MIR-218-1*	8,21E-01	-1,240	-0,311
MIR-499-3P	8,22E-01	-1,141	-0,191
MIR-802	8,23E-01	1,584	0,663
MIR-145*	8,23E-01	-1,189	-0,249
MIR-133B	8,27E-01	-1,222	-0,290
MIR-548B-3P	8,28E-01	1,406	0,491
MIR-1915	8,28E-01	-1,214	-0,280

MIR-367*	8,28E-01	-1,214	-0,280
MIR-1224-3P	8,28E-01	-1,261	-0,335
MIR-302B*	8,28E-01	1,369	0,453
MIR-25	8,28E-01	-1,219	-0,286
MIR-1268-1268B	8,29E-01	-1,159	-0,213
JCV-MIR-J1-3P-BKV-MIR-B1-3P	8,29E-01	-1,144	-0,194
MIR-497*	8,29E-01	-1,136	-0,184
MIR-365	8,29E-01	1,552	0,634
MIR-514	8,31E-01	1,505	0,590
MIR-21	8,31E-01	-1,157	-0,211
MIR-206	8,31E-01	-1,303	-0,382
MIR-202-3P	8,32E-01	-1,175	-0,232
MIR-504	8,35E-01	-1,162	-0,217
MIR-22*	8,37E-01	1,239	0,309
MIR-302A	8,37E-01	1,240	0,310
MIR-499-5P	8,37E-01	1,497	0,582
KSHV-MIR-K12-6-3P	8,39E-01	-1,140	-0,189
MIR-876-5P	8,39E-01	1,433	0,519
MIR-559	8,40E-01	1,470	0,556
MIR-376A*	8,41E-01	1,437	0,523
MIR-548Q	8,45E-01	-1,250	-0,322
MIR-28-3P	8,47E-01	-1,210	-0,275
MIR-17	8,48E-01	1,169	0,225
MIR-613	8,50E-01	1,288	0,365
MIR-1914*	8,50E-01	-1,175	-0,233
MIR-218-2*	8,51E-01	-1,266	-0,341
MIR-138-1*	8,51E-01	-1,398	-0,483
MIR-367	8,52E-01	1,358	0,441
MIR-154	8,52E-01	-1,240	-0,311
HSV1-MIR-H5-3P	8,52E-01	-1,217	-0,283
MIR-507	8,52E-01	1,404	0,490
MIR-603	8,53E-01	1,459	0,545
MIR-130A	8,54E-01	-1,195	-0,257
MIR-138-2*	8,54E-01	-1,191	-0,253
EBV-MIR-BART22	8,55E-01	-1,143	-0,193
MIR-511	8,55E-01	1,349	0,432
MIR-1911	8,56E-01	-1,188	-0,249
MIR-101*	8,57E-01	-1,166	-0,221
MIR-581	8,57E-01	1,490	0,575
MIR-579	8,58E-01	1,390	0,475
MIR-2115*	8,59E-01	-1,184	-0,244
LET-7G*	8,59E-01	-1,239	-0,309
MIR-1288	8,60E-01	1,470	0,556
MIR-541-3P	8,61E-01	-1,238	-0,309
MIR-607	8,62E-01	1,448	0,534
MIR-148A	8,62E-01	1,296	0,374
MCV-MIR-M1-3P	8,63E-01	-1,246	-0,317
MIR-586	8,64E-01	1,374	0,458
MIR-624	8,65E-01	1,305	0,384
MIR-302E	8,66E-01	-1,314	-0,394
MIR-1972	8,66E-01	-1,338	-0,420
MIR-376B	8,66E-01	1,301	0,380
MIR-938	8,67E-01	-1,164	-0,220
MIR-1236	8,68E-01	-1,202	-0,265
HSV1-MIR-H8	8,69E-01	-1,255	-0,328
MIR-15B*	8,69E-01	-1,131	-0,177
MIR-1283	8,69E-01	-1,191	-0,252
MIR-24-1*	8,72E-01	-1,210	-0,275
MIR-517C	8,72E-01	1,347	0,430

MIR-1265	8,73E-01	-1,259	-0,332
MIR-129*	8,73E-01	1,522	0,606
MIR-302B	8,73E-01	1,366	0,450
MIR-93*	8,74E-01	-1,134	-0,181
MIR-578	8,74E-01	1,387	0,472
MIR-590-5P	8,74E-01	1,526	0,610
MIR-450A-5P	8,74E-01	1,378	0,463
HSV1-MIR-H2	8,75E-01	-1,389	-0,474
MIR-616*	8,76E-01	-1,149	-0,200
MIR-216A	8,76E-01	-1,194	-0,255
MIR-657	8,76E-01	-1,181	-0,240
MIR-153	8,77E-01	1,233	0,302
MIR-7	8,79E-01	1,434	0,520
MIR-369-5P	8,80E-01	-1,168	-0,224
MIR-143*	8,81E-01	-1,224	-0,291
MIR-224	8,81E-01	-1,150	-0,202
MIR-1470	8,81E-01	-1,317	-0,397
MIR-1255B	8,82E-01	-1,290	-0,367
EBV-MIR-BART21-5P	8,82E-01	-1,131	-0,178
MIR-33A	8,83E-01	1,140	0,190
MIR-92B	8,83E-01	-1,180	-0,239
MIR-582-5P	8,84E-01	1,376	0,461
MIR-92A	8,84E-01	1,232	0,301
MIR-520H	8,84E-01	1,382	0,467
MIR-30B	8,86E-01	-1,218	-0,284
MIR-224*	8,86E-01	-1,396	-0,481
MIR-563	8,86E-01	1,353	0,436
MIR-562	8,88E-01	1,350	0,433
MIR-513A-3P	8,88E-01	1,360	0,444
MIR-429	8,88E-01	1,286	0,363
MIR-620	8,88E-01	1,401	0,486
MIR-126-3P	8,89E-01	-1,217	-0,283
MIR-545	8,90E-01	1,387	0,472
MIR-33B*	8,90E-01	-1,130	-0,177
MIR-182_2	8,91E-01	-1,141	-0,190
MIR-451	8,92E-01	1,332	0,413
MIR-519A	8,93E-01	1,359	0,442
MIR-506	8,93E-01	-1,192	-0,253
MIR-1251	8,95E-01	-1,108	-0,148
MIR-374B-374C	8,95E-01	1,347	0,429
MIR-181C*	8,95E-01	-1,184	-0,243
MIR-193A-5P	8,95E-01	-1,277	-0,353
MIR-1825	8,95E-01	-1,310	-0,390
MIR-1258	8,95E-01	-1,140	-0,189
MIR-146A	8,96E-01	1,224	0,291
MIR-2278	8,96E-01	-1,223	-0,291
MIR-501-3P	8,96E-01	-1,408	-0,494
MIR-520A-5P	8,97E-01	-1,122	-0,166
MIR-519C-3P	8,98E-01	1,396	0,482
MIR-2114	8,99E-01	-1,270	-0,345
MIR-423-5P	8,99E-01	-1,258	-0,331
MIR-32*	9,01E-01	-1,120	-0,163
MIR-653	9,02E-01	1,319	0,400
LET-7A-2*	9,02E-01	-1,214	-0,280
MIR-132*	9,02E-01	1,359	0,443
BKV-MIR-B1-5P	9,03E-01	-1,239	-0,309
MIR-361-3P	9,03E-01	-1,318	-0,398
MIR-2113	9,03E-01	-1,109	-0,149
MIR-1234	9,04E-01	-1,242	-0,313

MIR-141	9,05E-01	-1,177	-0,235
MIR-488	9,06E-01	1,293	0,371
EBV-MIR-BART8-5P	9,07E-01	1,296	0,374
MIR-15A	9,08E-01	-1,184	-0,244
MIR-2277-3P	9,10E-01	-1,170	-0,226
MIR-1976	9,10E-01	-1,280	-0,356
MIR-34A*	9,11E-01	-1,159	-0,212
HSV1-MIR-H2*	9,11E-01	-1,308	-0,387
MIR-98	9,11E-01	-1,257	-0,329
MIR-1269	9,12E-01	-1,248	-0,320
MIR-663B	9,12E-01	-1,204	-0,268
MIR-574-3P	9,12E-01	-1,154	-0,207
LET-7G	9,12E-01	-1,106	-0,146
MIR-1249	9,12E-01	-1,210	-0,275
MIR-875-5P	9,12E-01	1,370	0,454
MIR-147A	9,13E-01	-1,169	-0,225
HCMV-MIR-UL22A*	9,14E-01	1,290	0,367
MIR-144	9,14E-01	1,388	0,473
HSV2-MIR-H4-3P	9,14E-01	1,374	0,458
MIR-1323	9,15E-01	-1,166	-0,222
MIR-7-1*	9,16E-01	-1,181	-0,240
MIR-2053	9,18E-01	-1,115	-0,157
MIR-545*	9,21E-01	-1,115	-0,157
MIR-1233	9,21E-01	-1,259	-0,332
LET-7F	9,21E-01	1,096	0,132
EBV-MIR-BART1-3P	9,23E-01	-1,134	-0,181
MIR-582-3P	9,24E-01	-1,148	-0,199
MIR-522	9,25E-01	1,258	0,331
MIR-379*	9,25E-01	1,277	0,352
MIR-524-5P	9,26E-01	-1,407	-0,493
MIR-764	9,26E-01	1,304	0,383
MIR-520G	9,27E-01	-1,131	-0,177
MIR-520E	9,27E-01	1,223	0,290
MIR-365B	9,27E-01	-1,144	-0,194
MIR-1254	9,27E-01	-1,227	-0,295
MIR-670	9,29E-01	1,185	0,245
MIR-30D	9,29E-01	-1,113	-0,155
MIR-181A-2*	9,29E-01	-1,108	-0,149
MIR-200A	9,30E-01	-1,205	-0,270
MIR-606	9,30E-01	-1,225	-0,293
MIR-890	9,31E-01	-1,135	-0,183
MIR-580	9,31E-01	1,370	0,454
MIR-1231	9,32E-01	-1,165	-0,220
MIR-125A-5P	9,33E-01	-1,140	-0,189
MIR-1263	9,34E-01	-1,197	-0,259
MIR-454_2	9,34E-01	1,229	0,297
MIR-10A	9,35E-01	-1,117	-0,159
MIR-34C-3P	9,37E-01	-1,176	-0,234
MIR-491-3P	9,38E-01	1,244	0,315
MIR-513C	9,38E-01	-1,137	-0,186
MIR-570	9,39E-01	1,270	0,345
MIR-517A-MIR-517B_1	9,39E-01	-1,139	-0,188
MIR-27A*	9,39E-01	-1,109	-0,149
MIR-195	9,40E-01	-1,151	-0,203
MIR-634	9,41E-01	-1,280	-0,357
MIR-196A*	9,42E-01	-1,225	-0,292
MIR-1207-3P	9,42E-01	-1,162	-0,216
MIR-1276	9,42E-01	1,198	0,260
MIR-519E	9,43E-01	1,250	0,322

MIR-518C	9,44E-01	1,181	0,240
MIR-548C-5P	9,44E-01	1,293	0,371
MIR-652	9,46E-01	1,132	0,179
MIR-144*	9,46E-01	-1,117	-0,160
MIR-515-5P	9,47E-01	1,269	0,343
LET-7E	9,49E-01	-1,078	-0,108
MIR-1911*	9,49E-01	1,292	0,369
MIR-1225-3P	9,50E-01	1,224	0,292
LET-7E*	9,50E-01	-1,168	-0,224
MIR-96	9,50E-01	1,198	0,260
MIR-1271	9,51E-01	-1,263	-0,337
MIR-181B	9,52E-01	-1,117	-0,160
MIR-139-3P	9,54E-01	1,337	0,419
MIR-331-3P	9,55E-01	-1,118	-0,160
MIR-34B-3P	9,55E-01	1,278	0,354
MIR-1264	9,55E-01	-1,150	-0,202
MIR-664-3P	9,56E-01	-1,155	-0,208
MIR-374A*	9,58E-01	-1,104	-0,142
MIR-1205	9,58E-01	-1,144	-0,195
MIR-26B	9,58E-01	-1,152	-0,204
MIR-30B*	9,59E-01	-1,168	-0,224
MIR-19B-2*	9,59E-01	-1,068	-0,094
MIR-1279	9,59E-01	-1,079	-0,110
MIR-888	9,60E-01	1,142	0,191
MIR-488*	9,60E-01	-1,124	-0,169
MIR-1322	9,61E-01	1,275	0,351
MIR-322*-MIR-424*	9,61E-01	-1,143	-0,192
MIR-525-3P	9,62E-01	-1,087	-0,120
MIR-548M	9,62E-01	-1,084	-0,117
MIR-1248	9,62E-01	-1,090	-0,125
MIR-1245	9,62E-01	1,155	0,208
MIR-452	9,63E-01	-1,099	-0,136
MIR-196B*	9,63E-01	1,288	0,365
MIR-31*	9,63E-01	-1,111	-0,151
MIR-34A	9,64E-01	1,107	0,146
MIR-146B-3P	9,65E-01	1,178	0,236
LET-7A*-LET-7C-2*	9,66E-01	-1,095	-0,131
MIR-891B	9,66E-01	1,171	0,228
MIR-421-5P	9,68E-01	1,226	0,293
MIR-190B	9,68E-01	-1,131	-0,178
MIR-195*	9,68E-01	1,204	0,268
MIR-649	9,69E-01	-1,110	-0,150
MIR-520A-3P	9,69E-01	-1,075	-0,104
HBV-MIR-B4	9,69E-01	-1,112	-0,153
MIR-2276	9,69E-01	-1,145	-0,195
MIR-214*	9,69E-01	-1,132	-0,179
MIR-10A*	9,69E-01	-1,169	-0,226
MIR-598-3P	9,70E-01	-1,116	-0,159
JCV-MIR-J1-5P	9,70E-01	-1,083	-0,116
MIR-1204	9,71E-01	1,255	0,328
MIR-1302	9,71E-01	-1,081	-0,112
MIR-449A	9,71E-01	-1,080	-0,111
HSV1-MIR-H3	9,72E-01	-1,127	-0,173
HSV2-MIR-H4-5P	9,72E-01	-1,139	-0,188
MIR-616	9,72E-01	-1,098	-0,135
MIR-106A*	9,72E-01	1,270	0,345
EBV-MIR-BART20-5P_3	9,72E-01	-1,083	-0,115
MIR-552	9,73E-01	-1,177	-0,236
MIR-744*	9,73E-01	-1,088	-0,122

MIR-599	9,73E-01	1,158	0,211
MIR-10B	9,73E-01	-1,098	-0,135
MIR-101-101C	9,73E-01	1,166	0,221
MIR-29B-2*	9,74E-01	-1,086	-0,118
MIR-219-5P	9,74E-01	-1,127	-0,173
MIR-325-3P	9,74E-01	1,120	0,163
MIR-1266	9,75E-01	1,229	0,297
MIR-196A	9,75E-01	1,117	0,160
MIR-127*	9,75E-01	-1,137	-0,185
MIR-561	9,75E-01	1,217	0,284
MIR-1277	9,75E-01	1,186	0,246
MIR-23A*	9,75E-01	-1,064	-0,090
MIR-136	9,76E-01	1,173	0,230
HSV1-MIR-H8*	9,76E-01	-1,118	-0,161
MIR-26B*	9,76E-01	-1,071	-0,099
MIR-1278	9,76E-01	1,105	0,144
MIR-539-5P	9,77E-01	1,125	0,171
MIR-186*	9,77E-01	-1,063	-0,088
MIR-940	9,78E-01	-1,116	-0,158
EBV-MIR-BART2-5P	9,78E-01	1,122	0,166
MIR-125B-1*	9,78E-01	1,094	0,129
MIR-1324	9,78E-01	-1,092	-0,127
MIR-892A	9,79E-01	1,134	0,182
MIR-1228	9,79E-01	-1,117	-0,160
MIR-340-3P	9,79E-01	-1,101	-0,138
MIR-147B	9,80E-01	-1,093	-0,128
EBV-MIR-BART21-3P	9,80E-01	-1,107	-0,147
MIR-218	9,80E-01	1,172	0,229
MIR-16	9,80E-01	-1,052	-0,073
MIR-1247	9,80E-01	-1,178	-0,236
MIR-92B*	9,81E-01	1,084	0,116
MIR-194*	9,81E-01	1,173	0,231
MIR-338-5P	9,82E-01	-1,065	-0,090
MIR-1284	9,82E-01	-1,135	-0,183
MIR-518F	9,82E-01	1,130	0,177
MIR-200C*	9,83E-01	1,148	0,199
MIR-609	9,83E-01	1,132	0,179
MIR-340-5P	9,83E-01	1,119	0,162
MIR-1285	9,83E-01	-1,113	-0,155
MIR-374A	9,83E-01	1,148	0,199
MIR-338-3P	9,83E-01	1,173	0,230
MIR-130B*	9,84E-01	-1,055	-0,077
LET-7B	9,84E-01	-1,079	-0,110
MIR-124*	9,84E-01	-1,123	-0,167
HSV2-MIR-H2	9,84E-01	1,178	0,237
MIR-505*	9,84E-01	-1,169	-0,225
MIR-335	9,84E-01	1,126	0,171
MIR-1293	9,85E-01	-1,061	-0,085
MIR-1281	9,85E-01	-1,137	-0,185
MIR-920	9,85E-01	-1,123	-0,167
EBV-MIR-BART18-5P	9,85E-01	1,149	0,200
MIR-1299	9,85E-01	-1,083	-0,115
MIR-221	9,86E-01	1,062	0,086
MIR-508-3P	9,86E-01	1,073	0,101
MIR-10B*	9,87E-01	-1,049	-0,070
MIR-889	9,87E-01	1,151	0,203
MIR-26A	9,87E-01	-1,047	-0,066
MIR-15A*	9,87E-01	1,164	0,219
MIR-135B	9,88E-01	1,160	0,214

MIR-625*	9,88E-01	-1,122	-0,165
MIR-577	9,88E-01	-1,099	-0,137
MIR-203	9,88E-01	-1,068	-0,095
MIR-1203	9,88E-01	1,160	0,214
MIR-548L	9,89E-01	1,156	0,209
MIR-1244	9,89E-01	-1,053	-0,075
MIR-122*	9,89E-01	1,089	0,123
MIR-1255A	9,89E-01	-1,059	-0,082
MIR-2116*	9,89E-01	-1,122	-0,167
MIR-941	9,89E-01	-1,068	-0,095
MIR-2114*	9,89E-01	-1,098	-0,135
HSV2-MIR-H3	9,89E-01	1,053	0,074
MIR-208B	9,90E-01	1,142	0,192
MIR-924	9,90E-01	-1,058	-0,082
MIR-1A	9,90E-01	1,171	0,228
MIR-30A	9,90E-01	-1,071	-0,099
MIR-410	9,90E-01	-1,078	-0,108
EBV-MIR-BART15	9,90E-01	-1,063	-0,088
MIR-192*	9,91E-01	1,133	0,180
MIR-376C	9,91E-01	1,093	0,128
MIR-585	9,91E-01	1,082	0,114
MIR-24-2*	9,91E-01	-1,142	-0,192
EBV-MIR-BART4*	9,91E-01	1,140	0,189
MIR-222*	9,91E-01	-1,104	-0,143
MIR-376A	9,91E-01	1,076	0,106
MIR-618	9,91E-01	1,141	0,190
MIR-943	9,91E-01	-1,130	-0,176
MIR-486-3P	9,91E-01	1,136	0,184
MIR-496_2	9,91E-01	1,075	0,105
MIR-26A-1*	9,91E-01	1,076	0,106
MIR-187*	9,92E-01	1,141	0,190
MIR-520C-3P-520F-520B_4	9,92E-01	1,107	0,147
MIR-30C-2*	9,92E-01	1,095	0,131
LET-7I*	9,92E-01	-1,049	-0,068
EBV-MIR-BART19-3P	9,92E-01	1,131	0,177
MIR-18B*	9,92E-01	1,158	0,211
MIR-592	9,93E-01	1,067	0,094
MIR-1250	9,93E-01	1,123	0,167
MIR-16-1*	9,93E-01	-1,102	-0,140
MIR-1226*	9,93E-01	-1,085	-0,118
MIR-208A	9,93E-01	-1,074	-0,103
MIR-576-3P	9,94E-01	1,082	0,114
MIR-16-2*	9,94E-01	-1,095	-0,131
MIR-154*	9,94E-01	1,082	0,114
MIR-29B	9,94E-01	-1,048	-0,067
MIR-342-5P	9,94E-01	-1,166	-0,221
EBV-MIR-BART13*	9,95E-01	1,113	0,154
MIR-302D*	9,95E-01	1,086	0,118
MIR-626	9,95E-01	1,098	0,134
MIR-141*	9,95E-01	-1,036	-0,051
MCV-MIR-M1-5P	9,95E-01	-1,073	-0,101
HSV1-MIR-H7*	9,95E-01	-1,094	-0,129
MIR-105*	9,95E-01	-1,078	-0,108
MIR-1307	9,96E-01	1,100	0,137
MIR-449C*	9,96E-01	-1,075	-0,105
MIR-1182	9,96E-01	-1,068	-0,094
MIR-1243	9,96E-01	-1,029	-0,042
MIR-325-5P	9,96E-01	1,049	0,069
MIR-452*	9,96E-01	1,059	0,083

MIR-384-5P	9,96E-01	-1,062	-0,087
MIR-944	9,97E-01	-1,045	-0,064
MIR-146A*	9,97E-01	-1,060	-0,084
LET-7D*	9,97E-01	1,073	0,102
MIR-329	9,97E-01	-1,037	-0,053
MIR-526B	9,97E-01	-1,047	-0,067
MIR-148A*	9,97E-01	-1,055	-0,077
MIR-519D	9,97E-01	-1,053	-0,075
MIR-624*	9,97E-01	1,049	0,070
MIR-1915*	9,97E-01	-1,089	-0,123
MIR-759	9,97E-01	-1,052	-0,073
MIR-553	9,98E-01	-1,070	-0,098
MIR-1286	9,98E-01	-1,053	-0,075
MIR-181A	9,98E-01	-1,058	-0,081
MIR-651	9,98E-01	-1,067	-0,093
MIR-2115	9,98E-01	-1,060	-0,084
MIR-548A-3P	9,98E-01	1,070	0,097
MIR-1306-3P	9,98E-01	-1,033	-0,046
MIR-29A*	9,98E-01	-1,047	-0,066
MIR-708*	9,98E-01	1,056	0,078
MIR-544	9,98E-01	1,037	0,052
MIR-95	9,98E-01	1,054	0,076
MIR-519B-3P	9,98E-01	1,059	0,083
MIR-28-5P-28C	9,98E-01	-1,048	-0,068
MIR-664-5P	9,98E-01	-1,036	-0,052
MIR-1202	9,98E-01	1,090	0,124
MIR-495	9,98E-01	-1,039	-0,055
MIR-99B*	9,98E-01	-1,055	-0,078
MIR-363	9,99E-01	-1,028	-0,040
MIR-548N	9,99E-01	1,028	0,040
MIR-18A	9,99E-01	-1,038	-0,054
MIR-302D	9,99E-01	1,037	0,053
MIR-431*	9,99E-01	-1,036	-0,051
MIR-221*	9,99E-01	1,065	0,091
MIR-1539	9,99E-01	1,062	0,087
MIR-1227	9,99E-01	1,028	0,040
MIR-19A*	9,99E-01	1,026	0,036
MIR-20B*	9,99E-01	-1,027	-0,038
MIR-921	9,99E-01	-1,052	-0,074
MIR-215	9,99E-01	-1,031	-0,044
MIR-1304	9,99E-01	-1,022	-0,032
MIR-192	9,99E-01	-1,029	-0,042
MIR-1229	9,99E-01	1,050	0,071
MIR-1180	9,99E-01	1,036	0,051
MIR-656	9,99E-01	-1,043	-0,060
MIR-1200	9,99E-01	-1,051	-0,072
EBV-MIR-BART3-5P	9,99E-01	-1,027	-0,038
MIR-126-5P	9,99E-01	-1,029	-0,041
MIR-642A	9,99E-01	-1,024	-0,035
MIR-33*	9,99E-01	-1,021	-0,030
MIR-302C	1,00E+00	1,028	0,040
LET-7B*	1,00E+00	1,055	0,078
MIR-185*	1,00E+00	-1,025	-0,036
EBV-MIR-BART5*	1,00E+00	-1,057	-0,079
MIR-20A*	1,00E+00	-1,031	-0,044
HSV1-MIR-H6-3P	1,00E+00	1,043	0,060
MIR-675*	1,00E+00	1,042	0,060
MIR-1184	1,00E+00	-1,040	-0,057
MIR-106A	1,00E+00	1,013	0,019

KSHV-MIR-K12-9*	1,00E+00	-1,022	-0,032
MIR-1298	1,00E+00	1,017	0,024
MIR-449B*	1,00E+00	1,036	0,051
MIR-106B*	1,00E+00	1,049	0,069
MIR-301B	1,00E+00	1,024	0,034
MIR-548B-5P	1,00E+00	-1,018	-0,025
EBV-MIR-BART9*	1,00E+00	-1,035	-0,050
HBV-MIR-B2RC	1,00E+00	-1,018	-0,025
MIR-576-5P	1,00E+00	-1,020	-0,029
MIR-199B-5P	1,00E+00	1,021	0,031
MIR-135A	1,00E+00	1,017	0,025
HCMV-MIR-UL36*	1,00E+00	-1,022	-0,032
MIR-188-3P	1,00E+00	1,018	0,026
LET-7I	1,00E+00	-1,012	-0,017
MIR-199A-3P-199B-3P	1,00E+00	1,015	0,021
MIR-591	1,00E+00	-1,015	-0,021
MIR-103B	1,00E+00	-1,019	-0,027
MIR-148B*	1,00E+00	-1,021	-0,030
MIR-1237	1,00E+00	1,014	0,020
MIR-521	1,00E+00	1,012	0,017
MIR-655	1,00E+00	-1,012	-0,017
MIR-219-2-3P	1,00E+00	-1,011	-0,016
MIR-589	1,00E+00	1,012	0,017
MIR-196B	1,00E+00	1,011	0,016
MIR-526B*	1,00E+00	-1,012	-0,017
MIR-583	1,00E+00	-1,011	-0,016
MIR-204	1,00E+00	1,008	0,011
MIR-17*	1,00E+00	-1,007	-0,010
MIR-125B-2*	1,00E+00	-1,016	-0,023
MIR-103A-2*	1,00E+00	1,011	0,016
MIR-377*	1,00E+00	-1,007	-0,010
MIR-1282	1,00E+00	-1,007	-0,010
MIR-644	1,00E+00	-1,008	-0,012
MIR-1291	1,00E+00	1,009	0,013
MIR-1226	1,00E+00	-1,014	-0,020
MIR-337-3P	1,00E+00	-1,006	-0,008
EBV-MIR-BART7*	1,00E+00	-1,008	-0,011
MIR-182*	1,00E+00	1,004	0,006
EBV-MIR-BART10*	1,00E+00	1,006	0,008
MIR-135B*	1,00E+00	1,010	0,014
MIR-302A*	1,00E+00	-1,003	-0,004
MIR-186	1,00E+00	-1,003	-0,004
MIR-1181	1,00E+00	1,002	0,003
MIR-211	1,00E+00	-1,001	-0,002
HCMV-MIR-US25-1*	1,00E+00	-1,001	-0,001
MIR-23B*	1,00E+00	1,001	0,002
MIR-632	1,00E+00	-1,000	0,000
