# Supplementary material for: Effector and regulatory dendritic cells display distinct patterns of miRNA expression
Source: Immun Inflamm Dis. 2017 May 12;5(3):310–7. doi: 10.1002/iid3.165 (PMC5569363; doi:10.1002/iid3.165)
Supplement: Supplementary file 4 — DC2. Microarray results DC2 vs Unstimulated DCs [file IID3-5-310-s004.pdf]

	DC2 vs Unstimulated DC		
miRNA name	Tukey.p-value	Fold_Change	Log2_Ratio
MIR-663	0,00E+00	-5,247	-2,392
MIR-155	0,00E+00	29,557	4,885
MIR-762	2,22E-15	-4,345	-2,119
MIR-638	3,31E-07	-3,892	-1,960
MIR-1469	1,25E-05	-6,358	-2,669
MIR-1908	1,53E-04	-3,015	-1,592
MIR-744	3,17E-04	-1,868	-0,901
MIR-132	7,07E-04	2,871	1,522
MIR-422A	1,61E-03	-2,378	-1,249
MIR-494	1,48E-02	-4,031	-2,011
MIR-1228*	1,52E-02	-3,001	-1,585
MIR-487B	1,78E-02	-1,627	-0,703
MIR-1246	3,01E-02	-2,800	-1,485
MIR-339-5P	3,01E-02	-1,943	-0,959
MIR-423-3P	5,32E-02	-1,546	-0,629
MIR-1275	5,50E-02	-3,064	-1,615
MIR-942	5,99E-02	-1,609	-0,686
MIR-148B	6,22E-02	-2,014	-1,010
MIR-149*	6,63E-02	-2,202	-1,139
MIR-548K	6,90E-02	-1,622	-0,697
MIR-1909	7,35E-02	-2,860	-1,516
MIR-371-5P	7,73E-02	-1,752	-0,809
MIR-593	7,81E-02	-1,664	-0,734
MIR-1228	8,75E-02	-1,926	-0,945
MIR-1231	9,25E-02	-1,809	-0,855
MIR-342-3P	1,01E-01	-1,395	-0,480
MIR-935	1,01E-01	-1,374	-0,458
MIR-1973	1,07E-01	-4,192	-2,068
MIR-378	1,10E-01	-1,527	-0,611
MIR-939	1,23E-01	-1,518	-0,602
HIV1-MIR-TAR-5P	1,31E-01	-1,414	-0,500
MIR-934	1,41E-01	-1,351	-0,434
MIR-34A	1,49E-01	1,567	0,648
MIR-1268-1268B	1,67E-01	-1,427	-0,513
MIR-505	1,67E-01	1,491	0,577
MIR-92A-1*	1,72E-01	-1,385	-0,470
MIR-150*	1,72E-01	-2,236	-1,161
MIR-933	1,91E-01	-1,455	-0,541
MIR-548A-5P	1,94E-01	-1,345	-0,428
MIR-628-5P	2,00E-01	-1,562	-0,643
MIR-1252	2,02E-01	-1,412	-0,498
MIR-302F	2,07E-01	-1,416	-0,502
MIR-29A	2,14E-01	1,440	0,526
LET-7E	2,21E-01	1,304	0,383
MIR-1537	2,21E-01	-1,411	-0,497
MIR-1296	2,22E-01	-1,548	-0,631
MIR-1287	2,26E-01	-1,574	-0,654
MIR-135A*	2,29E-01	-1,418	-0,504
MIR-130A*	2,39E-01	-1,644	-0,717
MIR-1260	2,41E-01	-2,051	-1,036
MIR-182_2	2,44E-01	-1,409	-0,495
MIR-1912	2,50E-01	-1,448	-0,534
MIR-1273	2,57E-01	-1,540	-0,623
MIR-212	2,59E-01	1,442	0,528
MIR-19A	2,63E-01	-1,642	-0,715
MIR-1179	2,66E-01	-1,319	-0,399
MIR-219-1-3P	2,69E-01	-1,336	-0,418

MIR-613	2,71E-01	1,758	0,814
MIR-663B	2,72E-01	-1,661	-0,732
MIR-196A	2,83E-01	1,597	0,676
LET-7A	2,86E-01	1,577	0,658
MIR-193B*	2,89E-01	-1,553	-0,635
MIR-196B	2,94E-01	1,655	0,727
MIR-1468	2,98E-01	-1,303	-0,382
MIR-1282	3,05E-01	-1,581	-0,660
MIR-499-3P	3,10E-01	-1,301	-0,380
MIR-891B	3,12E-01	1,791	0,841
MIR-33A	3,23E-01	1,356	0,439
MIR-26A-2*	3,25E-01	-1,527	-0,611
MIR-708*	3,25E-01	1,723	0,785
MIR-326	3,27E-01	-1,379	-0,464
MIR-302A	3,28E-01	1,544	0,626
MIR-25*	3,28E-01	-1,303	-0,382
MIR-15B	3,29E-01	-1,287	-0,364
MIR-373*	3,30E-01	-1,316	-0,396
MIR-548P-548AM	3,37E-01	-1,382	-0,467
MIR-33B*	3,39E-01	-1,330	-0,412
MIR-1183	3,40E-01	-1,461	-0,547
HSV1-MIR-H4*	3,41E-01	-1,276	-0,352
MIR-328-5P	3,51E-01	-1,977	-0,984
MIR-1224-5P	3,53E-01	-1,394	-0,479
MIR-943	3,56E-01	-1,970	-0,978
MIR-1294	3,57E-01	-1,324	-0,404
EBV-MIR-BART18-5P	3,58E-01	1,904	0,929
MIR-199A-3P-199B-3P	3,59E-01	1,536	0,619
MIR-20B	3,63E-01	-1,769	-0,823
MIR-425	3,67E-01	-1,330	-0,411
EBV-MIR-BART2-5P	3,70E-01	1,585	0,665
MIR-1267	3,71E-01	-1,480	-0,566
MIR-876-5P	3,73E-01	2,005	1,004
HSV1-MIR-H4	3,75E-01	-1,277	-0,352
MIR-611	3,76E-01	-1,349	-0,431
MIR-378*	3,81E-01	-1,379	-0,464
HCMV-MIR-US25-1*	3,87E-01	-1,354	-0,438
MIR-451	3,91E-01	1,888	0,917
MIR-34B-3P	3,91E-01	2,114	1,080
MIR-876-3P	3,95E-01	2,049	1,035
MIR-302B*	4,00E-01	1,776	0,828
MIR-301B	4,02E-01	1,547	0,629
MIR-1301	4,04E-01	-1,420	-0,506
MIR-1257	4,05E-01	-1,248	-0,320
HCMV-MIR-US33-5P	4,11E-01	-1,290	-0,367
MIR-570	4,17E-01	1,887	0,916
MIR-1225-5P	4,18E-01	-1,409	-0,495
MIR-1279	4,21E-01	-1,263	-0,337
MIR-1915	4,23E-01	-1,413	-0,499
MIR-548E	4,24E-01	-1,284	-0,361
MIR-127*	4,27E-01	-1,590	-0,669
HIV1-MIR-TAR-3P	4,27E-01	-2,003	-1,002
MIR-223	4,28E-01	-1,250	-0,322
MIR-548F-MIR-548G	4,34E-01	-1,300	-0,379
MIR-335*	4,36E-01	-1,344	-0,426
HSV1-MIR-H5-3P	4,36E-01	-1,443	-0,529
MIR-624*	4,37E-01	1,457	0,543
MIR-154*	4,39E-01	1,575	0,655
LET-7F	4,39E-01	1,241	0,312

MIR-662	4,40E-01	-1,274	-0,349
MIR-1256	4,41E-01	-1,259	-0,333
MIR-155*	4,44E-01	-1,340	-0,422
MIR-1914	4,47E-01	-1,679	-0,748
MIR-142-3P	4,48E-01	-1,679	-0,747
MIR-146A	4,50E-01	1,529	0,612
MIR-135B	4,54E-01	1,942	0,957
MIR-552	4,57E-01	1,739	0,798
MIR-616*	4,58E-01	1,312	0,391
MIR-937	4,61E-01	-1,343	-0,425
MIR-1248	4,61E-01	-1,297	-0,376
MIR-32*	4,62E-01	-1,270	-0,345
MIR-497*	4,62E-01	-1,244	-0,315
MIR-483-5P	4,63E-01	-1,476	-0,562
MIR-548M	4,64E-01	-1,273	-0,349
MIR-221	4,64E-01	-1,288	-0,365
MIR-1253	4,64E-01	-1,593	-0,672
MIR-185	4,66E-01	-1,385	-0,470
MIR-1208	4,66E-01	-1,241	-0,312
MIR-491-3P	4,67E-01	1,727	0,788
EBV-MIR-BART12	4,68E-01	-1,324	-0,405
MIR-545*	4,70E-01	-1,282	-0,358
MIR-125A-3P	4,71E-01	-1,646	-0,719
MIR-374A*	4,72E-01	-1,325	-0,406
MIR-514	4,73E-01	2,001	1,000
MIR-508-3P	4,74E-01	1,340	0,423
MIR-302A*	4,75E-01	1,450	0,536
MIR-376A*	4,75E-01	1,878	0,909
EBV-MIR-BART8-5P	4,78E-01	1,737	0,796
MIR-376B	4,79E-01	1,628	0,703
HSV1-MIR-H1	4,80E-01	-1,358	-0,442
MIR-7	4,87E-01	1,987	0,990
MIR-1234	4,88E-01	-1,570	-0,650
MIR-595	4,92E-01	-1,300	-0,379
MIR-1297	4,92E-01	-1,260	-0,334
MIR-888	4,94E-01	1,456	0,542
MIR-511	4,94E-01	1,694	0,761
MIR-548O	4,95E-01	-1,249	-0,321
MIR-1262	4,96E-01	-1,279	-0,355
MIR-328-3P	4,96E-01	-1,283	-0,359
MIR-323-5P	4,97E-01	-1,284	-0,360
MIR-429	4,97E-01	1,629	0,704
MIR-520E	5,00E-01	1,579	0,659
MIR-320B	5,02E-01	-1,300	-0,379
MIR-518E*-519A*-1-519B-5P-519C-5P-522*-523*-526C	5,07E-01	-1,239	-0,310
MIR-519A	5,09E-01	1,818	0,863
MIR-20A	5,09E-01	1,415	0,500
MIR-130B	5,10E-01	-1,224	-0,292
MIR-130B*	5,11E-01	-1,225	-0,293
EBV-MIR-BART22	5,12E-01	-1,259	-0,333
MIR-421-3P	5,17E-01	-1,199	-0,262
MIR-296-5P	5,17E-01	-1,345	-0,428
MIR-1299	5,18E-01	-1,369	-0,453
MIR-1251	5,21E-01	-1,219	-0,286
MIR-376C	5,22E-01	1,495	0,580
MIR-148A	5,24E-01	1,570	0,651
MIR-548B-3P	5,26E-01	1,716	0,779
MIR-9*	5,26E-01	1,588	0,668
MIR-93*	5,29E-01	-1,252	-0,324

MIR-569	5,32E-01	1,925	0,945
EBV-MIR-BART17-3P	5,32E-01	-1,257	-0,330
MIR-1206	5,32E-01	-1,245	-0,316
MIR-96	5,32E-01	1,574	0,655
MIR-1538	5,33E-01	-1,467	-0,553
MIR-519E	5,33E-01	1,708	0,773
MIR-324-3P	5,33E-01	-1,285	-0,362
MIR-664-5P	5,34E-01	-1,342	-0,424
MIR-1178	5,34E-01	-1,356	-0,439
MIR-183*	5,36E-01	-1,378	-0,463
MIR-22*	5,36E-01	1,411	0,496
MIR-654-5P	5,36E-01	-1,277	-0,352
MIR-1323	5,38E-01	-1,373	-0,457
MIR-1321	5,38E-01	-1,267	-0,341
MIR-30E*	5,39E-01	-1,217	-0,283
MIR-522	5,39E-01	1,638	0,712
MIR-517C	5,42E-01	1,685	0,752
MIR-325-3P	5,44E-01	1,426	0,512
HCMV-MIR-UL112	5,46E-01	-1,256	-0,328
EBV-MIR-BART21-5P	5,47E-01	-1,248	-0,319
MIR-1255B	5,47E-01	-1,580	-0,660
MIR-1302	5,47E-01	-1,260	-0,334
MIR-519C-5P	5,52E-01	-1,260	-0,333
MIR-454*	5,52E-01	1,697	0,763
MIR-520H	5,54E-01	1,788	0,838
MIR-759	5,55E-01	1,408	0,493
MIR-558	5,60E-01	1,587	0,666
MIR-320A	5,61E-01	-1,275	-0,351
MIR-631	5,62E-01	-1,355	-0,439
MIR-182*	5,67E-01	1,344	0,426
MIR-1293	5,67E-01	-1,240	-0,310
MIR-507	5,68E-01	1,726	0,787
MIR-539-5P	5,68E-01	1,453	0,539
MIR-892A	5,70E-01	1,503	0,588
MIR-675	5,71E-01	-1,288	-0,365
EBV-MIR-BHRF1-2	5,73E-01	2,007	1,005
MIR-642A	5,73E-01	1,301	0,379
MIR-2052	5,75E-01	-1,242	-0,313
MIR-596	5,79E-01	-1,247	-0,319
MIR-563	5,82E-01	1,694	0,760
MIR-568	5,84E-01	-1,366	-0,450
MIR-1289	5,88E-01	-1,268	-0,342
MIR-377	5,90E-01	1,595	0,674
MIR-23A*	5,90E-01	-1,207	-0,272
MIR-1270	5,91E-01	-1,277	-0,353
MIR-208B	5,92E-01	1,712	0,776
MIR-542-3P	5,92E-01	1,841	0,880
MIR-516A-3P-MIR-516*	5,94E-01	1,504	0,589
MIR-367	5,94E-01	1,609	0,686
MIR-153	5,95E-01	1,418	0,504
MIR-27B*	5,96E-01	-1,175	-0,233
MIR-92A-2*	5,98E-01	-1,344	-0,427
MIR-488	5,99E-01	1,605	0,682
MIR-875-5P	5,99E-01	1,811	0,857
MIR-1197	6,00E-01	-1,318	-0,398
MIR-92B	6,00E-01	-1,323	-0,404
MIR-222	6,01E-01	1,376	0,461
MIR-302D	6,04E-01	1,348	0,431
MIR-454_2	6,06E-01	1,535	0,619

HCMV-MIR-UL22A*	6,08E-01	1,611	0,688
MIR-519C-3P	6,10E-01	1,800	0,848
MIR-2053	6,10E-01	-1,231	-0,300
MIR-10B*	6,12E-01	-1,191	-0,252
MIR-548H	6,13E-01	-1,279	-0,356
MIR-10B	6,13E-01	1,304	0,383
MIR-625*	6,13E-01	-1,529	-0,613
MIR-1911	6,13E-01	-1,303	-0,382
MIR-15B*	6,17E-01	-1,215	-0,281
MIR-205*	6,19E-01	-1,211	-0,277
MIR-380-5P	6,19E-01	-1,247	-0,318
MIR-370	6,21E-01	-1,239	-0,309
MIR-554	6,24E-01	-1,457	-0,543
MIR-766	6,25E-01	-1,346	-0,428
MIR-579	6,25E-01	1,647	0,720
MIR-450B-3P	6,28E-01	-1,312	-0,392
MIR-337-3P	6,30E-01	1,317	0,398
MIR-452*	6,31E-01	1,370	0,454
MIR-203	6,31E-01	1,272	0,347
MIR-128	6,33E-01	-1,244	-0,315
MIR-877	6,36E-01	-1,348	-0,431
MIR-1264	6,38E-01	-1,379	-0,464
MIR-640	6,38E-01	-1,252	-0,325
LET-7F-1*	6,38E-01	-1,250	-0,322
MIR-802	6,39E-01	1,873	0,905
MIR-1290	6,40E-01	-1,180	-0,239
MIR-135A	6,41E-01	1,341	0,423
KSHV-MIR-K12-10B	6,43E-01	-1,187	-0,248
MIR-545	6,45E-01	1,706	0,770
MIR-374B-374C	6,46E-01	1,638	0,712
MIR-335	6,48E-01	1,471	0,556
MIR-582-5P	6,48E-01	1,662	0,733
MIR-193A-3P	6,50E-01	1,369	0,453
MIR-1292	6,50E-01	-1,298	-0,376
MIR-633	6,51E-01	1,755	0,811
MIR-513A-5P	6,51E-01	-1,513	-0,597
MIR-1910	6,52E-01	-1,586	-0,665
MIR-592	6,52E-01	1,315	0,395
MIR-578	6,55E-01	1,648	0,721
MIR-1304	6,55E-01	-1,205	-0,270
MIR-338-3P	6,56E-01	1,656	0,727
MIR-636	6,58E-01	-1,261	-0,334
MIR-138	6,58E-01	-1,274	-0,350
MIR-138-2*	6,58E-01	-1,286	-0,363
MIR-624	6,60E-01	1,481	0,567
MIR-556-3P	6,61E-01	-1,265	-0,339
MIR-374B*-374C*	6,64E-01	-1,232	-0,301
MIR-100	6,64E-01	-1,286	-0,362
MIR-1244	6,67E-01	-1,203	-0,266
MIR-9	6,69E-01	1,838	0,878
MIR-1972	6,69E-01	-1,530	-0,613
MIR-562	6,70E-01	1,599	0,677
MIR-320C	6,70E-01	-1,270	-0,345
MIR-380-3P	6,71E-01	1,714	0,777
MIR-337-3P	6,71E-01	1,777	0,829
MIR-548C-5P	6,72E-01	1,682	0,750
MIR-612	6,72E-01	-1,239	-0,309
MIR-106B*	6,73E-01	-1,856	-0,892
MIR-603	6,74E-01	1,699	0,765

MIR-144*	6,76E-01	-1,253	-0,326
MIR-320D	6,77E-01	-1,274	-0,349
MIR-137	6,79E-01	1,689	0,756
MIR-2054	6,82E-01	-1,247	-0,318
MIR-518B	6,84E-01	-1,249	-0,321
MIR-556-5P	6,84E-01	-1,348	-0,431
MIR-218-1*	6,85E-01	-1,316	-0,397
MIR-490-3P	6,86E-01	-1,314	-0,394
MIR-32	6,87E-01	1,706	0,770
MIR-450A-5P	6,90E-01	1,595	0,673
MIR-670	6,91E-01	1,358	0,441
MIR-548J	6,94E-01	-1,281	-0,358
MIR-1265	6,94E-01	-1,394	-0,479
MIR-138-1*	6,95E-01	-1,573	-0,654
MIR-513A-3P	6,96E-01	1,589	0,668
MIR-1207-3P	6,96E-01	-1,334	-0,416
MIR-29B-1*	6,96E-01	-1,258	-0,331
MIR-765	6,96E-01	-1,355	-0,438
MIR-590-3P	6,96E-01	-1,350	-0,433
MIR-34A*	6,98E-01	-1,272	-0,347
MIR-302B	6,98E-01	1,563	0,644
MIR-204	6,99E-01	1,266	0,341
MIR-499-5P	6,99E-01	1,687	0,754
MIR-585	7,01E-01	1,330	0,411
MIR-665	7,02E-01	-1,290	-0,367
MIR-1233	7,02E-01	-1,479	-0,565
MIR-615-3P	7,02E-01	-1,204	-0,268
MIR-345-5P	7,03E-01	-1,227	-0,296
MIR-586	7,04E-01	1,551	0,633
MIR-135B*	7,05E-01	1,919	0,940
HSV1-MIR-H2	7,06E-01	-1,597	-0,675
MIR-19B-1*	7,08E-01	-1,234	-0,304
MIR-1913	7,09E-01	-1,453	-0,539
MIR-1470	7,09E-01	-1,488	-0,573
MIR-580	7,10E-01	1,747	0,805
MIR-412	7,12E-01	-1,210	-0,276
MIR-134	7,14E-01	-1,201	-0,264
MIR-661	7,14E-01	-1,286	-0,362
EBV-MIR-BART3-5P	7,14E-01	1,259	0,332
LET-7E*	7,14E-01	-1,359	-0,443
JCV-MIR-J1-3P-BKV-MIR-B1-3P	7,20E-01	-1,180	-0,238
EBV-MIR-BART10	7,21E-01	-1,187	-0,248
MIR-1305	7,22E-01	-1,292	-0,369
MIR-338-5P	7,23E-01	-1,190	-0,251
MIR-29B	7,24E-01	1,209	0,274
MIR-581	7,24E-01	1,687	0,755
MIR-590-5P	7,26E-01	1,789	0,839
MIR-501-5P	7,26E-01	-1,165	-0,220
MIR-27B	7,27E-01	-1,245	-0,316
HSV1-MIR-H7*	7,27E-01	-1,480	-0,565
MIR-524-5P	7,29E-01	1,769	0,823
MIR-376A	7,30E-01	1,293	0,370
MIR-520A-5P	7,32E-01	1,185	0,245
MIR-18B	7,37E-01	-1,231	-0,300
MIR-718	7,37E-01	-1,195	-0,257
MIR-498	7,38E-01	-1,210	-0,275
MIR-302C	7,42E-01	1,301	0,380
MIR-515-5P	7,44E-01	1,550	0,633
MIR-186*	7,44E-01	-1,163	-0,217

MIR-2276	7,45E-01	-1,351	-0,434
MIR-199B-5P	7,48E-01	1,309	0,388
EBV-MIR-BART17-5P	7,49E-01	-1,219	-0,286
HCMV-MIR-UL70-3P	7,49E-01	-1,213	-0,279
MIR-523	7,49E-01	-1,221	-0,288
HCMV-MIR-US5-1	7,51E-01	-1,211	-0,276
MIR-496_2	7,52E-01	1,281	0,357
MIR-520A-3P	7,52E-01	1,171	0,228
MIR-559	7,52E-01	1,586	0,666
MIR-1283	7,55E-01	-1,254	-0,326
MIR-16	7,56E-01	1,136	0,184
MIR-361-5P	7,57E-01	-1,184	-0,244
MIR-7-2*	7,57E-01	-1,210	-0,274
MIR-140-3P	7,57E-01	-1,180	-0,238
MIR-148A*	7,59E-01	-1,301	-0,379
MIR-532-5P	7,59E-01	-1,310	-0,390
MIR-720	7,64E-01	-1,207	-0,271
MIR-1207-5P	7,64E-01	-1,259	-0,332
MIR-146B-5P	7,65E-01	-1,176	-0,234
MIR-193A-5P	7,66E-01	-1,400	-0,486
MIR-548L	7,68E-01	-1,548	-0,630
MIR-616	7,69E-01	-1,231	-0,300
EBV-MIR-BHRF1-1	7,69E-01	-1,159	-0,213
HCMV-MIR-US33-3P	7,70E-01	-1,237	-0,307
MIR-223*	7,71E-01	-1,151	-0,203
MIR-377*	7,71E-01	-1,276	-0,352
MIR-1258	7,71E-01	-1,197	-0,259
LET-7G*	7,71E-01	-1,298	-0,376
MIR-1324	7,71E-01	-1,236	-0,306
MIR-185*	7,72E-01	-1,263	-0,336
MIR-200B	7,72E-01	-1,170	-0,227
MIR-648	7,72E-01	-1,301	-0,380
MIR-924	7,72E-01	-1,192	-0,254
HCMV-MIR-US4	7,73E-01	-1,184	-0,243
MIR-519B-3P	7,73E-01	1,395	0,480
MIR-190	7,73E-01	-1,271	-0,346
MIR-664-3P	7,74E-01	-1,308	-0,388
EBV-MIR-BART16	7,74E-01	-1,215	-0,280
MIR-100*	7,75E-01	-1,174	-0,231
MIR-614	7,76E-01	-1,328	-0,409
MIR-490-5P	7,82E-01	-1,224	-0,292
MIR-551B	7,82E-01	1,253	0,326
MIR-652	7,82E-01	1,235	0,305
MIR-922	7,82E-01	-1,264	-0,338
MIR-602	7,84E-01	-1,251	-0,323
MIR-1278	7,84E-01	-1,254	-0,327
MIR-593*	7,85E-01	-1,248	-0,320
MIR-198	7,86E-01	-1,225	-0,293
MIR-621	7,87E-01	-1,298	-0,376
MIR-889	7,87E-01	1,474	0,560
MIR-550A	7,88E-01	-1,179	-0,238
MIR-668	7,89E-01	-1,177	-0,236
MIR-521	7,90E-01	1,272	0,347
MIR-874	7,91E-01	-1,250	-0,321
MIR-513B	7,92E-01	1,597	0,675
MIR-520C-3P-520F-520B_4	7,94E-01	1,384	0,468
MIR-329	7,96E-01	1,177	0,235
MIR-1249	7,96E-01	-1,303	-0,382
MIR-518A-5P-527_2	7,96E-01	-1,202	-0,265

MIR-145*	8,01E-01	-1,199	-0,262
MIR-769-3P	8,02E-01	-1,208	-0,272
EBV-MIR-BHRF1-3	8,04E-01	-1,187	-0,247
MIR-576-3P	8,06E-01	1,311	0,390
MIR-432	8,06E-01	-1,168	-0,224
MIR-941	8,06E-01	1,207	0,272
EBV-MIR-BART13	8,08E-01	-1,210	-0,275
MIR-574-5P	8,09E-01	-1,254	-0,327
MIR-526B*	8,09E-01	1,313	0,393
MIR-1247	8,10E-01	-1,454	-0,540
MIR-26B*	8,11E-01	-1,158	-0,212
MIR-615-5P	8,12E-01	-1,182	-0,241
MIR-141*	8,13E-01	-1,135	-0,183
MIR-410	8,13E-01	1,240	0,310
MIR-608	8,14E-01	-1,182	-0,242
LET-7F-2*	8,15E-01	-1,214	-0,279
MIR-183	8,18E-01	-1,212	-0,277
MIR-215	8,19E-01	1,211	0,276
EBV-MIR-BART19-3P	8,19E-01	1,463	0,549
MIR-936	8,20E-01	-1,134	-0,182
MIR-1A	8,21E-01	1,557	0,639
EBV-MIR-BART7	8,21E-01	-1,173	-0,230
MIR-200B*	8,21E-01	-1,296	-0,374
MIR-99A*	8,23E-01	-1,267	-0,341
HSV1-MIR-H8*	8,24E-01	-1,261	-0,335
MIR-214	8,24E-01	-1,178	-0,236
MIR-653	8,25E-01	1,414	0,500
MIR-525-3P	8,26E-01	1,158	0,211
MIR-218	8,26E-01	1,418	0,503
MIR-124	8,27E-01	-1,245	-0,316
MIR-658	8,28E-01	-1,228	-0,296
MIR-95	8,29E-01	1,312	0,391
KSHV-MIR-K12-2	8,30E-01	-1,181	-0,240
MIR-146A*	8,30E-01	-1,260	-0,333
MIR-491-5P	8,30E-01	-1,238	-0,308
MIR-432*	8,30E-01	-1,235	-0,304
MIR-331-5P	8,33E-01	-1,164	-0,220
MIR-365	8,34E-01	1,545	0,628
KSHV-MIR-K12-12*	8,34E-01	-1,161	-0,216
MIR-605	8,36E-01	-1,202	-0,266
MIR-1915*	8,37E-01	-1,448	-0,535
MIR-151-5P-151B	8,39E-01	-1,164	-0,220
EBV-MIR-BART11-3P	8,39E-01	-1,183	-0,242
MIR-1303	8,39E-01	-1,177	-0,235
MIR-1286	8,39E-01	1,256	0,329
HCMV-MIR-US5-2	8,40E-01	-1,161	-0,216
MIR-384-5P	8,40E-01	1,260	0,333
MIR-495	8,41E-01	1,216	0,282
EBV-MIR-BART18-3P	8,41E-01	-1,169	-0,225
MIR-2110	8,44E-01	-1,222	-0,289
MIR-770-3P	8,45E-01	-1,189	-0,250
SV40-MIR-S1-5P	8,46E-01	-1,190	-0,251
MIR-101-101C	8,47E-01	1,337	0,420
MIR-2116*	8,47E-01	-1,349	-0,432
MIR-548D-5P	8,47E-01	-1,263	-0,337
EBV-MIR-BART3-3P	8,47E-01	-1,154	-0,207
MIR-600	8,48E-01	-1,163	-0,218
MIR-1288	8,48E-01	1,489	0,575
MIR-211	8,52E-01	1,188	0,249

MIR-639	8,53E-01	-1,269	-0,343
MIR-147A	8,54E-01	1,210	0,275
KSHV-MIR-K12-3	8,54E-01	-1,204	-0,268
MIR-597	8,55E-01	-1,208	-0,273
MCV-MIR-M1-3P	8,56E-01	-1,251	-0,324
MIR-93	8,58E-01	-1,168	-0,224
MIR-19B	8,59E-01	-1,263	-0,337
MIR-629	8,59E-01	-1,150	-0,201
HCMV-MIR-UL70-5P	8,60E-01	-1,243	-0,314
MIR-340-3P	8,62E-01	1,208	0,273
KSHV-MIR-K12-1	8,63E-01	-1,157	-0,210
MIR-362-5P	8,63E-01	-1,161	-0,216
MIR-186	8,63E-01	1,219	0,286
MIR-591	8,65E-01	1,235	0,305
MIR-1306-3P	8,65E-01	-1,153	-0,206
MIR-106B	8,66E-01	-1,207	-0,272
MIR-136	8,68E-01	1,341	0,424
MIR-450B-5P	8,68E-01	1,376	0,461
MIR-129*	8,69E-01	1,529	0,613
MIR-30C-1*	8,69E-01	-1,180	-0,239
MIR-940	8,71E-01	-1,231	-0,300
MIR-588	8,71E-01	-1,180	-0,239
MIR-214*	8,71E-01	-1,233	-0,302
MIR-193B	8,72E-01	-1,150	-0,201
MIR-542-5P	8,72E-01	-1,159	-0,213
MIR-877*	8,73E-01	-1,221	-0,288
MIR-34C-3P	8,76E-01	-1,233	-0,302
MIR-1243	8,76E-01	-1,104	-0,143
MIR-606	8,77E-01	-1,286	-0,363
MIR-126-5P	8,77E-01	-1,197	-0,259
MIR-1224-3P	8,78E-01	-1,225	-0,292
MIR-96*	8,78E-01	1,224	0,291
MIR-632	8,78E-01	1,201	0,264
MIR-144	8,79E-01	1,452	0,538
MIR-29C	8,79E-01	-1,156	-0,209
MIR-483-3P	8,80E-01	-1,138	-0,186
MIR-26A	8,80E-01	1,107	0,146
MIR-298	8,80E-01	-1,157	-0,210
EBV-MIR-BART20-5P_3	8,81E-01	1,146	0,196
EBV-MIR-BART9*	8,81E-01	-1,370	-0,455
MIR-769-5P	8,81E-01	-1,175	-0,233
MIR-1263	8,82E-01	-1,251	-0,323
MIR-363	8,82E-01	1,143	0,193
MIR-760-3P	8,82E-01	-1,137	-0,185
MIR-637	8,85E-01	-1,165	-0,220
MIR-508-5P	8,86E-01	-1,200	-0,263
MIR-147B	8,86E-01	1,179	0,237
MIR-129-5P	8,86E-01	-1,152	-0,204
MIR-384-3P	8,87E-01	1,166	0,221
MIR-607	8,88E-01	1,407	0,493
EBV-MIR-BART13*	8,88E-01	-1,370	-0,454
MIR-296-3P	8,89E-01	-1,165	-0,220
MIR-210	8,91E-01	-1,237	-0,307
MIR-433-3P	8,92E-01	-1,136	-0,184
MIR-1227	8,92E-01	-1,149	-0,201
MIR-647	8,93E-01	-1,192	-0,253
MIR-488*	8,93E-01	1,183	0,243
KSHV-MIR-K12-5	8,94E-01	-1,149	-0,201
MIR-27A*	8,94E-01	-1,135	-0,183

MIR-1180	8,97E-01	-1,215	-0,281
MIR-224	8,97E-01	1,142	0,192
EBV-MIR-BART14-5P	8,97E-01	1,181	0,240
MIR-767-5P	8,97E-01	-1,161	-0,216
MIR-885-5P	8,97E-01	-1,134	-0,181
MIR-1276	8,97E-01	1,250	0,322
MIR-541-3P	8,98E-01	-1,209	-0,274
MIR-1271	8,98E-01	-1,356	-0,439
MIR-2113	8,99E-01	-1,111	-0,151
MIR-10A	8,99E-01	1,138	0,187
HCMV-MIR-US25-2-5P	9,00E-01	-1,147	-0,198
MIR-202-5P	9,00E-01	-1,135	-0,183
MIR-19A*	9,01E-01	-1,130	-0,176
MIR-503	9,03E-01	1,196	0,258
MIR-509-5P	9,03E-01	-1,150	-0,202
MIR-1280	9,03E-01	-1,304	-0,383
MIR-708	9,04E-01	-1,169	-0,225
MIR-31	9,04E-01	-1,142	-0,191
MIR-103A	9,04E-01	-1,098	-0,134
MIR-517A-MIR-517B_1	9,05E-01	1,166	0,222
MIR-34C-5P	9,05E-01	-1,204	-0,268
MIR-1277	9,05E-01	1,319	0,399
MIR-367*	9,05E-01	-1,167	-0,223
MIR-659	9,06E-01	1,107	0,146
MIR-644	9,06E-01	1,247	0,318
MIR-2114	9,06E-01	-1,262	-0,335
MIR-575	9,06E-01	-1,152	-0,205
MIR-744*	9,06E-01	-1,141	-0,191
MIR-1471	9,07E-01	-1,325	-0,406
MIR-322-MIR-424	9,07E-01	-1,330	-0,411
MIR-609	9,08E-01	1,251	0,323
MIR-601	9,09E-01	-1,124	-0,168
MIR-572	9,09E-01	-1,138	-0,186
HCMV-MIR-US25-2-3P	9,10E-01	-1,155	-0,208
MIR-449B	9,10E-01	-1,127	-0,172
MIR-136*	9,12E-01	-1,153	-0,205
KSHV-MIR-K12-9	9,13E-01	1,143	0,192
MIR-761	9,14E-01	-1,156	-0,209
MIR-548C-3P	9,15E-01	-1,138	-0,186
MIR-566	9,16E-01	-1,164	-0,219
MIR-449A	9,16E-01	1,120	0,164
MIR-431*	9,16E-01	-1,163	-0,218
EBV-MIR-BART6-5P	9,16E-01	-1,168	-0,224
EBV-MIR-BART11-5P	9,17E-01	-1,114	-0,156
MIR-30C	9,19E-01	-1,107	-0,147
MIR-26B	9,20E-01	1,195	0,258
HSV1-MIR-H2*	9,20E-01	-1,294	-0,372
MIR-2117	9,21E-01	-1,146	-0,196
MIR-28-3P	9,21E-01	-1,159	-0,213
KSHV-MIR-K12-7	9,22E-01	-1,134	-0,182
MIR-512-3P	9,22E-01	-1,119	-0,162
MIR-526B	9,23E-01	1,152	0,204
MIR-500A*	9,23E-01	-1,103	-0,141
MIR-26A-1*	9,24E-01	-1,169	-0,225
MIR-361-3P	9,25E-01	-1,286	-0,363
MIR-518C	9,25E-01	1,203	0,267
MIR-655	9,25E-01	1,180	0,239
HSV1-MIR-H3	9,26E-01	-1,185	-0,244
MIR-518C*	9,26E-01	-1,100	-0,138

MIR-671-5P	9,26E-01	-1,145	-0,195
MIR-1914*	9,27E-01	-1,131	-0,177
EBV-MIR-BART6-3P	9,27E-01	-1,153	-0,206
MIR-885-3P	9,28E-01	-1,119	-0,162
MIR-502-3P	9,28E-01	-1,099	-0,136
MIR-620	9,28E-01	1,331	0,412
MIR-125B-1*	9,28E-01	-1,147	-0,198
MIR-1284	9,28E-01	-1,231	-0,300
MIR-921	9,29E-01	-1,250	-0,322
MIR-208A	9,29E-01	1,177	0,235
MIR-106A*	9,30E-01	1,394	0,480
EBV-MIR-BART10*	9,30E-01	-1,240	-0,310
MIR-873	9,31E-01	-1,147	-0,198
MIR-363*	9,32E-01	-1,125	-0,170
MIR-27A	9,33E-01	-1,106	-0,145
MIR-502-5P	9,33E-01	1,107	0,147
MIR-29B-2*	9,34E-01	1,121	0,164
MIR-671-3P	9,34E-01	-1,109	-0,150
MIR-512-5P	9,35E-01	-1,121	-0,165
LET-7C	9,35E-01	-1,088	-0,122
MIR-98	9,36E-01	1,224	0,292
MIR-346	9,37E-01	1,117	0,159
EBV-MIR-BART20-3P	9,38E-01	-1,146	-0,197
MIR-143*	9,38E-01	-1,171	-0,228
MIR-576-5P	9,38E-01	-1,160	-0,214
MIR-577	9,39E-01	-1,181	-0,240
MIR-331-3P	9,39E-01	1,131	0,178
MIR-630	9,39E-01	-1,106	-0,145
MIR-221*	9,39E-01	-1,274	-0,350
MIR-216A	9,40E-01	1,144	0,194
MIR-19B-2*	9,41E-01	-1,077	-0,108
MIR-654-3P	9,42E-01	1,146	0,196
MIR-30D	9,42E-01	1,105	0,144
MIR-7-1*	9,42E-01	1,156	0,209
MIR-219-2-3P	9,42E-01	-1,174	-0,232
MIR-23A	9,42E-01	-1,123	-0,167
MIR-548Q	9,42E-01	-1,166	-0,222
MIR-643	9,43E-01	1,104	0,143
MIR-618	9,43E-01	1,285	0,362
MIR-518D-5P-520C-5P-526A	9,43E-01	-1,104	-0,142
MIR-1236	9,43E-01	-1,144	-0,194
MIR-20B*	9,43E-01	-1,112	-0,153
MIR-2115	9,46E-01	-1,192	-0,253
MIR-532-3P	9,49E-01	-1,091	-0,125
MIR-194	9,49E-01	1,138	0,186
MIR-599	9,49E-01	1,201	0,264
MIR-625	9,50E-01	-1,101	-0,138
MIR-557	9,50E-01	-1,096	-0,132
EBV-MIR-BART15	9,50E-01	1,113	0,154
MIR-431	9,51E-01	-1,116	-0,159
MIR-561	9,51E-01	1,283	0,360
MIR-151-3P	9,52E-01	1,115	0,157
MIR-10A*	9,53E-01	-1,200	-0,263
SV40-MIR-S1-3P	9,54E-01	1,108	0,148
MIR-492	9,54E-01	-1,106	-0,145
MIR-589	9,54E-01	-1,174	-0,232
MIR-101*	9,54E-01	-1,105	-0,144
MIR-124*	9,54E-01	-1,180	-0,239
MIR-24-1*	9,55E-01	1,138	0,187

MIR-139-3P	9,56E-01	1,334	0,416
MIR-330-5P	9,56E-01	-1,086	-0,119
MIR-1255A	9,56E-01	-1,097	-0,134
MIR-887	9,56E-01	-1,122	-0,166
KSHV-MIR-K12-8	9,56E-01	-1,133	-0,180
MIR-888*	9,57E-01	1,085	0,118
MIR-519D	9,57E-01	1,143	0,193
KSHV-MIR-K12-6-5P	9,57E-01	-1,088	-0,122
LET-7I*	9,57E-01	-1,089	-0,123
HSV2-MIR-H4-5P	9,57E-01	-1,163	-0,218
MIR-485-5P	9,58E-01	-1,100	-0,137
MIR-299-3P	9,58E-01	-1,100	-0,138
MIR-146B-3P	9,58E-01	1,191	0,252
MIR-125B	9,58E-01	-1,142	-0,191
MIR-1539	9,58E-01	-1,226	-0,293
MIR-1237	9,59E-01	-1,163	-0,218
MIR-383	9,59E-01	-1,082	-0,113
HIV1-MIR-H1	9,59E-01	-1,107	-0,146
MIR-764	9,59E-01	1,239	0,309
HCMV-MIR-UL148D	9,59E-01	-1,102	-0,139
MIR-30D*	9,59E-01	-1,161	-0,216
MIR-323B-5P	9,59E-01	-1,087	-0,121
MIR-1285	9,59E-01	-1,157	-0,210
MIR-372	9,60E-01	-1,099	-0,136
EBV-MIR-BHRF1-2*	9,60E-01	1,174	0,231
MIR-497	9,60E-01	-1,107	-0,146
MIR-126-3P	9,60E-01	1,144	0,194
MIR-589*	9,60E-01	-1,119	-0,163
MIR-99A	9,61E-01	-1,154	-0,206
MIR-199A-5P	9,61E-01	-1,103	-0,142
MIR-1295	9,61E-01	-1,097	-0,134
MIR-2277-3P	9,62E-01	-1,122	-0,166
MIR-489	9,62E-01	-1,095	-0,130
MIR-17	9,62E-01	1,097	0,134
MIR-519E*	9,63E-01	1,101	0,139
MIR-452	9,63E-01	1,098	0,135
MIR-181B	9,63E-01	1,106	0,146
MIR-1827	9,63E-01	-1,214	-0,280
MIR-122	9,63E-01	-1,077	-0,107
MIR-544	9,63E-01	1,110	0,151
MIR-23B	9,64E-01	-1,085	-0,118
MIR-484	9,64E-01	-1,092	-0,128
MIR-196A*	9,64E-01	-1,186	-0,246
MIR-369-5P	9,65E-01	1,104	0,143
MIR-892B	9,65E-01	-1,094	-0,129
MIR-200C	9,66E-01	-1,089	-0,123
MIR-219-5P	9,67E-01	-1,139	-0,187
MIR-646	9,67E-01	-1,117	-0,159
LET-7A*-LET-7C-2*	9,68E-01	-1,094	-0,129
MIR-493*	9,68E-01	1,239	0,310
MIR-411	9,69E-01	1,089	0,124
EBV-MIR-BART2-3P	9,69E-01	-1,080	-0,111
MIR-573	9,69E-01	-1,109	-0,149
MIR-1184	9,69E-01	-1,211	-0,276
MIR-711	9,69E-01	-1,098	-0,135
MIR-148B*	9,70E-01	-1,207	-0,272
MIR-1909*	9,70E-01	-1,175	-0,233
MIR-330-3P	9,70E-01	-1,075	-0,105
MIR-216B	9,70E-01	1,112	0,153

MIR-222*	9,70E-01	1,161	0,216
MIR-518F*	9,71E-01	-1,086	-0,119
MIR-195	9,71E-01	1,115	0,157
MIR-1205	9,71E-01	-1,126	-0,171
MIR-29A*	9,71E-01	1,124	0,169
MIR-339-3P	9,72E-01	-1,085	-0,118
MIR-509-3-5P	9,72E-01	-1,067	-0,093
MIR-622	9,72E-01	-1,089	-0,123
KSHV-MIR-K12-11	9,73E-01	1,094	0,129
MIR-181A*	9,73E-01	-1,106	-0,145
MIR-1322	9,73E-01	1,239	0,309
MIR-891A	9,73E-01	-1,101	-0,139
MIR-564	9,74E-01	-1,094	-0,129
MIR-31*	9,74E-01	-1,098	-0,135
MIR-107	9,74E-01	-1,054	-0,076
MIR-206	9,75E-01	-1,139	-0,188
MIR-192	9,76E-01	1,096	0,132
MIR-548A-3P	9,76E-01	1,167	0,223
EBV-MIR-BART9	9,76E-01	-1,069	-0,096
MIR-325-5P	9,76E-01	1,096	0,132
MIR-1185	9,76E-01	1,058	0,082
MIR-202-3P	9,76E-01	-1,082	-0,113
MIR-619	9,77E-01	-1,121	-0,165
MIR-623	9,77E-01	-1,081	-0,112
MIR-524-3P	9,77E-01	-1,082	-0,114
MIR-149	9,77E-01	-1,083	-0,115
MIR-1307	9,78E-01	-1,182	-0,241
MIR-375	9,78E-01	-1,084	-0,116
MIR-18A*	9,78E-01	1,089	0,122
EBV-MIR-BART1-3P	9,79E-01	1,082	0,114
MIR-635	9,79E-01	-1,108	-0,148
MIR-187	9,80E-01	-1,067	-0,093
MIR-188-3P	9,80E-01	-1,120	-0,163
MIR-139-5P	9,80E-01	-1,088	-0,121
MIR-15A*	9,80E-01	-1,193	-0,255
MIR-1181	9,81E-01	-1,147	-0,198
MIR-553	9,81E-01	1,147	0,198
MIR-567	9,81E-01	-1,113	-0,155
MIR-649	9,82E-01	1,090	0,125
MIR-550A*	9,82E-01	-1,070	-0,097
MIR-628-3P	9,82E-01	-1,056	-0,079
MIR-449C	9,82E-01	1,181	0,240
MIR-500A-500B	9,83E-01	1,078	0,109
MIR-125A-5P	9,83E-01	1,084	0,117
MIR-938	9,83E-01	-1,076	-0,105
MIR-520G	9,83E-01	1,076	0,105
MIR-302D*	9,83E-01	-1,131	-0,177
MIR-34B-5P	9,83E-01	1,074	0,103
MIR-548N	9,83E-01	1,069	0,096
MIR-132*	9,84E-01	1,177	0,235
KSHV-MIR-K12-10A	9,84E-01	-1,057	-0,080
MIR-21*	9,84E-01	1,227	0,296
MIR-549	9,84E-01	1,058	0,082
LET-7D*	9,84E-01	1,130	0,177
MIR-548B-5P	9,84E-01	-1,078	-0,108
MIR-142-5P	9,84E-01	-1,068	-0,095
MIR-890	9,84E-01	1,078	0,108
MIR-2278	9,84E-01	-1,108	-0,148
MIR-181A-2*	9,85E-01	-1,062	-0,087

MIR-485-3P	9,85E-01	-1,055	-0,077
MIR-181D	9,85E-01	-1,064	-0,089
MIR-17*	9,85E-01	1,075	0,104
MIR-140-5P	9,85E-01	-1,084	-0,116
MIR-340-5P	9,85E-01	1,113	0,154
MIR-510	9,85E-01	-1,077	-0,107
MIR-660	9,85E-01	-1,087	-0,121
MIR-143	9,85E-01	-1,075	-0,105
MIR-1203	9,86E-01	1,170	0,227
MIR-541-5P	9,86E-01	-1,082	-0,114
MIR-25	9,87E-01	-1,082	-0,113
MIR-299-5P	9,87E-01	1,075	0,104
EBV-MIR-BART1-5P	9,87E-01	-1,054	-0,076
MIR-582-3P	9,87E-01	1,077	0,106
MIR-770-5P	9,87E-01	-1,089	-0,123
MIR-323-3P	9,87E-01	-1,062	-0,086
MIR-650	9,88E-01	-1,056	-0,079
MIR-194*	9,88E-01	-1,146	-0,197
MIR-604	9,88E-01	-1,071	-0,099
MIR-181C	9,88E-01	-1,070	-0,097
MIR-371-3P	9,89E-01	-1,061	-0,085
MIR-641	9,89E-01	-1,063	-0,089
MIR-1281	9,89E-01	-1,122	-0,166
MIR-22	9,89E-01	-1,086	-0,119
MIR-369-3P	9,89E-01	-1,062	-0,087
MIR-105	9,89E-01	-1,068	-0,094
MIR-513C	9,90E-01	-1,071	-0,099
MIR-30A*	9,90E-01	-1,063	-0,088
MIR-150	9,91E-01	1,059	0,083
MIR-379	9,91E-01	-1,057	-0,080
MIR-1254	9,91E-01	1,106	0,145
MIR-200C*	9,91E-01	1,117	0,160
MIR-487A	9,91E-01	-1,049	-0,069
MIR-1825	9,91E-01	-1,118	-0,161
EBV-MIR-BART8-3P	9,91E-01	-1,068	-0,095
MIR-373	9,91E-01	-1,063	-0,088
MIR-1238	9,92E-01	1,118	0,161
MIR-300-3P	9,92E-01	-1,051	-0,071
MIR-99B	9,92E-01	-1,055	-0,078
HCMV-MIR-US25-1	9,93E-01	-1,061	-0,086
MIR-379*	9,93E-01	-1,115	-0,157
MIR-516A-5P	9,93E-01	-1,056	-0,079
MIR-875-3P	9,93E-01	-1,059	-0,083
MIR-30C-2*	9,93E-01	-1,090	-0,125
MIR-342-5P	9,93E-01	-1,178	-0,236
HBV-MIR-B20	9,93E-01	1,054	0,076
MIR-122*	9,93E-01	-1,076	-0,105
HIV1-MIR-N367	9,93E-01	-1,059	-0,082
LET-7A-2*	9,93E-01	-1,078	-0,108
MIR-130A	9,94E-01	1,060	0,084
MIR-548I	9,94E-01	1,074	0,103
MIR-409-3P	9,94E-01	-1,049	-0,068
MIR-629*	9,94E-01	-1,071	-0,099
HSV1-MIR-H7	9,94E-01	-1,065	-0,091
MIR-583	9,94E-01	-1,079	-0,110
MIR-598-3P	9,94E-01	1,064	0,089
MIR-297A	9,94E-01	1,049	0,069
MIR-551B*	9,94E-01	-1,061	-0,085
JCV-MIR-J1-5P	9,95E-01	-1,045	-0,064

EBV-MIR-BART7*	9,95E-01	-1,098	-0,135
MIR-197	9,95E-01	-1,068	-0,095
MIR-509-3P	9,95E-01	-1,046	-0,065
EBV-MIR-BART14-3P	9,95E-01	-1,044	-0,062
MIR-127	9,96E-01	-1,037	-0,052
MIR-587	9,96E-01	1,052	0,073
MIR-1226	9,96E-01	-1,137	-0,185
MIR-217	9,96E-01	1,038	0,053
MIR-1272	9,96E-01	-1,047	-0,066
KSHV-MIR-K12-4-3P	9,96E-01	-1,046	-0,064
MIR-16-1*	9,96E-01	-1,083	-0,115
MIR-455-3P	9,96E-01	-1,045	-0,064
MIR-33*	9,96E-01	-1,041	-0,058
MIR-1204	9,96E-01	1,120	0,163
MIR-196B*	9,96E-01	1,122	0,166
HCMV-MIR-UL36*	9,96E-01	1,074	0,104
MIR-548D-3P	9,96E-01	1,079	0,110
EBV-MIR-BART19-5P	9,96E-01	1,045	0,063
MIR-449C*	9,96E-01	-1,072	-0,100
MIR-409-5P	9,97E-01	1,045	0,064
MIR-610	9,97E-01	-1,039	-0,055
MIR-30B*	9,97E-01	-1,066	-0,092
MIR-504	9,97E-01	1,038	0,054
EBV-MIR-BART21-3P	9,97E-01	-1,054	-0,076
MIR-423-5P	9,97E-01	-1,069	-0,096
KSHV-MIR-K12-6-3P	9,97E-01	1,032	0,045
LET-7B*	9,97E-01	-1,108	-0,148
MIR-584	9,97E-01	-1,034	-0,049
MIR-105*	9,97E-01	-1,065	-0,092
LET-7G	9,97E-01	1,031	0,044
MCV-MIR-M1-5P	9,97E-01	-1,058	-0,082
MIR-190B	9,97E-01	1,054	0,075
MIR-411*	9,98E-01	-1,036	-0,051
MIR-30A	9,98E-01	1,043	0,061
MIR-381	9,98E-01	-1,038	-0,053
EBV-MIR-BART4*	9,98E-01	-1,085	-0,118
MIR-1250	9,98E-01	1,081	0,113
MIR-651	9,98E-01	1,066	0,092
MIR-1269	9,98E-01	-1,063	-0,088
MIR-181C*	9,98E-01	-1,044	-0,063
MIR-29C*	9,98E-01	1,034	0,048
MIR-486-5P	9,98E-01	-1,030	-0,043
MIR-1202	9,98E-01	-1,099	-0,137
MIR-920	9,98E-01	-1,060	-0,084
EBV-MIR-BART4	9,98E-01	-1,041	-0,058
MIR-1200	9,98E-01	-1,074	-0,103
MIR-645	9,98E-01	-1,038	-0,054
MIR-184	9,98E-01	-1,035	-0,049
MIR-374A	9,98E-01	1,068	0,095
MIR-421-5P	9,98E-01	1,080	0,111
MIR-448	9,98E-01	-1,027	-0,039
MIR-141	9,98E-01	1,041	0,058
LET-7I	9,98E-01	1,032	0,045
MIR-337-5P	9,98E-01	-1,036	-0,051
MIR-1226*	9,98E-01	-1,052	-0,073
MIR-486-3P	9,98E-01	1,074	0,103
MIR-152	9,98E-01	1,031	0,044
MIR-518E	9,99E-01	-1,029	-0,042
MIR-767-3P	9,99E-01	-1,031	-0,044

MIR-455-5P	9,99E-01	1,030	0,043
MIR-617	9,99E-01	-1,032	-0,046
MIR-205	9,99E-01	1,039	0,055
MIR-517*	9,99E-01	-1,026	-0,037
MIR-555	9,99E-01	-1,027	-0,039
MIR-944	9,99E-01	-1,031	-0,044
MIR-362-3P	9,99E-01	-1,051	-0,072
MIR-2116	9,99E-01	-1,037	-0,052
MIR-33B	9,99E-01	-1,066	-0,093
MIR-182_1	9,99E-01	-1,026	-0,037
MIR-675*	9,99E-01	-1,065	-0,091
MIR-301A	9,99E-01	-1,036	-0,050
MIR-125B-2*	9,99E-01	-1,068	-0,095
MIR-1298	9,99E-01	1,027	0,038
MIR-18A	9,99E-01	1,036	0,051
HSV2-MIR-H2	9,99E-01	1,065	0,091
MIR-571	9,99E-01	-1,026	-0,036
LET-7B	9,99E-01	1,028	0,040
KSHV-MIR-K12-9*	9,99E-01	-1,032	-0,046
MIR-224*	9,99E-01	1,061	0,085
MIR-1245	9,99E-01	1,038	0,054
KSHV-MIR-K12-4-5P	9,99E-01	-1,028	-0,040
MIR-133A	9,99E-01	1,029	0,042
MIR-181A	9,99E-01	1,037	0,053
MIR-324-5P	9,99E-01	-1,024	-0,034
MIR-133B	9,99E-01	1,028	0,040
HBV-MIR-B2RC	9,99E-01	-1,027	-0,039
MIR-626	9,99E-01	1,046	0,064
MIR-1976	9,99E-01	-1,043	-0,061
MIR-520D-3P	9,99E-01	1,019	0,027
MIR-1182	9,99E-01	-1,033	-0,047
MIR-634	1,00E+00	1,050	0,070
MIR-525-5P	1,00E+00	-1,018	-0,025
MIR-302C*	1,00E+00	-1,019	-0,027
MIR-520D-5P	1,00E+00	-1,020	-0,029
MIR-506	1,00E+00	-1,027	-0,039
EBV-MIR-BART5	1,00E+00	1,026	0,038
BKV-MIR-B1-5P	1,00E+00	1,034	0,049
MIR-543-3P	1,00E+00	-1,019	-0,026
MIR-1229	1,00E+00	1,040	0,057
MIR-187*	1,00E+00	1,048	0,068
MIR-99B*	1,00E+00	1,035	0,050
MIR-154	1,00E+00	1,027	0,039
MIR-551A	1,00E+00	-1,019	-0,027
MIR-1266	1,00E+00	1,048	0,067
HSV2-MIR-H3	1,00E+00	-1,015	-0,022
HSV2-MIR-H4-3P	1,00E+00	-1,046	-0,065
MIR-18B*	1,00E+00	1,048	0,067
MIR-200A	1,00E+00	1,027	0,038
MIR-191	1,00E+00	-1,016	-0,022
MIR-15A	1,00E+00	-1,022	-0,031
MIR-518D-3P	1,00E+00	1,015	0,022
MIR-501-3P	1,00E+00	-1,040	-0,057
MIR-218-2*	1,00E+00	1,024	0,034
MIR-574-3P	1,00E+00	-1,017	-0,025
MIR-758	1,00E+00	-1,019	-0,027
MIR-656	1,00E+00	1,026	0,038
EBV-MIR-BART5*	1,00E+00	1,042	0,059
HSV1-MIR-H8	1,00E+00	-1,023	-0,033

MIR-382	1,00E+00	1,011	0,016
MIR-425*	1,00E+00	1,019	0,027
MIR-518A-3P	1,00E+00	1,014	0,020
MIR-515-3P	1,00E+00	1,015	0,021
MIR-1291	1,00E+00	-1,025	-0,036
MIR-516B	1,00E+00	-1,012	-0,017
MIR-365B	1,00E+00	1,016	0,022
LET-7D	1,00E+00	-1,009	-0,014
MIR-103B	1,00E+00	1,024	0,034
MIR-195*	1,00E+00	1,027	0,039
HCMV-MIR-UL36	1,00E+00	-1,011	-0,015
MIR-145	1,00E+00	1,012	0,017
MIR-28-5P-28C	1,00E+00	-1,017	-0,024
MIR-106A	1,00E+00	1,008	0,011
MIR-92B*	1,00E+00	-1,012	-0,018
MIR-30E	1,00E+00	-1,012	-0,017
MIR-627	1,00E+00	1,014	0,020
MIR-1225-3P	1,00E+00	1,019	0,028
MIR-23B*	1,00E+00	1,016	0,023
MIR-1911*	1,00E+00	-1,023	-0,033
MIR-30B	1,00E+00	1,013	0,019
MIR-192*	1,00E+00	-1,020	-0,028
MIR-2114*	1,00E+00	-1,013	-0,018
MIR-92A	1,00E+00	1,012	0,017
HCMV-MIR-UL22A	1,00E+00	1,007	0,010
MIR-302E	1,00E+00	-1,014	-0,020
MIR-24	1,00E+00	1,006	0,008
MIR-188-5P	1,00E+00	-1,008	-0,012
MIR-657	1,00E+00	1,008	0,012
MIR-449B*	1,00E+00	1,013	0,018
MIR-129-3P	1,00E+00	1,008	0,011
HBV-MIR-B4	1,00E+00	1,008	0,011
HSV1-MIR-H6-3P	1,00E+00	1,012	0,018
MIR-200A*	1,00E+00	-1,007	-0,010
MIR-322*-MIR-424*	1,00E+00	-1,007	-0,010
MIR-1261	1,00E+00	-1,006	-0,009
MIR-518F	1,00E+00	1,008	0,011
MIR-493	1,00E+00	1,004	0,006
MIR-16-2*	1,00E+00	-1,006	-0,009
MIR-103A-2*	1,00E+00	-1,006	-0,009
MIR-2115*	1,00E+00	1,004	0,005
MIR-20A*	1,00E+00	1,005	0,007
MIR-24-2*	1,00E+00	-1,007	-0,011
MIR-505*	1,00E+00	-1,006	-0,008
MIR-21	1,00E+00	-1,001	-0,002
KSHV-MIR-K12-3*	1,00E+00	-1,001	-0,001
MIR-191*	1,00E+00	1,000	0,000
