# Supplementary material for: Effector and regulatory dendritic cells display distinct patterns of miRNA expression
Source: Immun Inflamm Dis. 2017 May 12;5(3):310–7. doi: 10.1002/iid3.165 (PMC5569363; doi:10.1002/iid3.165)
Supplement: Supplementary file 5 — DCreg. Microarray results DCreg vs Unstimulated DCs [file IID3-5-310-s005.pdf]

	DCreg vs Unstimulated DC		
**miRNA name**	**Tukey.p-value**	**Fold_Change**	**Log2_Ratio**
MIR-762	4,82E-07	2,639	1,400
MIR-33A	2,27E-06	2,452	1,294
MIR-142-5P	1,28E-05	-2,394	-1,260
MIR-520A-3P	2,85E-05	2,068	1,048
MIR-663	3,15E-05	2,425	1,278
MIR-508-3P	3,73E-05	2,495	1,319
MIR-525-3P	4,12E-05	2,154	1,107
MIR-659	4,36E-05	1,945	0,960
MIR-363	4,78E-05	2,233	1,159
MIR-616*	5,13E-05	2,266	1,180
KSHV-MIR-K12-6-3P	1,40E-04	1,944	0,959
MIR-302A	1,42E-04	2,942	1,557
MIR-382	2,28E-04	1,805	0,852
MIR-448	2,36E-04	1,902	0,928
MIR-643	2,62E-04	2,040	1,028
MIR-1185	2,80E-04	1,753	0,810
MIR-520A-5P	2,82E-04	1,951	0,964
MIR-135A	3,12E-04	2,720	1,444
MIR-549	3,21E-04	1,878	0,909
MIR-888*	3,77E-04	1,889	0,917
MIR-449A	4,99E-04	1,998	0,999
MIR-329	5,66E-04	1,995	0,997
MIR-628-3P	6,19E-04	1,763	0,818
MIR-759	7,07E-04	2,719	1,443
EBV-MIR-BART20-5P_3	7,18E-04	2,020	1,015
MIR-10A	7,44E-04	2,035	1,025
MIR-203	7,52E-04	2,159	1,110
EBV-MIR-BART2-5P	9,27E-04	2,935	1,554
HBV-MIR-B20	9,77E-04	2,076	1,054
MIR-204	9,78E-04	2,262	1,178
MIR-224	1,04E-03	2,024	1,017
MIR-127	1,06E-03	1,774	0,827
MIR-592	1,14E-03	2,423	1,277
MIR-642A	1,14E-03	2,148	1,103
MIR-122	1,30E-03	1,759	0,815
KSHV-MIR-K12-6-5P	1,55E-03	1,828	0,870
MIR-509-3-5P	1,65E-03	1,723	0,785
HCMV-MIR-UL22A	1,66E-03	1,793	0,843
MIR-504	1,90E-03	1,903	0,928
MIR-152	1,91E-03	1,960	0,971
MIR-502-5P	1,91E-03	1,838	0,878
EBV-MIR-BART3-5P	1,93E-03	2,173	1,120
MIR-154	1,95E-03	2,611	1,385
MIR-211	2,03E-03	2,139	1,097
HCMV-MIR-UL36	2,13E-03	1,789	0,840
MIR-487A	2,20E-03	1,770	0,824
EBV-MIR-BART1-5P	2,30E-03	1,729	0,790
MIR-328-5P	2,35E-03	4,292	2,102
MIR-196A	2,41E-03	2,528	1,338
MIR-187	2,59E-03	1,782	0,834
MIR-217	2,64E-03	1,749	0,807
MIR-585	2,66E-03	2,526	1,337
MIR-10B	2,73E-03	2,139	1,097
MIR-383	2,73E-03	1,732	0,792
MIR-495	3,03E-03	2,259	1,176
MIR-613	3,16E-03	2,932	1,552
HBV-MIR-B2RC	3,40E-03	2,168	1,117

MIR-455-5P	3,52E-03	1,940	0,956
SV40-MIR-S1-3P	3,57E-03	1,963	0,973
MIR-29C*	3,83E-03	1,866	0,900
MIR-539-5P	3,84E-03	2,663	1,413
HBV-MIR-B4	3,84E-03	2,201	1,138
MIR-301B	3,88E-03	2,582	1,369
MIR-302D	3,89E-03	2,265	1,180
MIR-215	3,90E-03	2,100	1,070
MIR-496_2	4,04E-03	2,320	1,214
MIR-337-3P	4,04E-03	2,183	1,126
MIR-550A*	4,13E-03	1,823	0,866
MIR-888	4,20E-03	2,452	1,294
MIR-153	4,24E-03	2,573	1,363
MIR-30E	4,35E-03	-2,006	-1,004
MIR-96	4,44E-03	3,097	1,631
MIR-2115*	4,58E-03	2,041	1,029
MIR-302C	4,59E-03	2,382	1,252
MIR-199A-3P-199B-3P	4,59E-03	2,415	1,272
MIR-670	4,65E-03	2,524	1,336
MIR-302A*	4,71E-03	2,370	1,245
MIR-410	4,91E-03	2,240	1,163
MIR-376A	4,96E-03	2,279	1,188
MIR-520G	4,96E-03	1,933	0,951
MIR-302C*	4,96E-03	1,754	0,811
MIR-330-3P	4,97E-03	1,711	0,775
MIR-186	5,12E-03	2,324	1,217
MIR-299-5P	5,39E-03	2,016	1,012
MIR-429	5,49E-03	3,128	1,645
MIR-409-3P	5,56E-03	1,837	0,877
MIR-369-5P	5,70E-03	1,984	0,989
MIR-182*	5,72E-03	2,120	1,084
MIR-516B	6,10E-03	1,740	0,799
MIR-411	6,20E-03	1,843	0,882
MIR-610	6,30E-03	1,814	0,859
MIR-650	6,32E-03	1,713	0,777
MIR-520D-3P	6,36E-03	1,720	0,783
MIR-432	6,44E-03	1,755	0,812
MIR-584	6,51E-03	1,729	0,790
MIR-488*	6,68E-03	2,157	1,109
MIR-452	6,92E-03	1,871	0,904
MIR-181B	7,11E-03	1,966	0,975
MIR-26B	7,14E-03	-2,466	-1,302
EBV-MIR-BART7	7,22E-03	1,803	0,850
MIR-2117	7,31E-03	1,987	0,991
MIR-517A-MIR-517B_1	7,48E-03	2,062	1,044
MIR-501-5P	7,49E-03	1,600	0,678
MIR-892A	7,51E-03	2,745	1,457
MIR-148A	7,66E-03	2,882	1,527
MIR-34B-5P	7,97E-03	1,864	0,898
MIR-199B-5P	8,06E-03	2,341	1,227
MIR-1275	8,29E-03	4,053	2,019
MIR-200B	8,41E-03	1,679	0,748
MIR-485-3P	8,50E-03	1,603	0,681
MIR-1909	9,08E-03	3,906	1,966
MIR-22*	9,22E-03	2,236	1,161
MIR-516A-3P-MIR-516*	9,33E-03	2,784	1,477
MIR-624*	9,38E-03	2,190	1,131
MIR-505	9,38E-03	1,838	0,878
MIR-452*	9,40E-03	2,287	1,194

HSV2-MIR-H3	9,51E-03	1,670	0,740
EBV-MIR-BART1-3P	9,63E-03	1,863	0,898
MIR-632	1,01E-02	2,142	1,099
MIR-192	1,09E-02	1,965	0,974
MIR-323-3P	1,11E-02	1,743	0,802
EBV-MIR-BART11-5P	1,11E-02	1,674	0,743
MIR-325-3P	1,11E-02	2,277	1,187
MIR-140-5P	1,12E-02	-2,017	-1,012
KSHV-MIR-K12-11	1,15E-02	1,886	0,915
MIR-371-3P	1,15E-02	1,763	0,818
MIR-421-3P	1,21E-02	1,499	0,584
MIR-340-3P	1,22E-02	2,090	1,063
MIR-376B	1,24E-02	2,826	1,499
MIR-325-5P	1,25E-02	1,959	0,970
MIR-630	1,30E-02	1,697	0,763
MIR-30A	1,32E-02	1,956	0,968
MIR-518D-3P	1,34E-02	1,733	0,793
MIR-518C	1,34E-02	2,455	1,296
MIR-520E	1,37E-02	2,669	1,416
MIR-601	1,40E-02	1,693	0,760
MIR-513C	1,43E-02	1,947	0,961
MIR-154*	1,44E-02	2,484	1,313
MIR-300-3P	1,45E-02	1,694	0,761
MIR-506	1,47E-02	2,094	1,066
MIR-767-3P	1,47E-02	1,791	0,840
MIR-625	1,48E-02	1,705	0,770
MIR-526B	1,48E-02	1,964	0,974
MIR-323B-5P	1,49E-02	1,651	0,724
EBV-MIR-BART2-3P	1,50E-02	1,658	0,729
MIR-377	1,51E-02	3,025	1,597
MIR-623	1,52E-02	1,761	0,816
MIR-216A	1,52E-02	2,017	1,012
MIR-518A-3P	1,52E-02	1,805	0,852
MIR-551A	1,55E-02	1,767	0,822
MIR-9*	1,57E-02	2,752	1,460
MIR-521	1,58E-02	2,155	1,108
MIR-454*	1,65E-02	3,281	1,714
MIR-1228*	1,66E-02	2,960	1,566
MIR-518C*	1,68E-02	1,578	0,658
MIR-649	1,68E-02	1,963	0,973
MIR-302B*	1,69E-02	2,955	1,563
MIR-548N	1,70E-02	1,711	0,775
MIR-543-3P	1,70E-02	1,664	0,734
MIR-367	1,75E-02	3,050	1,609
MIR-297A	1,75E-02	1,747	0,805
MIR-451	1,76E-02	3,263	1,706
MIR-517*	1,82E-02	1,655	0,727
MIR-891B	1,87E-02	2,682	1,424
MIR-454_2	1,87E-02	2,756	1,462
MIR-1286	1,87E-02	2,222	1,152
MIR-491-3P	1,88E-02	2,995	1,583
MIR-376C	1,88E-02	2,361	1,240
MIR-380-3P	1,89E-02	3,997	1,999
MIR-558	1,89E-02	2,803	1,487
MIR-511	1,92E-02	2,971	1,571
MIR-555	1,92E-02	1,701	0,767
MIR-141*	1,93E-02	1,517	0,601
EBV-MIR-BART9	1,95E-02	1,594	0,673
MIR-147A	2,00E-02	1,985	0,989

MIR-557	2,01E-02	1,638	0,712
MIR-579	2,02E-02	3,311	1,727
HCMV-MIR-US25-1*	2,08E-02	1,735	0,795
MIR-708*	2,09E-02	2,523	1,335
MIR-130A	2,11E-02	1,893	0,921
MIR-202-3P	2,15E-02	1,727	0,788
MIR-381	2,16E-02	1,757	0,813
MIR-379	2,18E-02	1,691	0,757
MIR-485-5P	2,18E-02	1,710	0,774
MIR-548B-3P	2,19E-02	3,121	1,642
MIR-483-3P	2,19E-02	1,645	0,718
MIR-335	2,22E-02	2,571	1,362
HCMV-MIR-UL22A*	2,23E-02	3,026	1,598
KSHV-MIR-K12-4-3P	2,23E-02	1,761	0,816
MIR-372	2,25E-02	1,712	0,776
MIR-384-3P	2,27E-02	1,826	0,869
MIR-409-5P	2,29E-02	1,828	0,870
MIR-184	2,34E-02	1,735	0,795
MIR-196B	2,37E-02	2,253	1,172
MIR-194	2,38E-02	1,967	0,976
MIR-665	2,40E-02	1,947	0,961
MIR-145	2,45E-02	1,721	0,783
MIR-151-3P	2,45E-02	1,785	0,836
MIR-486-5P	2,46E-02	1,604	0,681
KSHV-MIR-K12-4-5P	2,46E-02	1,885	0,915
EBV-MIR-BART8-5P	2,47E-02	2,961	1,566
MIR-890	2,48E-02	1,804	0,851
MIR-519E*	2,48E-02	1,751	0,809
MIR-542-3P	2,58E-02	3,919	1,971
MIR-199A-5P	2,58E-02	1,754	0,810
MIR-638	2,58E-02	2,022	1,016
MIR-99B	2,58E-02	1,716	0,779
EBV-MIR-BART19-5P	2,64E-02	1,764	0,819
MIR-551B	2,70E-02	1,951	0,964
MIR-411*	2,70E-02	1,679	0,748
MIR-671-3P	2,73E-02	1,629	0,704
MIR-147B	2,75E-02	1,875	0,907
MIR-340-5P	2,75E-02	2,324	1,217
MIR-1227	2,76E-02	1,721	0,783
MIR-624	2,80E-02	2,590	1,373
MIR-519A	2,80E-02	3,321	1,731
EBV-MIR-BART19-3P	2,81E-02	3,348	1,743
MIR-618	2,81E-02	3,432	1,779
MIR-522	2,83E-02	2,787	1,479
MIR-622	2,84E-02	1,719	0,782
MIR-493	2,97E-02	1,775	0,827
MIR-18A*	2,97E-02	1,792	0,842
KSHV-MIR-K12-9	3,01E-02	1,747	0,805
MIR-609	3,02E-02	2,510	1,328
MIR-324-5P	3,04E-02	1,703	0,768
MIR-130B	3,06E-02	1,495	0,580
MIR-1243	3,17E-02	1,433	0,519
MIR-2116	3,25E-02	1,966	0,975
MIR-582-5P	3,28E-02	3,279	1,713
KSHV-MIR-K12-10A	3,40E-02	1,514	0,599
MIR-525-5P	3,40E-02	1,549	0,632
MIR-591	3,43E-02	2,084	1,059
MIR-182_1	3,44E-02	1,612	0,689
MIR-181D	3,48E-02	1,605	0,683

MIR-576-3P	3,50E-02	2,256	1,174
EBV-MIR-BART10	3,51E-02	1,556	0,638
MIR-519B-3P	3,53E-02	2,547	1,349
MIR-195	3,55E-02	1,940	0,956
MIR-512-3P	3,56E-02	1,614	0,690
MIR-135B	3,57E-02	3,363	1,750
MIR-208A	3,63E-02	2,053	1,038
KSHV-MIR-K12-3*	3,65E-02	1,694	0,760
MIR-517C	3,68E-02	2,857	1,514
KSHV-MIR-K12-7	3,69E-02	1,703	0,768
MIR-212	3,78E-02	1,706	0,771
EBV-MIR-BART14-3P	3,78E-02	1,629	0,704
KSHV-MIR-K12-10B	3,82E-02	1,479	0,564
MIR-711	3,82E-02	1,733	0,793
MIR-668	3,83E-02	1,597	0,675
MCV-MIR-M1-5P	3,86E-02	2,173	1,120
MIR-876-5P	3,89E-02	3,161	1,661
MIR-146B-3P	3,92E-02	2,521	1,334
MIR-412	3,92E-02	1,614	0,691
MIR-433-3P	3,92E-02	1,607	0,684
MIR-562	3,93E-02	3,012	1,591
MIR-509-3P	3,98E-02	1,645	0,718
MIR-569	4,02E-02	3,622	1,857
MIR-514	4,04E-02	3,575	1,838
MIR-524-3P	4,06E-02	1,669	0,739
MIR-376A*	4,16E-02	3,173	1,666
MIR-200C	4,17E-02	1,610	0,687
MIR-95	4,18E-02	2,311	1,209
MIR-515-3P	4,19E-02	1,694	0,761
MIR-563	4,22E-02	2,985	1,578
MIR-1298	4,25E-02	1,641	0,714
EBV-MIR-BART18-5P	4,26E-02	2,815	1,493
MIR-519E	4,29E-02	2,838	1,505
MIR-369-3P	4,39E-02	1,647	0,720
MIR-141	4,50E-02	1,868	0,902
MIR-578	4,52E-02	3,084	1,625
MIR-30A*	4,54E-02	1,688	0,755
MIR-363*	4,57E-02	1,663	0,734
MIR-629*	4,64E-02	2,001	1,001
MIR-545	4,67E-02	3,265	1,707
MIR-330-5P	4,71E-02	1,514	0,599
HCMV-MIR-US5-2	4,72E-02	1,593	0,672
MIR-2277-3P	4,75E-02	1,837	0,878
MIR-519D	4,80E-02	1,963	0,973
MIR-586	4,85E-02	2,865	1,518
JCV-MIR-J1-5P	4,86E-02	1,587	0,666
MIR-574-3P	4,87E-02	1,750	0,808
MIR-550A	4,87E-02	1,579	0,659
MIR-181A-2*	4,89E-02	1,548	0,630
MIR-553	4,89E-02	2,502	1,323
MIR-629	4,90E-02	1,578	0,658
EBV-MIR-BART21-3P	4,95E-02	1,952	0,965
MIR-2113	4,98E-02	1,474	0,560
MIR-449B	5,15E-02	1,584	0,663
KSHV-MIR-K12-3	5,17E-02	1,812	0,857
MIR-142-3P	5,20E-02	-2,441	-1,288
MIR-526B*	5,33E-02	2,173	1,120
MIR-598-3P	5,35E-02	1,866	0,900
MIR-655	5,40E-02	1,964	0,974

MIR-488	5,46E-02	2,622	1,391
MIR-603	5,48E-02	3,299	1,722
MIR-137	5,65E-02	3,268	1,708
MIR-491-5P	5,67E-02	1,884	0,914
MIR-520D-5P	5,68E-02	1,579	0,659
MIR-7	5,83E-02	3,365	1,751
MIR-518E	5,83E-02	1,576	0,656
MIR-298	5,83E-02	1,636	0,710
MIR-548C-5P	5,88E-02	3,169	1,664
HCMV-MIR-US4	5,89E-02	1,555	0,637
MIR-1260	5,92E-02	-2,620	-1,389
MIR-885-5P	5,92E-02	1,570	0,651
MIR-181C	5,94E-02	1,686	0,754
MIR-136	5,98E-02	2,592	1,374
MIR-149*	6,02E-02	2,227	1,155
MIR-571	6,02E-02	1,604	0,681
EBV-MIR-BHRF1-1	6,06E-02	1,465	0,551
KSHV-MIR-K12-12*	6,14E-02	1,555	0,637
MIR-633	6,18E-02	3,335	1,738
MIR-17*	6,39E-02	1,657	0,728
MIR-302B	6,44E-02	2,765	1,467
KSHV-MIR-K12-2	6,51E-02	1,618	0,695
MIR-206	6,52E-02	2,158	1,110
MIR-455-3P	6,52E-02	1,635	0,709
MIR-299-3P	6,55E-02	1,597	0,675
MIR-507	6,55E-02	2,831	1,501
MIR-891A	6,57E-02	1,728	0,789
MIR-766	6,61E-02	1,834	0,875
MIR-219-5P	6,66E-02	1,990	0,993
MIR-1293	6,67E-02	1,507	0,591
MIR-654-3P	6,71E-02	1,798	0,847
MIR-641	6,72E-02	1,607	0,685
MIR-497	6,78E-02	1,650	0,722
MIR-346	6,78E-02	1,587	0,666
MIR-572	6,82E-02	1,606	0,684
MIR-450A-5P	6,93E-02	2,820	1,496
MIR-645	6,94E-02	1,685	0,752
MIR-150	6,95E-02	1,599	0,677
MIR-570	6,96E-02	2,746	1,457
MIR-573	6,97E-02	1,748	0,806
MIR-520H	7,02E-02	2,938	1,555
EBV-MIR-BART15	7,03E-02	1,624	0,700
MIR-1908	7,17E-02	1,892	0,920
MIR-876-3P	7,19E-02	3,029	1,599
MIR-200A	7,20E-02	2,109	1,077
MIR-760-3P	7,34E-02	1,523	0,607
MIR-599	7,36E-02	2,269	1,182
MIR-559	7,38E-02	3,069	1,618
KSHV-MIR-K12-9*	7,39E-02	1,808	0,854
EBV-MIR-BART17-3P	7,42E-02	1,507	0,591
MIR-938	7,43E-02	1,610	0,687
MIR-216B	7,45E-02	1,771	0,825
MIR-365B	7,51E-02	1,697	0,763
MIR-520C-3P-520F-520B_4	7,53E-02	2,340	1,227
MIR-7-1*	7,54E-02	1,852	0,889
EBV-MIR-BART6-3P	7,54E-02	1,745	0,803
MIR-513B	7,59E-02	3,381	1,757
MIR-657	7,59E-02	1,705	0,770
LET-7E	7,61E-02	-1,395	-0,481

MIR-617	7,65E-02	1,652	0,724
MIR-29B-1*	7,67E-02	1,660	0,731
MIR-587	7,74E-02	1,688	0,755
MIR-1207-5P	7,79E-02	1,760	0,816
MIR-542-5P	7,92E-02	1,589	0,668
KSHV-MIR-K12-5	7,92E-02	1,599	0,677
MIR-519C-3P	7,97E-02	3,135	1,649
MIR-129-3P	8,04E-02	1,725	0,786
MIR-9	8,07E-02	3,593	1,845
MIR-371-5P	8,12E-02	1,744	0,802
MIR-523	8,29E-02	1,605	0,683
MIR-23A*	8,43E-02	1,426	0,512
MIR-516A-5P	8,48E-02	1,596	0,674
HCMV-MIR-UL148D	8,51E-02	1,579	0,659
MIR-604	8,55E-02	1,647	0,720
MIR-589*	8,58E-02	1,710	0,774
MIR-513A-3P	8,62E-02	2,717	1,442
MIR-331-5P	8,68E-02	1,525	0,609
MIR-548L	8,70E-02	2,891	1,532
MIR-515-5P	8,73E-02	2,773	1,472
MIR-489	8,75E-02	1,545	0,627
MIR-875-3P	8,93E-02	1,631	0,706
MIR-1276	9,01E-02	2,108	1,076
LET-7D*	9,01E-02	2,205	1,141
EBV-MIR-BHRF1-2	9,05E-02	3,542	1,825
MIR-431	9,07E-02	1,621	0,697
MIR-510	9,08E-02	1,632	0,706
MIR-600	9,09E-02	1,541	0,624
HCMV-MIR-US25-2-5P	9,12E-02	1,587	0,667
MIR-644	9,21E-02	2,131	1,092
MIR-339-3P	9,26E-02	1,542	0,625
MIR-218	9,45E-02	2,560	1,356
MIR-802	9,49E-02	3,418	1,773
MIR-222*	9,62E-02	2,166	1,115
MIR-612	9,68E-02	1,551	0,633
MIR-373*	9,70E-02	1,455	0,541
MIR-143	9,97E-02	1,610	0,687
LET-7I*	1,00E-01	1,463	0,549
EBV-MIR-BART17-5P	1,01E-01	1,574	0,654
MIR-337-3P	1,02E-01	3,195	1,676
HCMV-MIR-UL112	1,02E-01	1,480	0,566
MIR-320A	1,05E-01	1,528	0,611
MIR-718	1,06E-01	1,485	0,571
MIR-181A*	1,06E-01	1,695	0,761
MIR-10B*	1,07E-01	1,380	0,465
MIR-944	1,07E-01	1,598	0,676
MIR-662	1,09E-01	1,441	0,527
MIR-1179	1,12E-01	1,407	0,493
HIV1-MIR-TAR-5P	1,13E-01	1,429	0,515
MIR-375	1,13E-01	1,564	0,646
HSV1-MIR-H8	1,13E-01	1,938	0,954
EBV-MIR-BART8-3P	1,13E-01	1,658	0,729
EBV-MIR-BART14-5P	1,14E-01	1,701	0,767
MIR-16-2*	1,14E-01	2,158	1,109
MIR-518B	1,15E-01	1,564	0,645
MIR-23B	1,15E-01	-1,467	-0,553
MIR-581	1,15E-01	3,061	1,614
MIR-337-5P	1,18E-01	1,606	0,683
MIR-126-5P	1,18E-01	1,702	0,767

MIR-384-5P	1,18E-01	1,854	0,891
MIR-499-5P	1,19E-01	2,914	1,543
MIR-24-1*	1,19E-01	1,740	0,799
MIR-105	1,19E-01	1,585	0,664
MIR-892B	1,20E-01	1,520	0,604
MIR-616	1,22E-01	1,605	0,683
MIR-1290	1,22E-01	1,363	0,447
HCMV-MIR-UL36*	1,22E-01	2,062	1,044
EBV-MIR-BART4	1,24E-01	1,654	0,726
MIR-615-3P	1,24E-01	1,459	0,545
MIR-518D-5P-520C-5P-526A	1,25E-01	1,468	0,554
MIR-508-5P	1,26E-01	1,732	0,792
MIR-764	1,26E-01	2,557	1,355
MIR-653	1,26E-01	2,416	1,273
MIR-133A	1,27E-01	1,659	0,731
MIR-32	1,28E-01	2,874	1,523
MIR-545*	1,28E-01	1,454	0,540
MIR-582-3P	1,29E-01	1,617	0,694
KSHV-MIR-K12-1	1,29E-01	1,501	0,586
EBV-MIR-BHRF1-3	1,30E-01	1,512	0,596
MIR-608	1,31E-01	1,510	0,594
MIR-149	1,31E-01	1,534	0,617
HCMV-MIR-US5-1	1,33E-01	1,515	0,600
MIR-374B-374C	1,34E-01	2,491	1,317
MIR-761	1,34E-01	1,612	0,689
MIR-640	1,35E-01	1,507	0,592
MIR-654-5P	1,35E-01	1,481	0,566
MIR-31	1,36E-01	1,520	0,604
MIR-208B	1,39E-01	2,506	1,325
HSV1-MIR-H4*	1,40E-01	1,367	0,451
MIR-1288	1,41E-01	2,856	1,514
MIR-370	1,42E-01	1,461	0,547
MIR-1261	1,43E-01	1,671	0,741
MIR-1246	1,43E-01	2,220	1,150
MIR-338-3P	1,44E-01	2,535	1,342
MIR-129-5P	1,46E-01	1,510	0,595
MIR-126-3P	1,47E-01	1,779	0,831
MIR-33*	1,48E-01	1,469	0,554
MIR-151-5P-151B	1,49E-01	1,472	0,558
HSV1-MIR-H7	1,50E-01	1,680	0,748
SV40-MIR-S1-5P	1,51E-01	1,564	0,645
MIR-518E*-519A*-1-519B-5P-519C-5P-522*-523*-526C	1,51E-01	1,386	0,471
MIR-1322	1,51E-01	2,838	1,505
MIR-373	1,51E-01	1,560	0,642
MIR-2276	1,52E-01	1,875	0,907
EBV-MIR-BART11-3P	1,53E-01	1,527	0,611
MIR-627	1,54E-01	1,723	0,785
MIR-205	1,57E-01	1,705	0,770
MIR-1244	1,59E-01	1,402	0,487
MIR-449C*	1,60E-01	1,940	0,956
MIR-223*	1,61E-01	1,355	0,438
MIR-590-5P	1,61E-01	3,196	1,676
MIR-885-3P	1,62E-01	1,462	0,548
HIV1-MIR-N367	1,63E-01	1,552	0,634
MIR-1469	1,63E-01	2,236	1,161
MIR-155	1,64E-01	1,772	0,825
MIR-1277	1,65E-01	2,317	1,212
MIR-615-5P	1,66E-01	1,475	0,560
MIR-875-5P	1,67E-01	2,672	1,418

LET-7F	1,68E-01	-1,345	-0,428
MIR-588	1,71E-01	1,560	0,641
MIR-134	1,72E-01	1,425	0,511
MIR-634	1,72E-01	2,432	1,282
MIR-889	1,73E-01	2,336	1,224
MIR-1254	1,74E-01	1,971	0,979
MIR-106A*	1,79E-01	3,038	1,603
MIR-2054	1,79E-01	1,494	0,579
MIR-492	1,79E-01	1,476	0,562
EBV-MIR-BART18-3P	1,83E-01	1,461	0,547
MIR-2053	1,84E-01	1,406	0,492
MIR-190B	1,84E-01	1,721	0,784
MIR-133B	1,85E-01	1,600	0,678
MIR-1289	1,86E-01	1,458	0,544
MIR-200A*	1,86E-01	1,647	0,720
MIR-2278	1,88E-01	1,774	0,827
MIR-580	1,88E-01	2,846	1,509
MIR-31*	1,89E-01	1,548	0,631
MIR-101-101C	1,90E-01	2,036	1,026
MIR-144	1,90E-01	2,715	1,441
HCMV-MIR-US25-2-3P	1,90E-01	1,537	0,620
MIR-564	1,90E-01	1,516	0,600
MIR-652	1,94E-01	1,561	0,642
MIR-770-3P	1,95E-01	1,522	0,606
MIR-197	1,96E-01	1,710	0,774
MIR-26A-1*	1,97E-01	1,638	0,712
MIR-191*	1,98E-01	1,607	0,684
MIR-100*	2,00E-01	1,392	0,477
MIR-2114*	2,01E-01	1,804	0,851
EBV-MIR-BART21-5P	2,01E-01	1,389	0,474
MIR-21	2,03E-01	-1,401	-0,487
MIR-193B	2,04E-01	1,431	0,517
MIR-651	2,04E-01	1,988	0,991
MIR-548A-3P	2,04E-01	2,068	1,048
MIR-182_2	2,04E-01	1,434	0,520
MIR-596	2,06E-01	1,403	0,489
MIR-296-3P	2,06E-01	1,513	0,598
MIR-379*	2,07E-01	2,153	1,107
MIR-1304	2,08E-01	1,371	0,455
MIR-513A-5P	2,10E-01	1,999	0,999
MIR-1321	2,10E-01	1,409	0,495
MIR-205*	2,11E-01	1,359	0,443
MIR-450B-5P	2,12E-01	2,238	1,162
MIR-1303	2,15E-01	1,458	0,544
MIR-20A	2,15E-01	-1,623	-0,698
MIR-93*	2,15E-01	1,378	0,462
MIR-767-5P	2,16E-01	1,511	0,595
MIR-656	2,17E-01	1,895	0,922
MIR-675	2,17E-01	1,460	0,545
MIR-2114	2,19E-01	1,931	0,949
MIR-874	2,22E-01	1,584	0,664
MIR-518A-5P-527_2	2,22E-01	1,469	0,555
MIR-92A-2*	2,23E-01	1,576	0,656
MIR-125A-5P	2,23E-01	1,519	0,603
HCMV-MIR-US25-1	2,23E-01	1,504	0,589
MIR-129*	2,26E-01	2,867	1,520
MIR-637	2,27E-01	1,488	0,573
MIR-186*	2,29E-01	1,328	0,409
MIR-575	2,31E-01	1,487	0,573

MIR-374A	2,33E-01	2,039	1,028
MIR-96*	2,36E-01	1,664	0,735
MIR-887	2,37E-01	1,524	0,608
MIR-544	2,38E-01	1,505	0,590
MIR-214	2,40E-01	1,427	0,513
MIR-30B*	2,41E-01	1,784	0,835
MIR-541-3P	2,42E-01	1,661	0,732
MIR-188-3P	2,42E-01	1,716	0,779
JCV-MIR-J1-3P-BKV-MIR-B1-3P	2,43E-01	1,341	0,424
MIR-181C*	2,45E-01	1,561	0,642
MIR-34B-3P	2,45E-01	2,418	1,274
EBV-MIR-BART22	2,45E-01	1,365	0,449
MIR-19A*	2,47E-01	1,389	0,474
MIR-934	2,48E-01	1,298	0,376
MIR-2052	2,48E-01	1,368	0,452
MIR-301A	2,49E-01	1,571	0,652
MIR-607	2,50E-01	2,397	1,261
MIR-1256	2,53E-01	1,329	0,411
EBV-MIR-BART12	2,53E-01	1,429	0,515
MIR-139-5P	2,53E-01	1,492	0,577
MIR-646	2,54E-01	1,551	0,633
MIR-548A-5P	2,54E-01	1,317	0,398
EBV-MIR-BART13	2,55E-01	1,482	0,567
MIR-502-3P	2,56E-01	1,328	0,409
MIR-769-3P	2,57E-01	1,466	0,552
MIR-362-5P	2,58E-01	1,419	0,505
MIR-188-5P	2,66E-01	1,510	0,595
MIR-626	2,68E-01	1,999	0,999
MIR-27B*	2,68E-01	1,263	0,337
MIR-1291	2,70E-01	1,869	0,903
EBV-MIR-BART5	2,71E-01	1,547	0,629
HCMV-MIR-US33-5P	2,73E-01	1,346	0,428
MIR-323-5P	2,75E-01	1,376	0,461
HCMV-MIR-UL70-3P	2,78E-01	1,413	0,499
MIR-498	2,80E-01	1,398	0,484
MIR-532-3P	2,80E-01	1,331	0,412
MIR-595	2,80E-01	1,394	0,480
MIR-664-5P	2,81E-01	1,476	0,562
MIR-324-3P	2,81E-01	1,394	0,479
MIR-548P-548AM	2,83E-01	1,410	0,496
MIR-29B-2*	2,85E-01	1,405	0,491
MIR-541-5P	2,86E-01	1,509	0,594
MIR-877*	2,86E-01	1,593	0,671
MIR-365	2,88E-01	2,490	1,316
MIR-500A-500B	2,89E-01	1,432	0,518
LET-7B	2,90E-01	1,447	0,533
MIR-519C-5P	2,90E-01	1,364	0,448
MIR-497*	2,93E-01	1,301	0,380
MIR-509-5P	2,94E-01	1,433	0,519
HIV1-MIR-H1	3,01E-01	1,423	0,509
MIR-143*	3,02E-01	1,607	0,684
MIR-939	3,04E-01	1,390	0,475
MIR-25	3,06E-01	1,495	0,581
MIR-635	3,07E-01	1,570	0,651
MIR-101*	3,07E-01	1,396	0,482
MIR-769-5P	3,07E-01	1,461	0,547
MIR-605	3,09E-01	1,463	0,549
MIR-548I	3,10E-01	1,602	0,680
MIR-183	3,10E-01	1,461	0,547

MIR-320B	3,11E-01	1,382	0,467
MIR-708	3,15E-01	1,478	0,564
MIR-620	3,16E-01	2,226	1,155
MIR-589	3,18E-01	1,694	0,760
MIR-1279	3,18E-01	1,299	0,377
MIR-548B-5P	3,19E-01	1,427	0,513
MIR-770-5P	3,21E-01	1,542	0,625
MIR-518F*	3,22E-01	1,372	0,456
MIR-548C-3P	3,23E-01	1,401	0,486
EBV-MIR-BART6-5P	3,27E-01	1,501	0,586
MIR-873	3,27E-01	1,472	0,558
MIR-30C-1*	3,29E-01	1,440	0,526
MIR-500A*	3,32E-01	1,302	0,381
MIR-486-3P	3,34E-01	2,075	1,053
MIR-1182	3,34E-01	1,624	0,700
MIR-103B	3,37E-01	1,802	0,850
MIR-29B	3,38E-01	-1,356	-0,440
MIR-1248	3,39E-01	1,347	0,430
MIR-548F-MIR-548G	3,41E-01	1,338	0,420
MIR-103A-2*	3,45E-01	1,790	0,840
EBV-MIR-BART16	3,50E-01	1,399	0,484
MIR-130B*	3,53E-01	1,274	0,349
MIR-18B*	3,53E-01	2,364	1,241
HSV1-MIR-H4	3,55E-01	1,284	0,360
MIR-181A	3,56E-01	1,637	0,711
MIR-218-2*	3,65E-01	1,607	0,685
KSHV-MIR-K12-8	3,65E-01	1,486	0,571
MIR-374A*	3,66E-01	1,372	0,456
MIR-16-1*	3,71E-01	1,777	0,830
MIR-125A-3P	3,72E-01	1,743	0,802
MIR-548D-3P	3,73E-01	1,752	0,809
MIR-1973	3,75E-01	2,766	1,468
MIR-561	3,75E-01	2,124	1,087
MIR-1255A	3,76E-01	1,337	0,419
MIR-552	3,79E-01	1,828	0,870
MIR-658	3,80E-01	1,468	0,554
MIR-1226*	3,80E-01	1,627	0,702
MIR-548O	3,82E-01	1,288	0,365
MIR-758	3,82E-01	1,466	0,552
MIR-548E	3,83E-01	1,299	0,377
MIR-490-5P	3,88E-01	1,405	0,490
HCMV-MIR-US33-3P	3,88E-01	1,419	0,505
MIR-567	3,89E-01	1,564	0,646
LET-7I	3,89E-01	1,343	0,425
MIR-380-5P	3,89E-01	1,334	0,416
MIR-551B*	3,95E-01	1,448	0,534
MIR-647	3,95E-01	1,474	0,560
MIR-32*	3,96E-01	1,293	0,371
MIR-1252	3,97E-01	1,317	0,397
MIR-1266	3,97E-01	2,146	1,102
MIR-1297	3,97E-01	1,293	0,370
MIR-193B*	3,99E-01	1,476	0,562
MIR-1229	3,99E-01	1,796	0,845
MIR-29C	3,99E-01	-1,355	-0,438
MIR-619	4,06E-01	1,540	0,623
MIR-1226	4,06E-01	2,366	1,243
MIR-671-5P	4,09E-01	1,402	0,487
MIR-631	4,09E-01	1,436	0,522
MIR-1251	4,10E-01	1,252	0,324

MIR-548M	4,10E-01	1,293	0,370
MIR-1250	4,11E-01	1,933	0,951
MIR-577	4,12E-01	1,558	0,640
MIR-132*	4,17E-01	1,985	0,989
MIR-512-5P	4,19E-01	1,343	0,426
MIR-1206	4,20E-01	1,282	0,358
MIR-493*	4,24E-01	2,044	1,031
MIR-449C	4,26E-01	1,969	0,977
MIR-1323	4,28E-01	1,431	0,517
MIR-518F	4,30E-01	1,642	0,716
EBV-MIR-BART20-3P	4,34E-01	1,424	0,510
MIR-1238	4,35E-01	1,809	0,856
MIR-328-3P	4,36E-01	1,305	0,384
MIR-198	4,36E-01	1,386	0,471
MIR-27A	4,38E-01	-1,286	-0,363
MIR-139-3P	4,38E-01	2,313	1,210
MIR-499-3P	4,41E-01	1,257	0,330
MIR-2110	4,43E-01	1,439	0,525
MIR-190	4,43E-01	1,452	0,538
MIR-494	4,45E-01	1,996	0,997
MIR-1468	4,48E-01	1,252	0,324
MIR-1203	4,48E-01	1,975	0,982
MIR-219-1-3P	4,51E-01	1,267	0,342
MIR-566	4,51E-01	1,412	0,497
MIR-593*	4,52E-01	1,419	0,504
MIR-196B*	4,54E-01	2,163	1,113
MIR-320D	4,55E-01	1,374	0,458
MIR-191	4,56E-01	1,304	0,383
MIR-1245	4,56E-01	1,546	0,628
MIR-484	4,59E-01	1,312	0,392
MIR-1208	4,60E-01	1,243	0,314
MIR-1302	4,62E-01	1,290	0,368
MIR-924	4,64E-01	1,305	0,384
EBV-MIR-BART3-3P	4,65E-01	1,293	0,370
MIR-597	4,69E-01	1,409	0,494
MIR-302E	4,71E-01	1,663	0,734
MIR-449B*	4,75E-01	1,782	0,834
MIR-320C	4,78E-01	1,354	0,437
MIR-636	4,83E-01	1,333	0,414
MIR-367*	4,86E-01	1,381	0,466
MIR-1257	4,87E-01	1,225	0,293
MIR-556-5P	4,88E-01	1,466	0,552
EBV-MIR-BART5*	4,88E-01	2,129	1,090
MIR-2115	4,89E-01	1,570	0,651
MIR-602	4,90E-01	1,402	0,488
MIR-1207-3P	4,96E-01	1,450	0,537
MIR-195*	5,01E-01	1,765	0,820
MIR-432*	5,07E-01	1,411	0,496
MIR-138	5,08E-01	1,339	0,421
MIR-202-5P	5,09E-01	1,289	0,366
MIR-1306-3P	5,09E-01	1,290	0,367
MIR-1A	5,11E-01	2,022	1,016
MIR-920	5,14E-01	1,569	0,650
MIR-34C-5P	5,17E-01	1,454	0,540
HSV1-MIR-H6-3P	5,18E-01	1,782	0,834
EBV-MIR-BHRF1-2*	5,18E-01	1,555	0,637
MIR-1180	5,21E-01	1,461	0,547
MIR-378	5,24E-01	1,291	0,369
MIR-378*	5,28E-01	1,314	0,394

MIR-941	5,28E-01	1,329	0,410
MIR-548Q	5,30E-01	1,445	0,531
MIR-660	5,32E-01	-1,379	-0,463
MIR-501-3P	5,35E-01	1,923	0,943
MIR-744	5,36E-01	1,231	0,299
MIR-326	5,38E-01	1,290	0,367
MIR-1268-1268B	5,39E-01	1,260	0,334
MIR-1282	5,40E-01	1,423	0,509
MIR-339-5P	5,42E-01	-1,380	-0,465
MIR-933	5,45E-01	1,284	0,361
MIR-26B*	5,47E-01	1,244	0,315
MIR-1225-5P	5,50E-01	1,343	0,425
MIR-193A-5P	5,51E-01	1,579	0,659
MIR-27B	5,51E-01	-1,321	-0,401
MIR-99B*	5,52E-01	1,548	0,631
MIR-532-5P	5,53E-01	1,435	0,521
MIR-505*	5,55E-01	1,765	0,820
MIR-1204	5,57E-01	1,955	0,967
MIR-548H	5,62E-01	1,300	0,378
MIR-1296	5,62E-01	-1,346	-0,428
MIR-1299	5,64E-01	1,347	0,430
MIR-574-5P	5,64E-01	1,391	0,476
MIR-765	5,68E-01	1,434	0,520
HSV2-MIR-H2	5,69E-01	1,799	0,847
LET-7A*-LET-7C-2*	5,70E-01	1,286	0,363
MIR-1231	5,74E-01	1,387	0,472
MIR-92B*	5,75E-01	1,308	0,388
MIR-1236	5,76E-01	1,357	0,441
MIR-15B*	5,77E-01	1,227	0,295
MIR-1224-5P	5,77E-01	1,294	0,372
MIR-100	5,78E-01	1,324	0,405
MIR-194*	5,80E-01	1,706	0,771
MIR-296-5P	5,83E-01	1,316	0,396
MIR-125B	5,83E-01	1,396	0,481
MIR-606	5,87E-01	1,528	0,611
MIR-27A*	5,91E-01	1,253	0,326
MIR-128	5,95E-01	1,258	0,331
MIR-1202	5,97E-01	1,932	0,950
MIR-1181	5,99E-01	1,557	0,639
MIR-1912	6,05E-01	1,280	0,356
MIR-374B*-374C*	6,06E-01	1,253	0,325
MIR-490-3P	6,06E-01	1,356	0,439
MIR-335*	6,07E-01	1,274	0,350
HSV1-MIR-H1	6,07E-01	1,302	0,380
MIR-1258	6,08E-01	1,258	0,331
MIR-214*	6,08E-01	1,402	0,488
MIR-19B-2*	6,10E-01	1,174	0,232
MIR-1225-3P	6,13E-01	1,584	0,663
EBV-MIR-BART9*	6,15E-01	1,680	0,748
MIR-1307	6,18E-01	1,648	0,721
MIR-92A	6,19E-01	1,413	0,499
MIR-124	6,24E-01	1,361	0,445
MIR-200C*	6,26E-01	1,562	0,643
MIR-26A-2*	6,26E-01	1,350	0,433
MIR-675*	6,29E-01	1,686	0,753
MIR-421-5P	6,31E-01	1,697	0,763
MIR-20B	6,34E-01	-1,518	-0,602
MIR-556-3P	6,36E-01	1,275	0,351
MIR-1205	6,39E-01	1,372	0,456

MIR-936	6,40E-01	1,187	0,247
MIR-922	6,47E-01	1,337	0,419
MIR-1915	6,50E-01	1,303	0,382
MIR-1263	6,50E-01	1,422	0,508
MIR-19B	6,52E-01	-1,409	-0,494
MIR-1178	6,60E-01	1,297	0,375
MIR-302D*	6,60E-01	1,470	0,556
MIR-935	6,61E-01	1,173	0,230
HSV1-MIR-H5-3P	6,66E-01	1,320	0,401
MIR-628-5P	6,69E-01	1,292	0,370
MIR-661	6,72E-01	1,307	0,386
MIR-302F	6,73E-01	1,223	0,290
MIR-1247	6,75E-01	-1,605	-0,683
MIR-621	6,77E-01	1,367	0,452
MIR-362-3P	6,81E-01	1,463	0,549
MIR-1270	6,82E-01	1,242	0,313
MIR-1262	6,87E-01	1,213	0,278
MIR-125B-2*	6,88E-01	1,668	0,738
MIR-1537	6,88E-01	1,219	0,286
MIR-1264	6,88E-01	1,350	0,433
MIR-30C	6,94E-01	-1,190	-0,251
MIR-19B-1*	6,99E-01	1,238	0,308
MIR-1294	7,15E-01	1,198	0,261
MIR-1183	7,16E-01	1,271	0,346
MIR-1265	7,16E-01	1,378	0,463
MIR-1976	7,23E-01	1,475	0,560
MIR-140-3P	7,26E-01	1,191	0,252
MIR-338-5P	7,26E-01	1,189	0,250
MIR-1197	7,26E-01	1,260	0,334
MIR-548J	7,26E-01	1,266	0,340
HSV2-MIR-H4-5P	7,28E-01	1,359	0,443
MIR-92A-1*	7,29E-01	1,179	0,238
MIR-877	7,30E-01	1,297	0,375
MIR-93	7,35E-01	1,223	0,290
MIR-425*	7,37E-01	1,288	0,365
MIR-611	7,37E-01	1,209	0,274
LET-7F-2*	7,42E-01	1,249	0,321
LET-7A	7,44E-01	1,296	0,374
MIR-503	7,46E-01	-1,300	-0,378
MIR-1272	7,47E-01	1,224	0,291
MIR-25*	7,50E-01	1,169	0,226
MIR-548K	7,50E-01	1,218	0,285
MIR-30B	7,53E-01	-1,308	-0,388
MIR-183*	7,57E-01	1,267	0,341
MIR-1284	7,71E-01	1,387	0,472
MIR-144*	7,72E-01	1,213	0,279
MIR-576-5P	7,72E-01	-1,278	-0,354
MIR-1909*	7,72E-01	1,412	0,497
LET-7D	7,74E-01	-1,142	-0,191
MIR-450B-3P	7,74E-01	1,242	0,313
MIR-1234	7,82E-01	1,350	0,433
MIR-33B	7,82E-01	1,515	0,599
MIR-322*-MIR-424*	7,84E-01	1,290	0,368
MIR-1301	7,84E-01	1,236	0,305
MIR-29A	7,84E-01	1,193	0,255
MIR-1914*	7,86E-01	1,206	0,270
MIR-30E*	7,87E-01	-1,146	-0,197
HSV1-MIR-H2	7,89E-01	1,500	0,585
MIR-1184	7,93E-01	1,476	0,562

LET-7E*	7,97E-01	1,303	0,382
MIR-224*	7,98E-01	1,520	0,604
MIR-10A*	8,01E-01	1,370	0,454
LET-7A-2*	8,01E-01	1,291	0,369
LET-7F-1*	8,04E-01	1,186	0,246
MIR-648	8,05E-01	1,280	0,356
MIR-1305	8,08E-01	1,245	0,316
MIR-30D*	8,08E-01	1,306	0,385
MIR-1324	8,09E-01	1,217	0,284
MIR-744*	8,17E-01	1,187	0,247
MIR-639	8,17E-01	1,297	0,375
EBV-MIR-BART7*	8,20E-01	1,388	0,473
MIR-942	8,20E-01	1,180	0,239
MIR-423-5P	8,22E-01	1,330	0,411
MIR-487B	8,25E-01	1,154	0,207
MIR-614	8,25E-01	1,291	0,368
EBV-MIR-BART4*	8,26E-01	1,462	0,548
MIR-1292	8,26E-01	1,212	0,277
MIR-138-2*	8,27E-01	1,206	0,271
MIR-345-5P	8,29E-01	1,176	0,234
MIR-196A*	8,30E-01	1,355	0,439
MIR-103A	8,32E-01	1,122	0,166
MIR-1539	8,36E-01	1,404	0,489
MIR-377*	8,36E-01	1,237	0,307
MIR-148B*	8,37E-01	1,419	0,505
MIR-1224-3P	8,39E-01	1,254	0,326
MIR-136*	8,39E-01	1,196	0,258
HSV2-MIR-H4-3P	8,41E-01	1,496	0,581
MIR-1269	8,42E-01	1,319	0,400
MIR-146A	8,42E-01	1,268	0,342
MIR-663B	8,43E-01	1,261	0,335
MIR-422A	8,46E-01	-1,216	-0,282
MIR-1471	8,51E-01	1,400	0,486
MIR-423-3P	8,51E-01	1,148	0,199
MIR-98	8,54E-01	1,319	0,399
MIR-20A*	8,59E-01	1,269	0,344
MIR-124*	8,67E-01	1,279	0,355
MIR-30C-2*	8,70E-01	1,273	0,348
MIR-28-5P-28C	8,71E-01	-1,241	-0,311
MIR-1911*	8,71E-01	1,435	0,521
MIR-1827	8,71E-01	1,359	0,443
MIR-593	8,75E-01	1,174	0,231
MIR-483-5P	8,79E-01	1,220	0,287
MIR-1271	8,83E-01	1,378	0,463
MIR-192*	8,84E-01	1,354	0,438
MIR-187*	8,85E-01	1,396	0,481
MIR-2116*	8,88E-01	1,303	0,382
MIR-937	8,89E-01	1,156	0,209
MIR-15A	8,93E-01	-1,196	-0,258
MIR-23A	8,93E-01	1,156	0,210
MIR-524-5P	8,94E-01	1,478	0,563
MIR-135B*	8,94E-01	1,536	0,619
MIR-193A-3P	9,01E-01	1,205	0,269
MIR-1825	9,04E-01	1,300	0,378
MIR-185*	9,05E-01	1,180	0,239
MIR-1281	9,09E-01	1,274	0,350
MIR-185	9,11E-01	1,160	0,214
LET-7B*	9,15E-01	1,397	0,482
MIR-431*	9,16E-01	1,163	0,218

MIR-29A*	9,17E-01	1,186	0,247
MIR-34A*	9,17E-01	1,154	0,206
MIR-23B*	9,18E-01	1,223	0,291
MIR-106B*	9,18E-01	1,423	0,509
MIR-24-2*	9,25E-01	1,321	0,401
MIR-99A*	9,26E-01	1,185	0,245
LET-7C	9,27E-01	1,092	0,127
MIR-135A*	9,27E-01	1,120	0,163
MIR-24	9,27E-01	1,095	0,131
MIR-15A*	9,30E-01	1,320	0,401
MIR-1911	9,30E-01	1,141	0,190
MIR-921	9,35E-01	1,241	0,312
MIR-1283	9,38E-01	1,142	0,192
MIR-219-2-3P	9,38E-01	1,180	0,238
MIR-342-3P	9,39E-01	1,088	0,121
HIV1-MIR-TAR-3P	9,41E-01	1,298	0,377
EBV-MIR-BART13*	9,42E-01	1,280	0,356
MIR-107	9,43E-01	1,071	0,099
MIR-1280	9,43E-01	-1,243	-0,314
MIR-222	9,43E-01	-1,156	-0,209
HSV1-MIR-H2*	9,44E-01	1,253	0,326
HSV1-MIR-H8*	9,45E-01	1,161	0,215
MIR-17	9,49E-01	-1,109	-0,149
MIR-554	9,52E-01	1,180	0,238
MIR-20B*	9,54E-01	-1,104	-0,142
MIR-26A	9,55E-01	1,073	0,102
MIR-218-1*	9,59E-01	1,133	0,180
MIR-720	9,59E-01	-1,101	-0,139
MIR-16	9,59E-01	-1,067	-0,093
MIR-1228	9,62E-01	-1,145	-0,196
MIR-200B*	9,62E-01	1,156	0,209
MIR-361-5P	9,64E-01	1,085	0,118
HCMV-MIR-UL70-5P	9,66E-01	1,138	0,187
MIR-1255B	9,67E-01	1,174	0,232
MIR-132	9,67E-01	1,131	0,178
MIR-150*	9,68E-01	1,199	0,262
MIR-221*	9,68E-01	1,213	0,278
MIR-221	9,70E-01	1,081	0,112
MIR-7-2*	9,72E-01	1,088	0,121
MIR-99A	9,74E-01	1,132	0,179
MIR-322-MIR-424	9,75E-01	-1,194	-0,256
MIR-106B	9,75E-01	-1,107	-0,147
MIR-34A	9,76E-01	-1,092	-0,127
MIR-33B*	9,76E-01	1,073	0,102
MIR-1287	9,76E-01	1,103	0,141
LET-7G	9,77E-01	1,065	0,091
HSV1-MIR-H3	9,77E-01	1,118	0,161
MIR-18A	9,77E-01	-1,106	-0,146
MIR-1295	9,79E-01	1,078	0,108
MIR-1278	9,80E-01	1,098	0,135
MIR-625*	9,80E-01	1,145	0,195
MIR-1200	9,81E-01	1,168	0,225
MIR-15B	9,81E-01	1,058	0,082
MIR-130A*	9,82E-01	1,105	0,144
MIR-148B	9,83E-01	-1,109	-0,149
MIR-146A*	9,83E-01	1,104	0,143
MIR-1538	9,85E-01	-1,106	-0,146
MIR-28-3P	9,85E-01	1,085	0,118
MIR-1237	9,86E-01	1,109	0,150

MIR-138-1*	9,86E-01	1,152	0,204
MIR-590-3P	9,87E-01	1,097	0,133
MIR-127*	9,87E-01	1,107	0,147
HSV1-MIR-H7*	9,87E-01	1,134	0,181
BKV-MIR-B1-5P	9,88E-01	1,109	0,149
MIR-1285	9,89E-01	1,097	0,134
MIR-940	9,90E-01	-1,088	-0,121
MIR-583	9,91E-01	1,094	0,129
MIR-1233	9,91E-01	-1,113	-0,154
MIR-1910	9,91E-01	1,124	0,169
MIR-1267	9,91E-01	1,073	0,102
MIR-548D-5P	9,92E-01	1,083	0,115
MIR-1913	9,93E-01	-1,102	-0,140
MIR-361-3P	9,93E-01	-1,116	-0,158
MIR-1273	9,93E-01	1,066	0,093
MIR-34C-3P	9,93E-01	1,077	0,106
LET-7G*	9,94E-01	-1,071	-0,099
MIR-1253	9,95E-01	1,080	0,111
MIR-146B-5P	9,95E-01	1,040	0,057
MIR-331-3P	9,96E-01	-1,048	-0,068
MIR-30D	9,96E-01	1,039	0,056
MIR-664-3P	9,97E-01	1,058	0,081
MIR-223	9,97E-01	-1,030	-0,043
MIR-105*	9,99E-01	1,051	0,071
MIR-122*	9,99E-01	1,039	0,056
MIR-92B	9,99E-01	1,032	0,045
MIR-21*	9,99E-01	1,081	0,113
MIR-19A	9,99E-01	-1,038	-0,054
MIR-342-5P	9,99E-01	1,085	0,118
MIR-106A	9,99E-01	-1,018	-0,025
MIR-425	9,99E-01	-1,022	-0,032
MIR-210	9,99E-01	-1,037	-0,053
MIR-148A*	1,00E+00	1,023	0,032
MIR-125B-1*	1,00E+00	-1,018	-0,026
MIR-1249	1,00E+00	1,022	0,032
MIR-22	1,00E+00	1,020	0,028
MIR-18B	1,00E+00	-1,014	-0,021
MIR-1972	1,00E+00	-1,022	-0,031
MIR-155*	1,00E+00	1,011	0,015
MIR-145*	1,00E+00	1,010	0,014
EBV-MIR-BART10*	1,00E+00	1,017	0,024
MIR-568	1,00E+00	1,010	0,014
MIR-1915*	1,00E+00	1,012	0,018
MIR-1914	1,00E+00	1,009	0,013
MCV-MIR-M1-3P	1,00E+00	1,003	0,005
MIR-943	1,00E+00	1,002	0,003
MIR-1470	1,00E+00	1,001	0,002
